# FKBP51 promotes invasion and migration by increasing the autophagic degradation of TIMP3 in clear cell renal cell carcinoma

**DOI:** 10.1038/s41419-021-04192-8

**Published:** 2021-10-01

**Authors:** Shaowei Mao, Di Zhang, Luan Chen, Jie Tan, Yunpeng Chu, Sijia Huang, Wenqi Zhou, Hengwei Qin, Qinghua Xia, Yueran Zhao, Rongxiu Li, Shengying Qin, Muyun Wei

**Affiliations:** 1 School of Life Sciences and Biotechnology, Shanghai Jiao Tong University, 800 Dong Chuan Road, Shanghai, China; 2 State Key Laboratory of Microbial Metabolism, School of Life Sciences and Biotechnology, Shanghai Jiao Tong University, 800 Dong Chuan Road, Shanghai, China; 3 Bio-X Institutes, Key Laboratory of the Genetics of Developmental and Neuropsychiatric Disorders (Ministry of Education), Shanghai Jiao Tong University, 800 Dong Chuan Road, Shanghai, China; 4 School of Medicine, Shanghai Jiao Tong University, 800 Dong Chuan Road, Shanghai, China; 5 Shandong Provincial Hospital, 324 Jingwu Road, Jinan, China

**Keywords:** Renal cell carcinoma, Extracellular matrix

## Abstract

The occurrence of metastasis is a serious risk for renal cell carcinoma (RCC) patients. In order to develop novel therapeutic approaches to control the progression of metastatic RCC, it is of urgent need to understand the molecular mechanisms underlying RCC metastasis and identify prognostic markers of metastatic risk. Matrix metalloproteinases (MMPs) and tissue inhibitors of metalloproteinases (TIMPs) have been known to be closely associated with extracellular matrix (ECM) turnover, which plays a highly active role in tumor metastasis. Recent studies have shown that immunophilin FK-506-binding protein 51 (FKBP51) may be important for the regulation of ECM function, and exert effects on the invasion and migration of tumor cells. However, the mechanisms underlying these activities remain unclear. The present study detected the role of FKBP51 in clear cell renal cell carcinoma (ccRCC), the most common subtype of RCC, and found that FKBP51 significantly promotes ccRCC invasion and migration by binding with the TIMP3, connecting TIMP3 with Beclin1 complex and increasing autophagic degradation of TIMP3. Given the important roles that TIMPs/MMPs play in ECM regulation and remodeling, our findings will provide new perspective for future investigation of the regulation of metastasis of kidney cancer and other types of cancer.

## Introduction

Renal cell carcinoma (RCC) is one of the most common cancers globally, with 403,262 new cases having been diagnosed and 175,098 people having died from the disease in 2018 [1]. Clear-cell renal cell carcinoma (ccRCC) is the most prevalent subtype of RCC with a frequency of 70–80%, followed by papillary RCC, chromophobe RCC, carcinoma of the collecting ducts of Bellini, and unclassified RCC [2]. Approximately 20–30% of RCC patients were diagnosed with synchronous metastases at the time of presentation, meanwhile 20% of patients who undergo nephrectomy still suffered relapse and developed metastatic RCC [3]. Therefore, understanding the molecular mechanisms of RCC metastasis and identifying effective prognostic markers for the individual metastatic risk are urgently needed to develop novel therapeutic approaches to control and treat metastatic RCC.

Matrix metalloproteinases (MMPs) are a family of zinc-dependent endopeptidases that control the degradation of a wide spectrum of extracellular matrix-associated (ECM) and cell surface-associated proteins. MMPs (MMP1–28) are widely expressed during tumor progression and metastasis [4, 5]. The influence of MMP7 and MMP9 on RCC metastasis has been previously observed. Serum levels of MMP7 were significantly increased in metastatic RCC, and high MMP7 levels were independently associated with shorter disease-specific survival [6–8]. MMP9 has been indicated to be a negative prognostic factor in RCC [9, 10] and an essential factor for vasculogenic mimicry formation and tumor metastasis in ccRCC [11, 12]. Tissue inhibitors of metalloproteinases (TIMP1–4) are proteins that form noncovalent complexes with MMPs and inhibit their activity. Among the four TIMPs, TIMP3 has preserved its dual, broader MMP inhibitory capacity and is widely localized in the ECM [13]. MMP7 and MMP9 were proved as targets of TIMP3. The regulation of TIMP3 and MMP7, MMP9 activities may be important for the invasive and migratory ability of ccRCC.

FK506-binding protein 51 (FKBP51) is a high molecular weight immunophilin encoded by the *FKBP5* gene. In addition to participating in the regulation of steroid hormones responses and hormone receptor activities, FKBP51 has been reported as both a tumor suppressor and promoter in different types of cancers [14]. This protein inhibits lung adenocarcinoma, non-small-cell lung cancer (NSCLC), pancreatic cancer and glioma via its scaffolding function that negatively regulates AKT phosphorylation [15–18]. However, clinical studies on colorectal cancer, oral squamous cell carcinoma (OSCC), and glioma reported that the upregulation of FKBP51 was associated with worse prognosis and metastasis [19–21]. Researchers also found that FKBP51 acts as a cofactor of the IκB-kinase (IKK) complex, induces the activation of the NF-κB pathway and promotes the progression of prostate cancers, melanoma, and glioblastoma [22–25]. Similarly, our team has previously reported that FKBP51 promotes endometrial cancer and thyroid cancer and plays a role in the remodeling process of endometrial decidualization [26, 27]. These findings suggest that FKBP51 may play an important role in the regulation of ECM functions and influence the invasion and migration of tumor cells. However, the exact roles and associated mechanisms underlying the effects of FKBP51 in ccRCC remain unknown. In this study, we reported high-level expression of FKBP51 in both primary ccRCC tumor samples and cell lines and discovered that the invasion and migration ability of ccRCC cells were promoted by the FKBP51-mediated autophagic degradation of TIMP3.

## Results

### FKBP51 is frequently upregulated in ccRCC and high FKBP51 expression positively correlates with metastasis

**Fig. 1:**
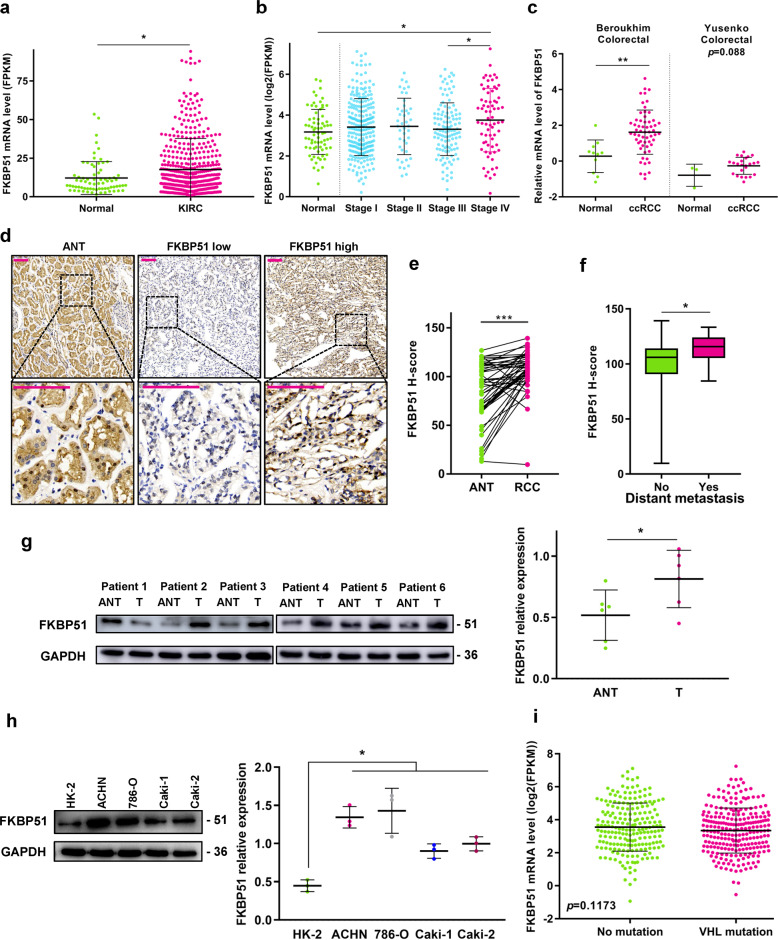
FKBP51 expression profile and its association with cancer in ccRCC patients and cell lines. **a** Comparison of the mRNA expression of FKBP51 in the normal and KIRC (ccRCC) groups in the TCGA-kidney renal clear cell carcinoma (TCGA–KIRC) cohort (normal tissue = 72, ccRCC tissue = 538). **b** Comparison of the mRNA expression of FKBP51 in the normal group and different stages of KIRC in the TCGA–KIRC cohort. **c** Comparison of the mRNA expression of FKBP51 in the normal and ccRCC groups in two Oncomine datasets: Berouklim colorectal (normal tissue = 11, ccRCC tissue = 59) and Yusenko colorectal (normal tissue = 3, ccRCC tissue = 24). **d** Examination of FKBP51 protein expression levels in ccRCC (*n* = 70) and adjacent nontumor tissues (ANT) (*n* = 49) by immunohistochemical (IHC) staining (top panel: magnification ×100; bottom panel: magnification ×400; scale bar = 100 μm). Samples were divided into two groups (FKBP51 low and high) by medium based on the *H*-score of each sample. **e** Comparison of FKBP51 protein expression levels (*H*-score) in 49 paired ANT and ccRCC tissues. **f** Comparison of FKBP51 protein expression levels in ccRCC patients with (*n* = 22) and without distant metastasis (*n* = 48). **g** FKBP51 levels in six paired ccRCC tissues (T) and matched ANTs were detected by western blotting. Schematic representation of quantitative data of the indicated proteins. Representative images from three independent experiments are shown. **h** Analysis of the FKBP51 protein expression level in the human kidney proximal tubular cell line (HK-2) and four RCC cell lines (ACHN, 786-O, Caki-1, and Caki-2) was performed by western blotting. Schematic representation of quantitative data of the indicated proteins. Representative images from three independent experiments are shown. **i***FKBP51* mRNA levels in ccRCC tissues with *VHL* mutation (*n* = 244) and without *VHL* mutation (*n* = 207). Data were collected from TCGA–KIRC cohort. *p < 0.05; **p < 0.01; ***p < 0.001. Error bars represent standard deviation.

To investigate the potential role of FKBP51 in ccRCC progression, we first evaluated the mRNA expression levels of FKBP51 in TCGA and two Oncomine colorectal datasets. As shown in Fig. 1a–c, FKBP51 expression was significantly upregulated in cancer tissues (*p* < 0.05) and increased with clinical stage (*p* < 0.05). Immunohistochemistry (IHC) staining of FKBP51 in 70 ccRCC samples and 49 adjacent normal tissues showed that the protein levels of FKBP51 were significantly higher in ccRCC tissues than in adjacent nontumorous tissues (ANTs) (*p* < 0.001, Fig. 1d, e), and upregulated in patients who developed distant metastasis compared to patients without distant metastasis (*p* < 0.05, Fig. 1f and Table 1). Western blotting assays of FKBP51 in six paired ccRCC tissues and ANTs revealed that in most cases the FKBP51 levels were higher in tumor tissues than in normal tissues (Fig. 1g). In addition, FKBP51 expression was more abundant in four RCC cell lines, ccRCC cell lines 786-O and Caki-1 and papillary RCC cell lines ACHN and Caki-2, than in the human kidney proximal tubular cell line (HK-2) (Fig. 1h). Among two ccRCC cell lines, Caki-1 expresses the wild-type von Hippel–Lindau (VHL) tumor-suppressor protein, while 786-O does not [28]. We analyzed the gene variants and mRNA expression data from the TCGA–KIRC cohort and found that mutations in *VHL* had no significant association with FKBP51 expression levels (Fig. 1i). These results indicated that the 786-O and Caki-1 cell lines were suitable to further explore the potential mechanism underlying the biological functions of FKBP51 in ccRCC cells.

**Table 1:** Correlations between FKBP51, TIMP3, MMP7 and MMP9 expression and clinical characteristics.

Clinicopathologic parameters	Total (*n* = 70)	FKBP51 expression		*p* value	TIMP3 expression		*p* value	MMP7 expression		*p* value	MMP9 expression		*p* value
		Low	High		Low	High		Low	High		Low	High	
*Age (years)*													
<65	55	26	29	0.382	27	28	0.771	28	27	0.771	26	29	0.382
≥65	15	9	6		8	7		7	8		9	6	
*Gender*													
Male	48	22	26	0.303	26	22	0.303	24	24	1	24	24	1
Female	22	13	9		9	13		11	11		11	11	
*Differentiation grade*													
1, 1–2	18	12	6	0.201	9	9	0.935	12	6	0.024*	9	9	0.935
2	43	20	23		21	22		22	21		22	21	
2–3, 3	9	3	6		5	4		1	8		4	5	
*Tumor size (cm)*													
<5	47	24	23	0.799	22	25	0.445	26	21	0.203	24	23	0.799
≥5	23	11	12		13	10		9	14		11	12	
*Distant metastasis*													
Yes	22	6	16	0.010*	15	7	0.039*	8	14	0.122	25	23	0.607
No	48	29	19		20	28		27	21		10	12	

*p value < 0.05.

^a^Using median *H*-score values as cutoff.

### Activation of FKBP51 facilitates ccRCC invasion and migration in vitro

**Fig. 2:**
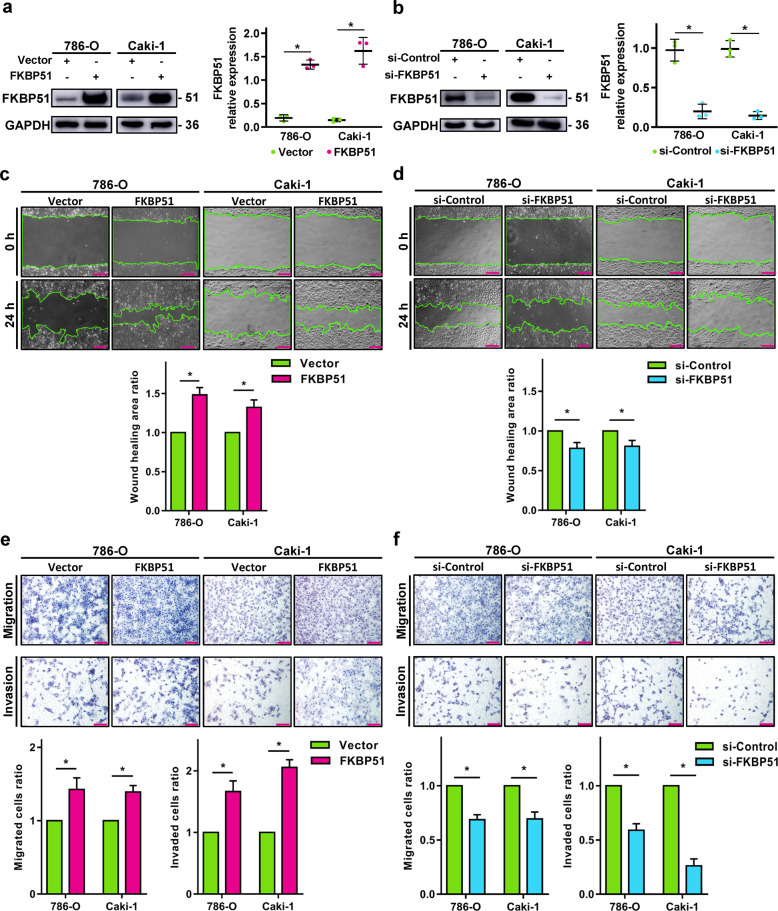
Activation of FKBP51 facilitates ccRCC invasion and migration in vitro. For FKBP51 overexpression, 786-O and Caki-1 cells were transfected with FKBP51 overexpression lentivirus (FKBP51) or empty vector lentivirus (Vector). For FKBP51 knockdown, 786-O and Caki-1 cells were transfected with siRNA-FKBP51 (si-FKBP51) or siRNA-control (si-Control). **a** The effects of FKBP51 overexpression were measured by western blotting. Schematic representation of the quantitative data of indicated proteins. Representative images from three independent experiments are shown. **b** The effects of FKBP51 knockdown were measured by western blotting. Schematic representation of quantitative data of the indicated proteins. Representative images from three independent experiments are shown. **c** FKBP51 overexpressing 786-O and Caki-1 cells were subjected to a scratch assay in serum-free media for 24 h. Empty areas in captured images were measured by ImageJ (scale bar = 200 μm). **d** FKBP51 knockdown 786-O and Caki-1 cells were subjected to a scratch assay in serum-free media for 24 h. Empty areas in captured images were measured by ImageJ (scale bar = 200 μm). **e** The migratory and invasive abilities of FKBP51 overexpressing 786-O and Caki-1 cells were assessed by transwell assay. The cells in three randomly chosen fields were counted. Representative images (scale bar = 100 μm) and corresponding statistical results are presented by the histogram. Error bars represent standard deviation. **f** The migratory and invasive abilities of FKBP51 knockdown 786-O and Caki-1 cells were assessed by transwell assay. The cells in three randomly chosen fields were counted. Representative images (scale bar = 100 μm) and corresponding statistical results are presented by the histogram. Error bars represent standard deviation. *p < 0.05.

To investigate whether FKBP51 can alter ccRCC cell tumor biology, we assayed its effects on proliferation, cell cycle, migration, and invasion in 786-O and Caki1 cell lines stably overexpressing FKBP51 or with FKBP51 knockdown. Overexpression and knockdown efficiencies were confirmed by western blotting (Fig. 2a, b). The results of flow cytometry and CCK8 assays showed that FKBP51 expression had no significant influence on the ccRCC cell cycle (Fig. S1a) or cell proliferation (Fig. S1b, c). However, as shown in the scratch assay (Fig. 2c, d) and transwell assay (Fig. 2e, f), the overexpression of FKBP51 markedly promoted the invasion and migration ability of both 786-O and Caki-1 cells while the knockdown significantly inhibited that of both cell lines. These results suggest that activation of FKBP51 facilitates ccRCC invasion and migration in vitro.

### Dissection of the mechanisms involved in FKBP51-induced ccRCC invasion and migration

**Fig. 3:**
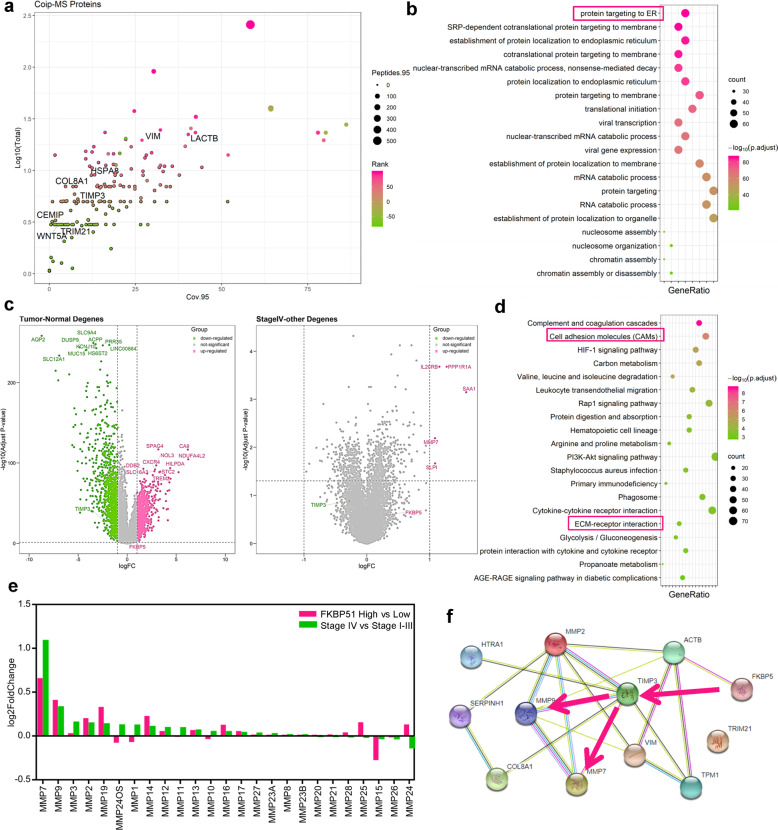
Bioinformatic analysis of Co-IP–MS data and RNA-Seq data from TCGA. **a** Scatter plot showing the 257 proteins identified by Co-IP–MS. **b** Dot plot showing the biological processes, cellular components, and molecular functions in which the 257 proteins from Co-IP–MS analysis were enriched by GO enrichment analysis. **c** Volcano plot showing the differentially expressed genes (DEGs) between tumor (*n* = 538) and normal (*n* = 72) tissues (left panel) and between stage IV (*n* = 70) and stage I–III (*n* = 377) groups (right panel). **d** Dot plot showing the KEGG pathway enrichment results for DEGs between the tumor and normal groups. **e** Bar plot showing the log2-fold changes for members of the MMP protein family in comparison between the FKBP51 high group (*n* = 269) and the FKBP51 low group (*n* = 269) (divided by the median in the tumor group) and between the stage IV group (*n* = 70) and the stage I–III group (*n* = 377). **f** Protein–protein interaction (PPI) network of metastasis-related genes from our analysis using the STRING database. The red line indicates our hypothesis for the regulatory axes in ccRCC.

To decipher the mechanisms through which FKBP51 promotes ccRCC invasion and migration, we first evaluated the levels of proteins associated with the AKT and NF-κB signaling pathways and found no significant changes in the activities of these two pathways (Fig. S1d, e). This suggested that neither NF-κB nor AKT signaling is crucial for the FKBP51-mediated promotion of ccRCC cell invasion and migration, and that other unreported mechanisms must be involved. Using Co-IP-coupled mass spectrometry (Co-IP–MS), we identified 257 proteins that interact with FKBP51 in 786-O cell lines (Fig. S2). Some of the 257 proteins were reported to be associated with regulatory effects in cancer (LACTB, WNT5A, and TRIM21) and some were associated with metastasis (TIMP3, COL8A1, VIM, and CEMIP) (Fig. 3a). GO enrichment analysis showed that these proteins were most significantly enriched on proteins targeting the estrogen receptor (ER) (Fig. 3b), which is consistent with the known roles of FKBP51 in binding with ER, suggesting that Co-IP–MS worked well in detecting FKBP51-interacting proteins. Among these proteins, we identified TIMP3 as a potential key factor in FKBP51-related metastasis, considering its ability to directly inhibit MMPs and its role in ECM degradation and remodeling. String and PPI network analysis also revealed that TIMP3 was involved in the top 15 significant nodes of the network (Figs. S3 and S4). Differentially expressed genes (DEGs) analysis of TCGA data showed that the expression of TIMP3 was downregulated and that of FKBP51 was upregulated in ccRCC and stage IV samples, while MMP7 expression was significantly increased in stage IV samples (Fig. 3c). KEGG enrichment analysis showed that DEGs between ccRCC and normal tissues were enriched in cell adhesion molecules and ECM receptor interactions (Fig. 3d). We also observed changes in the expression levels of MMP family members in TCGA samples, and found that MMP7 and MMP9, both of which are targets of TIMP3, were upregulated in the stage IV group and FKBP51 high expression group, while MMP20 and MMP21, which are not TIMP3 targets, showed no clear trend (Fig. 3e). These results suggested that TIMP3 may play key roles in FKBP51-related metastasis through the regulation of MMP7 and MMP9 (Fig. 3f).

### FKBP51 decreased the expression of TIMP3 and increased that of MMP7 and MMP9 in ccRCC cell lines and clinical samples

**Fig. 4:**
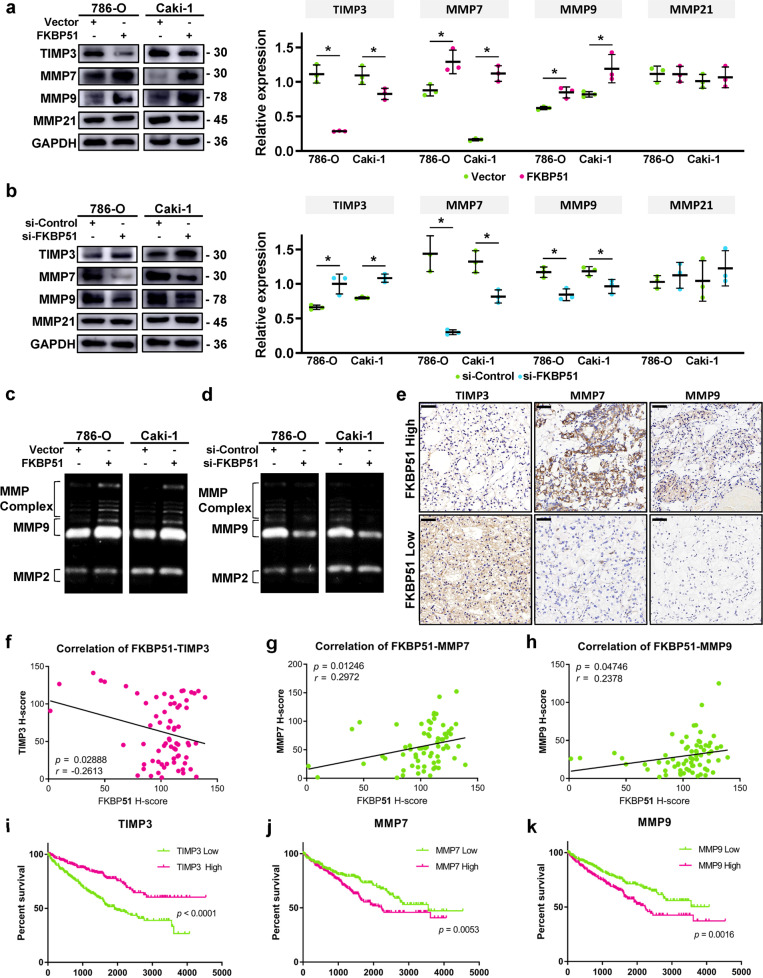
FKBP51 inhibits TIMP3 and upregulates MMP7 and MMP9 in ccRCC cell lines and tissues. For FKBP51 overexpression, 786-O and Caki-1 cells were transfected with FKBP51 overexpression lentivirus (FKBP51) or empty vector lentivirus (Vector). For FKBP51 knockdown, 786-O and Caki-1 cells were transfected with siRNA-FKBP51 (si-FKBP51) or siRNA-control (si-Control). **a** Western blotting was conducted to determine the expression levels of TIMP3 and MMPs in FKBP51 overexpressing 786-O and Caki-1 cells. Schematic representation of quantitative data of the indicated proteins. Representative images from three independent experiments are shown. **b** Western blotting was conducted to determine the expression levels of TIMP3 and MMPs in FKBP51 knockdown 786-O and Caki-1 cells. Schematic representation of quantitative data of the indicated proteins. Representative images from three independent experiments are shown. **c** The degradation activity of MMP2, MMP9, and MMPs complex was detected by gelatin zymography in FKBP51 overexpressing 786-O and Caki-1 cells. **d** The degradation activity of MMP2, MMP9 and MMPs complex was detected by gelatin zymography in FKBP51 knockdown 786-O and Caki-1 cells. **e** Immunohistochemistry was used to evaluate the expression of TIMP3, MMP7 and MMP9 in the clinical ccRCC tissues we collected (*n* = 70). Representative pictures in the low or high FKBP51 expression groups (divided by the median H-score) are displayed here. Scale bar = 50 μm. **f–h** Statistical correlation plots of the FKBP51 *H*-score and TIMP3, MMP7, and MMP9 H-scores in IHC staining are presented. **i–k** Kaplan–Meier survival curves of ccRCC patients with low or high expression of TIMP3, MMP7, and MMP9 in the TCGA cohort (*n* = 538). *p < 0.05.

To understand the relationship between FKBP51 and TIMP3, we first assessed whether TIMP3, MMP7 and MMP9 were regulated in FKBP51 overexpressing and knockdown ccRCC cell lines. Western blotting results showed that FKBP51 overexpression decreased the expression of TIMP3 while increasing that of its targets, MMP7 and MMP9 (Fig. 4a); FKBP51 knockdown increased the expression of TIMP3 and decreased that of MMP7 and MMP9 (Fig. 4b). Additionally, no difference was observed in the expression of MMP21. In addition, as shown in Fig. 4c, d, FKBP51 upregulation increased, and downregulation decreased the zymographic activity of MMP9 and some of MMPs complex. IHC staining in clinical ccRCC tissues (*n* = 70) confirmed that patients with high levels of FKBP51 expression displayed lower TIMP3, and higher MMP7 and MMP9 expression levels (Fig. 4e). The *H*-scores of FKBP51 were negatively correlated with those of TIMP3 (*r* = −0.2613, *p* = 0.02888) (Fig. 4f) and positively correlated with those of MMP7 (*r* = 0.2972, *p* = 0.01246) (Fig. 4g) and MMP9 (*r* = 0.2378, *p* = 0.04746) (Fig. 4h). Furthermore, Kaplan–Meier survival curves showed that lower levels of TIMP3 (*p* < 0.0001) (Fig. 4i), and higher levels of MMP7 (*p* = 0.0053) (Fig. 4j) and MMP9 (*p* = 0.0016) (Fig. 4k) were significantly correlated with shorter overall survival using TCGA data (*n* = 538).

### FKBP51 directly interacts with TIMP3 and decreases TIMP3 protein levels

**Fig. 5:**
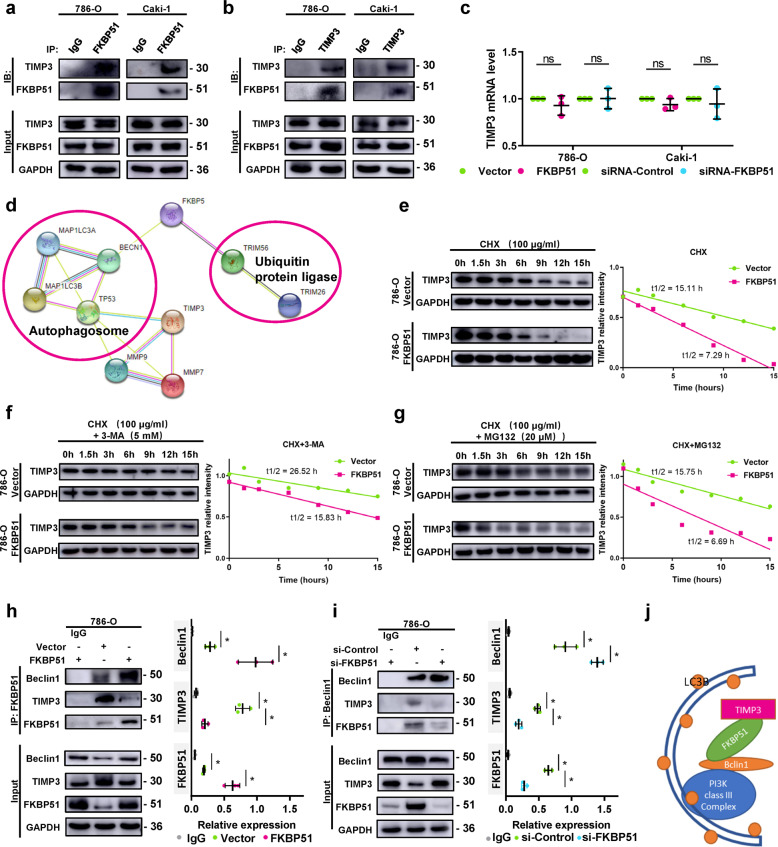
FKBP51 interacts with TIMP3 and promotes its autophagic degradation. **a** Co-IP experiments in 786-O and Caki-1 cells using an anti-FKBP51 antibody with IgG as a control. **b** Co-IP experiments in 786-O and Caki-1 cells using an anti-TIMP3 antibody with IgG as a control. **c** Real-time quantitative PCR to evaluate TIMP3 mRNA levels in both 786-O and Caki-1 cell lines with FKBP51 overexpression or FKBP51 knockdown. **d** Proteins possibly involved in FKBP51 and TIMP3 related autophagy and ubiquitination in the PPI network based on the STRING database. **e**–**g** 786-O cells transfected with FKBP51 overexpression lentivirus (FKBP51) and empty vector lentivirus (Vector) were treated with 100 μg/ml cycloheximide (CHX) alone, with 5 mM 3-methyladenine (3-MA) or with 20 μM MG132 and harvested at 0, 1.5, 3, 6, 9, 12, and 15 h. Lysates were subjected to western blotting to detect the changes in TIMP3 levels (TIMP3: 30 kD, GAPDH: 36 kD). Band intensities of the blots were analyzed by ImageJ and plotted using GraphPad Prism. **h** Co-IP experiments in 786-O cells transfected with FKBP51 overexpress lentivirus (FKBP51) and empty vector lentivirus (Vector) using an anti-FKBP51 antibody with IgG as a control. Schematic representation of quantitative data of the indicated proteins. Representative images from three independent experiments are shown. **i** Co-IP experiments in 786-O cells transfected with siRNA-FKBP51 (si-FKBP51) and siRNA-Control (si-Control) using an anti-Beclin1 antibody with IgG as a control. Schematic representation of quantitative data of the indicated proteins. Representative images from three independent experiments are shown. **j** The hypothesis of how TIMP3/FKBP51/Beclin1 works in autophagy. *p < 0.05.

Co-IP results indicated that endogenous FKBP51 and TIMP3 interact with each other in 786-O and Caki-1 cells (Fig. 5a, b). Real-time quantitative PCR showed no significant change in TIMP3 mRNA levels in either the 786-O or Caki-1 cell lines with FKBP51 overexpression or knockdown (Fig. 5c). These observations suggested that FKBP51 may regulate TIMP3 at the posttranscriptional level, which also explained the absence of a clear correlation between FKBP51 and TIMP3 mRNA levels in the TCGA database (Fig. S5a). We then focused on the potential mechanism through which FKBP51 may affect the protein stability of TIMP3. FKBP51 has been reported to bind with Beclin1, thus participating in autophagy and LC3A/B conversion [29], as well as to interact with the E3 ubiquitin ligase TRIM56 in a chaperone-cochaperone-client interaction network [30]. These interactions may provide explanations for the FKBP51-induced decrease in TIMP3 expression (Fig. 5d).

### FKBP51 promotes the autophagic degradation of TIMP3 through its interaction with Beclin1

To determine whether FKBP51 decreases the stability of TIMP3, 786-O cells transfected with empty vector or with FKBP51 overexpressing lentivirus were treated with 100 μg/ml cycloheximide, a protein synthesis inhibitor. As shown in Fig. 5e, overexpression of FKBP51 markedly decreased the half-life of TIMP3 from 15.11 h (CHX + Vector) to 7.29 h (CHX + FKBP51), suggesting that FKBP51 decreased the stability of TIMP3. We further used 5 mM 3-methyladenine (3-MA), an autophagy inhibitor, and 20 μM MG132, a proteasome inhibitor, to test whether the degradation of TIMP3 is based on autophagy or ubiquitination. After treatment with 3-MA, the half-life of TIMP3 was increased to 26.52 h (3-MA + CHX + Vector) and 15.83 h (3-MA + CHX + FKBP51) (Fig. 5f), while treatment with MG132 showed no clear influence on the half-life of TIMP3 (Fig. 5g). These results showed that overexpressing FKBP51 increases the autophagic degradation of the TIMP3 protein, and this effect can be blocked by exposure to the autophagy inhibitor, 3-MA. Given that FKBP51 is involved in autophagy regulation through binding to Beclin1, a central component of the PtdIns3K complex that exists on the inner side of forming the autophagosome membrane, we suspected that TIMP3 may bind with FKBP51 and Beclin1 and therefore be translocated into the autophagosome and then be degraded. Co-IP experiments in 786-O cell lines confirmed the physical interaction of Beclin1 and TIMP3 with FKBP51 (Fig. 5h). Moreover, knockdown of FKBP51 in 786-O cells blocked the interaction between Beclin1 and TIMP3 (Fig. 5i). These results led us to hypothesize that FKBP51 promotes TIMP3 autophagic degradation by acting as a “bridge” to connect TIMP3 with Beclin1 and autophagy (Fig. 5j).

**Fig. 6:**
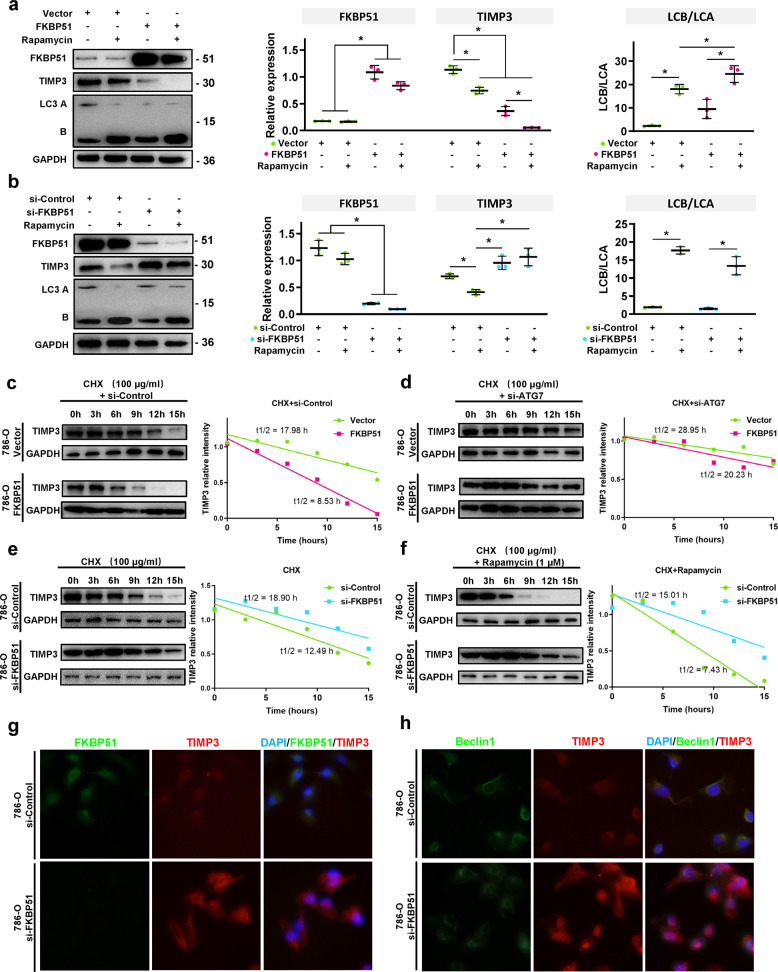
FKBP51 interacts with TIMP3 and promotes its autophagic degradation by connecting it with the Beclin1 complex. **a** Western blotting was used to determine the expression of FKBP51, TIMP3, and LC3A/B in 786-O cells transfected with FKBP51 overexpression lentivirus (FKBP51) and empty vector lentivirus (Vector) treated with or without 1 μM rapamycin. Schematic representation of quantitative data of the indicated proteins. Representative images from three independent experiments are shown. **b** Western blotting was used to determine the expression of FKBP51, TIMP3, and LC3A/B in 786-O cells transfected with siRNA-FKBP51 (si-FKBP51) and siRNA-Control (si-Control) treated with or without 1 μM rapamycin. Schematic representation of quantitative data of the indicated proteins. Representative images from three independent experiments are shown. **c**, **d** 786-O cells transfected with FKBP51 overexpression lentivirus (FKBP51) and empty vector lentivirus (Vector) were treated with 100 μg/ml cycloheximide (CHX) with siRNA-Control (si-Control) or siRNA-ATG7 (si-ATG7) and harvested at 0, 3, 6, 9, 12, and 15 h. Lysates were subjected to western blotting to detect the changes in TIMP3 levels (TIMP3: 30 kD, GAPDH: 36 kD). Band intensities of the blots were analyzed by ImageJ and plotted using GraphPad Prism. **e**, **f** 786-O cells transfected with siRNA-FKBP51 (si-FKBP51) and siRNA-Control (si-Control) were treated with 100 μg/ml cycloheximide (CHX) with or without 1 μM rapamycin and harvested at 0, 3, 6, 9, 12, and 15 h. Lysates were subjected to western blotting to detect the changes in TIMP3 levels (TIMP3: 30 kD, GAPDH: 36 kD). Band intensities of the blots were analyzed by ImageJ and plotted using GraphPad Prism. **g** Fluorescence staining of FKBP51 (green) and TIMP3 (red) in 786-O cells transfected with siRNA-FKBP51 (si-FKBP51) and siRNA-Control (si-Control). Magnification ×400. **h** Fluorescence staining of Beclin1 (green) and TIMP3 (red) in 786-O cells transfected with siRNA-FKBP51 (si-FKBP51) and siRNA-Control (si-Control). Magnification ×400. *p < 0.05.

To verify our hypothesis, we treated FKBP51 overexpressing and knockdown 786-O cells with rapamycin, an inducer of autophagy, to observe the changes in TIMP3 expression and LC3 B/A turnover. As shown in Fig. 6a, rapamycin efficiently increased the flux of autophagy (LC3B/A rate) and decreased the level of TIMP3, while FKBP51 overexpression promoted TIMP3 degradation. The knockdown of FKBP51 decreased the degradation of TIMP3 and showed no remarkable influence on autophagy flux induced by rapamycin (Fig. 6b). By using CHX with or without siRNA-ATG7 in FKBP51 overexpressing 786-O cells, we found that the promotion of TIMP3 degradation induced by FKBP51 overexpression could be blocked by siRNA-ATG7, and the half-life of TIMP3 increased from 8.53 h (si-Control + CHX + FKBP51) to 20.23 h (si-ATG7 + CHX + FKBP51) (Fig. 6c, d). In addition, by using CHX with or without rapamycin in FKBP51 knockdown 786-O cells, we found that induction by rapamycin promoted the degradation of TIMPs in 786-O cells treated with siRNA-Control (from 12.49 to 7.43 h), and this promotion could be blocked by siRNA-FKBP51 (Fig. 6e, f). The efficiency of all the interventions, including 3-MA, rapamycin, and siRNA-ATG, was tested before performing the experiments above (Fig. S5b). In addition, confocal microscopy analysis revealed the colocalizations of FKBP51 and TIMP3 and Beclin1 and TIMP3 in 786-O cells, while the knockdown of FKBP51 increased the expression of TIMP3 and blocked the colocalization of Beclin1 and TIMP3 (Fig. 6g, h). All the results above strongly support our model that FKBP51 acts as a “bridge” connecting TIMP3 with the Beclin1 complex and promotes TIMP3 degradation.

### TIMP3 autophagic degradation is essential for the FKBP51-induced invasion and migration of ccRCC cells

**Fig. 7:**
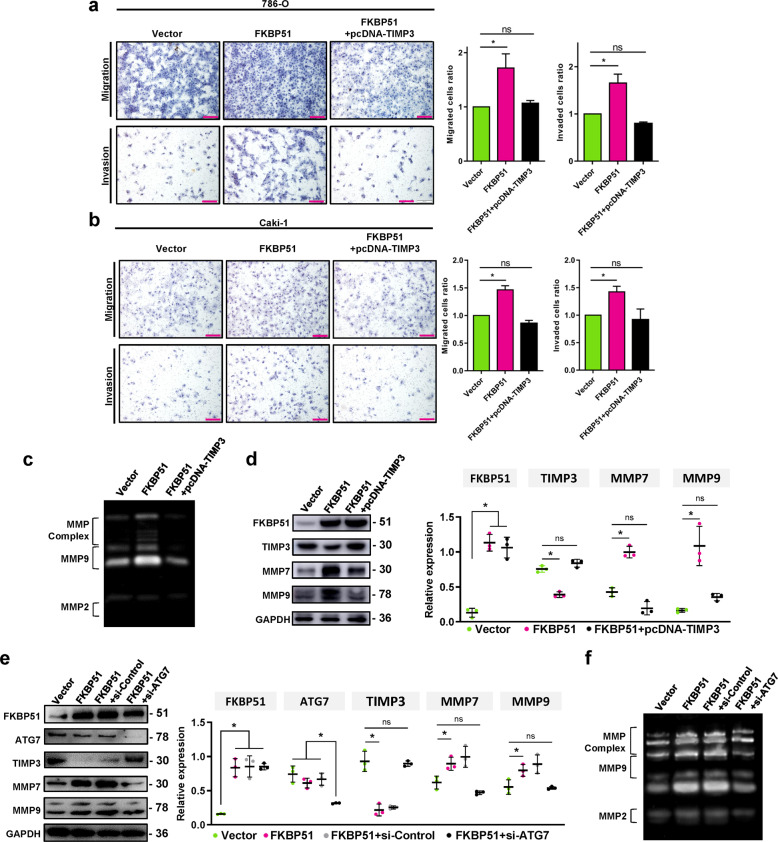
TIMP3 autophagic degradation is required for FKBP51-induced invasion and migration. **a**, **b** Transwell assays were conducted to analyze the migratory and invasive abilities of FKBP51-overexpressing 786-O and Caki-1 cells transfected with or without pcDNA3.1-TIMP3. **c** The degradation activity of MMP2, MMP9 and MMPs complex was detected by gelatin zymography in FKBP51 overexpressing 786-O cells transfected with or without pcDNA3.1-TIMP3. **d** Western blotting was used to detect the expression levels of TIMP3, MMP7 and MMP9 in FKBP51 overexpressing 786-O cells transfected with or without pcDNA3.1-TIMP3. Schematic representation of quantitative data of the indicated proteins. Representative images from three independent experiments are shown. **e** Western blotting was used to detect the expression levels of FKBP51, ATG7, TIMP3, MMP7, and MMP9 in FKBP51 overexpressing 786-O cells transfected with siRNA-Control (si-Control) or siRNA-ATG7 (si-ATG7). **f** The degradation activity of MMP2, MMP9, and MMPs complex was detected by gelatin zymography in FKBP51 overexpressing 786-O cells transfected with siRNA-Control (si-Control) or siRNA-ATG7 (si-ATG7). *p < 0.05.

**Fig. 8:**
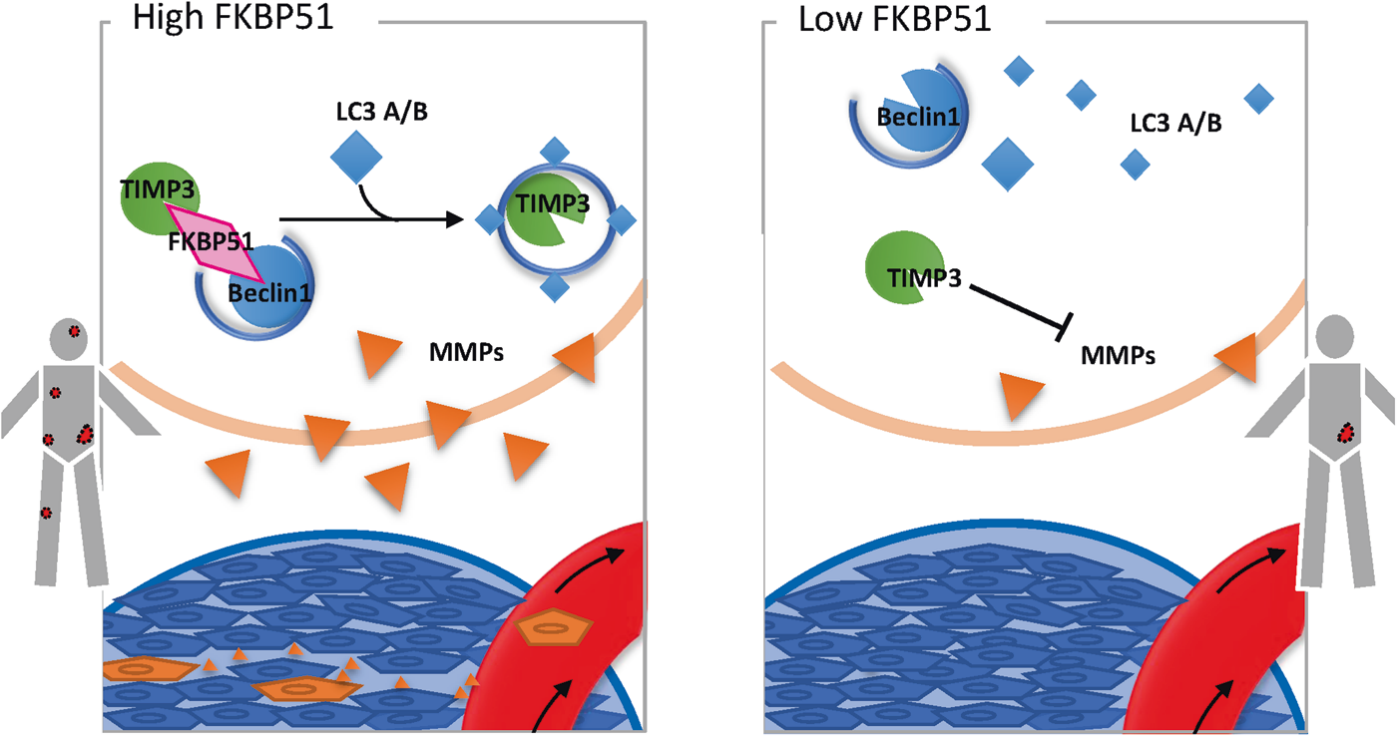
Schematic model of the relationship among FKBP51 and TIMP3/MMPs in ccRCC. FKBP51 promotes ccRCC invasion and migration by downregulating TIMP3 through connecting TIMP3 with Beclin1 complex and upregulating the expression and functions of downstream MMPs.

To determine whether FKBP51-induced invasion and migration are mediated through the autophagic degradation of TIMP3, 786-O, and Caki-1 cells stably overexpressing FKBP51 were transfected with the pcDNA3.1-TIMP3 plasmid to rescue TIMP3 expression. Transwell assays showed that pcDNA3.1-TIMP3 transfection reduced the FKBP51 overexpression-induced invasion and migration of 786-O and Caki-1 cells (Fig. 7a, b). The increased zymographic activity of MMP9 and the MMPs complex induced by FKBP51 overexpression could also be blocked by pcDNA3.1-TIMP3 transfection (Fig. 7c). At the protein level, pcDNA3.1-TIMP3 rescued the expression of TIMP3 and inhibited the expression of MMP7 and MMP9 (Fig. 7d and S5c). In addition, to further verify that autophagy works in FKBP51/TIMP3/MMP7,9 regulation, we transfected FKBP51 overexpressing 786-O cells with siRNA-ATG7 and found that siRNA-ATG7 rescued the inhibition of TIMP3 induced by FKBP51 overexpression and reduced the upregulation of MMP7 and MMP9 (Fig. 7e). The zymographic activity of MMP9 and the MMPs complex induced by FKBP51 overexpression was also blocked by siRNA-ATG7 (Fig. 7f). Taken together, the above data demonstrated that FKBP51 promotes invasion and migration in ccRCC cell lines by increasing the autophagic degradation of TIMP3 (Fig. 8).

## Discussion

In the present study, we reported that FKBP51 was upregulated in ccRCC tissues and cell lines. Overexpression of FKBP51 increased the invasive and migratory capacity of ccRCC cells while knockdown of FKBP51 suppressed this capacity. We mechanistically identified that FKBP51 could interact with TIMP3 and connect TIMP3 with the Beclin1 complex, thereby promoting the autophagic degradation of TIMP3 and the expression and functions of downstream MMP7 and MMP9.

The effects and mechanisms of FKBP51 in the regulation of proteolytic activity regulation and oncological behaviors of ccRCC have not been well characterized before. Previous studies have reported that FKBP51 overexpression induces AKT pathway inactivation and NF‐κB pathway activation, thereby enhancing the invasion and migration capacity of melanoma cells [31, 32]. Besides, FKBP51 promotes the invasion and migration of papillary thyroid carcinoma through NF-κB-dependent epithelial-to-mesenchymal transition [27]. However, our results showed no significant change in the levels of AKT phosphorylation and NF‐κB activity induced by FKBP51. The various genetic abnormalities of ccRCC may be responsible for this [33, 34]. In this study, the autophagic degradation of TIMP3 regulated by FKBP51 was identified as the crucial mechanism underlying ccRCC invasion and migration. Autophagy has recently been described as a regulator of tumor metastasis, and molecular mechanisms underlying autophagy-dependent invasion and migration are only beginning to emerge. Some studies have established a key role of autophagy in the control of focal adhesion (FA) turnover, the regulation of ECM components and Rho signaling during cell invasion and migration [35–40]. FKBP51 is thought to impact autophagy and Beclin1 mainly through two mechanisms: phosphorylation of Beclin1 itself and its regulatory E3 ligase S phase kinase-associated protein 2 (SKP2) [29, 41]. Both mechanisms involve the activity of AKT. The limited influences of FKBP51 expression on the phosphorylation of AKT and the expression of Belcin1 we observed support that FKBP51 promotes ccRCC invasion and migration through its “bridge” function connecting TIMP3 and autophagy. Thus, our finding about FKBP51 mediated autophagic degradation of TIMP3 provides not only novel insight into autophagy regulation in tumor metastasis but also an attempt to redefine the regulatory roles of FKBP51 in tumor cell autophagy.

As a key component of proteolytic activity regulation, TIMP3 was found to be associated with cancer progression and poor patient prognosis [42, 43]. Our study is not the first to report the regulations of TIMP3 in RCC. Masson et al. observed downregulated expression of TIMP3 mRNA and protein in 105 ccRCC clinical samples [44]. Chen et al. found that TIMPs are a direct target of miR-21 and that inhibition of TIMP3 promoted the invasion and migration abilities of RCC cells [45]. Li et al. found that circCSNK1G3 upregulated miR-181b, therefore inhibiting TIMP3 and enhancing the epithelial to mesenchymal transition (EMT) process, thus promoting the metastasis of RCC [46]. These observations are consistent with our finding that the degradation of TIMP3 induced by FKBP51 promotes the invasion and migration of ccRCC cell lines. Indeed, earlier studies mainly focused on the regulation of TIMP3 expression at the transcriptional level [47–49]. The mechanisms underlying the posttranscriptional regulation of TIMP3 have remained unclear, and few studies have linked the regulation of TIMP3 with autophagy. Consistent with our findings, reduced TIMP3 levels were reported to be accompanied by accumulation of Beclin1 and LC3 and increased autophagosomes under induction by decorin, a prototypical small leucine-rich proteoglycan (SLRP) that acts as a soluble tumor repressor that counteracts tumorigenic and angiogenic growth [50]. Another study using a rat model of hypertensive nephropathy observed that the inhibition of TIMP3 was accompanied by a decrease in autophagy markers [51]. Undoubtedly, our findings provide a new and valuable insight for further studies on the protein level regulation of TIMP3.

In conclusion, our study showed that FKBP51 acts as a promoter of ccRCC invasion and migration. The role of FKBP51 in the connection between TIMP3 and the Beclin1 autophagic complex is crucial to FKBP51-induced ccRCC invasion and migration. This finding reveals a novel function of FKBP51 and provides a new perspective on the mechanisms of invasion and migration of kidney cancer and other types of cancer. FKBP51 may be a biomarker for high metastatic capacity and a potential target for the prevention of metastasis in ccRCC. In addition, given the important roles that TIMP3/MMPs play in ECM function, we expect our study to provide a new direction for other physiopathological studies investigating autophagy related ECM regulation and remodeling.

## Methods and materials

### Bioinformatics

RNA-Seq expression data were downloaded from TCGA database (https://portal.gdc.cancer.gov/cart). The R package “limma” was applied to calculate the differentially expressed genes (DEGs) between the tumor and normal tissue, and between stage IV samples and stage I-III samples. Gene ontology (GO) enrichment and Kyoto Encyclopedia of Gene and Genome (KEGG) pathway enrichment of DEGs and Co-IP–MS-identified proteins were analyzed with the R package “clusterProfiler”. Additionally, the online Search Tool for the Retrieval of Interacting Genes (STRING; http://STRING-db.org) was applied to construct the protein–protein interaction (PPI) network to visualize the potential protein regulation axes. Cytoscape software with MCODE and cytoHubba plug-ins was used to analyze the functional modules and the significant nodes of the PPI network. Data and analysis results were visualized using the R package “ggplot2” and GraphPad Prism 8.0 software.

### Patients and tumor specimens

Matched tumors (*n* = 70) and ANTs (*n* = 49) were obtained from 70 ccRCC patients in the Minimally Invasive Urology Center, Shandong Provincial Hospital Affiliated to Shandong University. Samples were sent to Guge Ltd, Co. (Jinan, China) to combine into two tissue microarrays. Informed consent was obtained from the patients, and the collection of tissues was approved by the Ethics Committee of Shandong Provincial Hospital Affiliated to Shandong University (Ethical Review of Medical Research on Human Beings, No. 2017-209).

### Immunohistochemistry and immunofluorescence

IHC was carried out as previously described [26]. The primary antibodies were rabbit anti-FKBP51 (Abcam, ab126715, diluted: 1:200), rabbit anti-TIMP3 (Abcam, ab39184, diluted: 1:300), rabbit anti-MMP7 (Abcam, ab205525, diluted: 1:200), and rabbit anti-MMP9 (Abcam, ab39184, diluted: 1:200). Evaluation of TMC immunostaining was quantified by the H-score method.

Cells treated with siRNA-Control and siRNA-FKBP51 for 72 h were seeded on cover glasses at a concentration of 25,000 cells/ml. After being fixed with pre-cooled 95% alcohol, the cells were incubated with mouse anti-FKBP51 (Abcam, ab118050, diluted: 1:200), mouse anti-Beclin1 (Bioss, bsm-33323M, diluted: 1:100), and rabbit anti-TIMP3 (Abcam, ab39184, diluted: 1:200) overnight at 4 °C and then incubated with Alexa Fluor^®^ 555 (Abcam, ab150086, diluted: 1:1000), Alexa Fluor^®^ 647 (Abcam, ab150119, diluted: 1:1000) and DAPI for 1 h. Images were imaged using confocal microscopy (Olympus).

### Cell culture

The human renal proximal tubular epithelial (HK-2) and Caki-1 cell lines were purchased from the Cell Bank of Type Culture Collection of the Chinese Academy of Sciences. The 786-O, ACHN, and A498 cell lines were obtained from ATCC (Manassas, VA, USA). 786-O cells were routinely cultured in RPMI-1640, ACHN, and A498 in modified Eagle’s medium (MEM), Caki-1 in McCoy’s 5A medium, and normal renal HK-2 cells in Dulbecco’s modified Eagle’s medium (DMEM)/F12, supplemented with 10% (*v/v*) fetal bovine serum (FBS). 3-MA (HY-19312), MG132 (HY-13259), and rapamycin (HY-10219) were purchased from Med Chem Express Inc, New Jersey, USA. All media and FBS were purchased from Thermo Fisher Scientific Inc., Waltham, MA, USA. All cell lines were maintained in an incubator with a humidified atmosphere of 5% CO_2_ at 37 °C. All cells were used for experiments within 6 months.

### Transfection

786-O and Caki-1 cells were transfected with pcDNA3.1-FKBP51 packaging in the GV230 lentivirus vector constructed by Gene-Chem BioTECH (Shanghai, China) for FKBP51 overexpression; with pcDNA3.1-TIMP3 plasmid (Gene-Chem) for TIMP3 upregulation; with siRNA-FKBP51 (sc-35380) and siRNA-control (sc-37007) purchased from Santa Cruz Biotechnology for knockdown of FKBP51; and with siRNA-ATG7 (sc-41447) for knockdown of ATG7. All transfections were performed with Lipofectamine 2000 reagent (Invitrogen, 12566014) according to the manufacturer’s instructions.

### Western blotting

Total proteins were extracted with RIPA lysis and extraction buffer (Thermo Scientific, 89900) supplemented with a 1% Halt™ Protease and Phosphatase Inhibitor Cocktail (Thermo Scientific, 78442). A total of 20 µg of extracted total protein was used in blotting as described previously [21]. Primary antibodies for western blotting were as follows: anti-FKBP51 (Abcam, ab126715, diluted: 1:2000), anti-TIMP3 (Abcam, ab39184, diluted: 1:5000), anti-MMP7 (Abcam, ab205525, diluted: 1:1000), anti-MMP9 (Abcam, ab76003, diluted: 1:10000), anti-MMP21 (Santa Cruz, sc-398935, diluted: 1:2000), anti-Beclin1 (Abcam, ab207612, diluted: 1:2000), anti-LC3 A/B(CST, 12741, diluted: 1:2000), anti-ATG7(CST, 2631, diluted: 1:1000), anti-AKT Ser473 (Abcam, ab81283, diluted: 1:5000), anti-AKT Thr308 (Abcam, ab38449, diluted: 1:1000), anti-AKT 1/2/3 (Abcam, ab179463, diluted: 1:10,000), anti-IKBα (CST, 4812, diluted: 1:1000), and anti-GAPDH (Abcam, ab9485, diluted: 1:5000). HRP-conjugated goat anti-mouse (Santa Cruz, sc-2005, diluted: 1:10,000) or anti-rabbit (Santa Cruz, sc-2357, diluted: 1:10,000) secondary antibodies were used and then detected using an Immobilon Western Chemiluminescent HRP Substrate Kit (Millipore, WBKLS0100) on an Amersham Imager 600 (GE Healthcare). The gray values of western blotting strips were analyzed using ImageJ. GAPDH was used for normalization. All western blotting experiments were repeated at least three times and representative results are shown in the figures.

### Gelatin zymogram

MMPs activity in cell lines was measured by gelatin zymography. Cell lysates were loaded on 10% tris-glycine gel containing 0.1% gelatin and runed electrophoresis at 125 V constant. Gels were washed two times for 30 min at room temperature in 2.5% Triton X-100 to remove the SDS and then incubated with develop buffer (50 mM Tris-HCl, 10 mM CaCl2, 1% Triton X-100, pH 7.5) overnight at 37 °C, allowing gelatin degradation. After degradation, gels were stained for 30 min with 0.1% Coomassie blue in 45% methanol/10% acetic acid and destained in the same solution. Images were acquired with a digital gel imaging system (Tanon-5200Multi).

### RNA extraction, reverse transcription, and real-time quantitative PCR

Total RNA was isolated using TRIzol reagent (Invitrogen, 15596026), and then 1 mg total RNA was reverse transcribed to cDNA by using the PrimeScript RT Reagent Kit with gDNA Eraser (Takara, RR047A). RT-qPCR was performed using a Roche A700 sequence detection machine and SYBR GREEN (Roche, 04913914001) to quantify TIMP3 and GAPDH mRNA levels. GAPDH was used as an internal control for normalization. The following specific primer pairs were used: TIMP3: 5′-CTG CAA GGG CTG GGC ATC-3′(sense), 5′-TCC ATG GCC CGG TTG GCA GTG TGG AG-3′ (antisense).; GAPDH: 5′-GGT ATC GTG GAA GGA CTC-3′(sense), 5′-GTA GAG GCA GGG ATG ATG-3′ (antisense).

### Proliferation assays and flow cytometry

Cell Counting Kit-8 (CCK8) (Dojindo Molecular Technologies, Inc., Kumamoto, Japan) was used to evaluate cell proliferation by detecting changes in cell numbers. Cells were seeded on 96-well plates (786-O: 4 × 10^3^ cells per well; Caki-1: 5.5 × 10^3^ cells per well) and analyzed after 24, 48, and 72 h. A Muse cell-cycle kit (Merck Millipore) was applied to analyze the cell-cycle distribution in 786-O and Caki-1 cells fixed in 95% ice-cold ethanol overnight. After incubation with 200 μl of Muse cell-cycle reagent for 30 min in the dark at room temperature, the stained cells were detected using a Muse cell analyzer (Merck Millipore) to measure the distribution of cells in the G0/G1, S, and G2/M phases. All tests were repeated more than three times, and representative results are shown.

### Scratch, migration, and invasion assays

Confluent monolayer 786-O and Caki-1 cells in 6-well plates were scratched and photomicrographs were taken at 0 and 24 h using a Nikon fluorescence microscope, as previously described [52]. The areas of migration were measured by ImageJ software and the scratch healing ratio was calculated as follows: (area of 0 h – area of 24 h)/area of 0 h, representing the healing degree of scratches. Invasion and migration abilities were analyzed using a Boyden chamber containing 24-well transwell plates (Corning Inc. NY. USA). In brief, 7 × 10^4^ 786-O and Caki-1 cells in FBS free medium were seeded onto the 8-μm upper chamber or Matrigel-coated chamber. As a chemoattractant, medium containing 10% FBS was added to the lower chamber of the plate. After incubation at 37 °C in 5% CO_2_ for 24 h, cells on the top side of the membrane were removed with a cotton swab and the membranes were fixed in 95% pre-cooled ethanol. Migrated or invading cells were stained with 0.1% crystal violet and imaged. Three random microscopy fields were counted.

### Co-immunoprecipitation and mass spectroscopy

Co-IP experiments were conducted using a Pierce Co-Immunoprecipitation Kit (Thermo Scientific, 26149). Total protein was extracted from 786-O cells (~ 1 × 10^7^). Protein concentrations were determined by the Pierce BCA protein assay kit (Thermo Scientific, 23225). The sample was then split into two parts: 20 µg was used to determine the amounts of total input and 2 mg was used to perform Co-IP. For Co-IP, the lysate was precleaned using control agarose resin and incubated with FKBP51 antibody or control rabbit IgG immobilized beads at 4 °C overnight on a rotator. The immune complex was eluted and analyzed by western blotting or MS. Protein sample preparation for MS was conducted using a Pierce Mass Spec Sample Prep Kit for cultured cells following the manufacturer’s protocol (Thermo Scientific, 84840). The immune complex collected from Co-IP underwent reduction, alkylation, and acetone precipitation, and then digested with trypsin. The trypsinized peptides were analyzed using a QTRAP 5500 mass spectrometer.

### Statistical analyses

Statistical analyses were performed using GraphPad Prism 7.00 and SPSS version 22.0. The chi-square test was performed to assess the correlation between FKBP51 expression and clinical characteristics in patient samples. Overall survival curves were plotted according to the Kaplan–Meier method and verified by the Wilcoxon test. Paired or unpaired Student’s *t*-tests and one-way ANOVA were used to compare significance between two groups. The quantitative results of western blotting and Co-IP obtained by ImageJ software and their statistical results are shown in Supplementary Table 1. A two-tailed *p*-value <0.05 was considered significant.

## Supplementary information

supplementary materials in one

## Data Availability

The datasets used and/or analyzed during the current study are available from the corresponding author on reasonable request.
