# Supplementary material for: FKBP51 promotes invasion and migration by increasing the autophagic degradation of TIMP3 in clear cell renal cell carcinoma
Source: Cell Death Dis. 2021 Oct 1;12(10):899. doi: 10.1038/s41419-021-04192-8 (PMC8486832; doi:10.1038/s41419-021-04192-8)
Supplement: Supplementary file 1 — supplementary materials in one [file 41419_2021_4192_MOESM1_ESM.pdf]

Supplemental materials：

1，Supplemental figure 1-5 （Page 2 to 6）

2，Supplemental table 1 （Page 7 to 24）

3，TCGA samples id and information. (Page 25 to 41)

4，FKBP51 expression data in Oncomine beroukhim and yusenko cohorts. （Page 42）

Supplemental figures

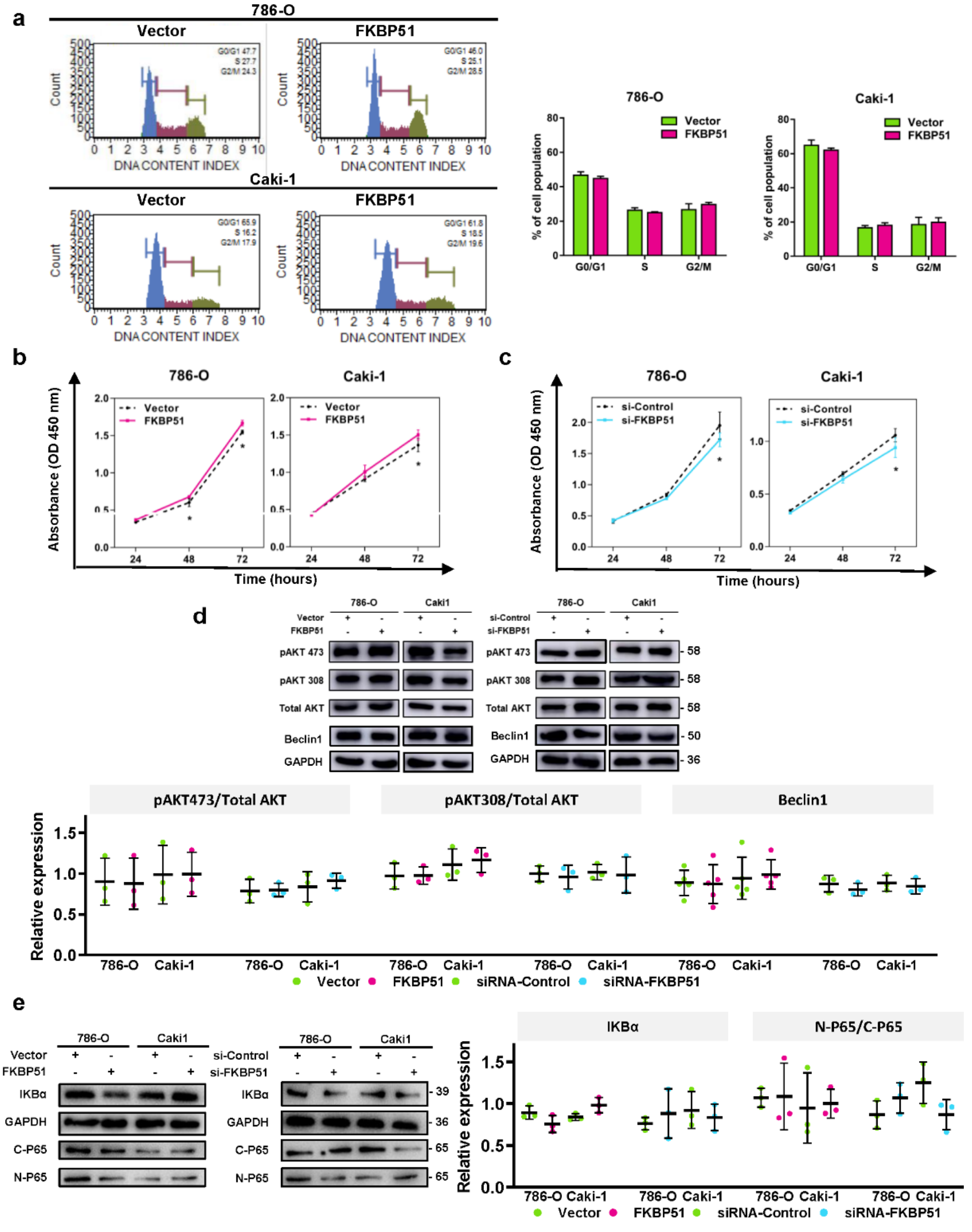

Fig. S1 a) Flow cytometric analysis of the cell cycle in 786-O and Caki-1 cells stably overexpressing FKBP51. Representative images and corresponding statistical results are presented by the histogram. **b)** The effect of FKBP51 overexpression on the proliferation of 786-O and Caki-1 cells was determined by CCK8 assay. **c)** The effect of FKBP51 knockdown on the proliferation of 786-O and Caki-1 cells was determined by CCK8 assay. **d)** Western blotting was conducted to determine the phosphorylation levels of AKT and the expression level of total AKT in FKBP51 overexpressing or knockdown 786-O and Caki-1 cells. **e)** Western blotting was conducted to determine

the expression level of IKBα and the levels of P65 in cytoplasm (C-P65) and nucleus (N-P65) in FKBP51 overexpressing or knockdown 786-O and Caki-1 cells. *, $p < 0.05$.

N	UNIPROTID	SYMBOL	N	UNIPROTID	SYMBOL	N	UNIPROTID	SYMBOL	N	UNIPROTID	SYMBOL	N	UNIPROTID	SYMBOL
**1**	P04264	KRT1	**53**	Q14315	FLNC	**105**	Q9H361	PABPC3	**157**	P38646	HSPA9	**209**	P0C0S5	H2AFZ
**2**	P35527	KRT9	**54**	O75369	FLNB	**106**	Q99988	GDF15	**158**	P40429	RPL13A	**210**	P62891	RPL39
**3**	P02538	KRT6A	**55**	Q9BTM1	H2AFJ	**107**	P52907	CAPZA1	**159**	Q8WUJ3	CEMIP	**211**	P62266	RPS23
**4**	P13645	KRT10	**56**	Q71UI9	H2AFV	**108**	P62244	RPS15A	**160**	O14950	MYL12A	**212**	P62280	RPS11
**5**	P02533	KRT14	**57**	P05386	RPLP1	**109**	O15143	ARPC1B	**161**	O14950	MYL12B	**213**	P61353	RPL27
**6**	P35908	KRT2	**58**	Q07020	RPL18	**110**	P54652	HSPA2	**162**	P04792	HSPB1	**214**	P62829	RPL23
**7**	P08779	KRT16	**59**	P06753	TPM3	**111**	Q9NYL9	TMOD3	**163**	Q07065	CKAP4	**215**	P23528	CFL1
**8**	Q5D862	FLG2	**60**	P17844	DDX5	**112**	P62906	RPL10A	**164**	P37108	SRP14	**216**	P63261	ACTG1
**9**	P48668	KRT6C	**61**	P18621	RPL17	**113**	Q96AG4	LRRC59	**165**	O15145	ARPC3	**217**	P62273	RPS29
**10**	P04259	KRT6B	**62**	P62899	RPL31	**114**	Q32P51	HNRNPA1L2	**166**	Q8WTX9	ZDHHC1	**218**	Q9H777	ELAC1
**11**	A0A0A0MT01	GSN	**63**	P56134	ATP5MF	**115**	P27658	COL8A1	**167**	Q14964	RAB39A	**219**	P42766	RPL35
**12**	A0A0A0MS51	GSN	**64**	Q6NZI2	CAVIN1	**116**	P46776	RPL27A	**168**	P61513	RPL37A	**220**	P62854	RPS26
**13**	A0A087WV22	RPL10	**65**	P23246	SFPQ	**117**	Q92743	HTRA1	**169**	P21291	CSRP1	**221**	P19474	TRIM21
**14**	A0A087WWU8	TPM3	**66**	P62913	RPL11	**118**	P14649	MYL6B	**170**	O15144	ARPC2	**222**	O95816	BAG2
**15**	A0A0A0MRF8	RPL17-C18orf32	**67**	P07951	TPM2	**119**	Q96L21	RPL10L	**171**	Q9UNX3	RPL26L1	**223**	P62910	RPL32
**16**	P35580	MYH10	**68**	P62136	PPP1CA	**120**	P62750	RPL23A	**172**	P35579	MYH9	**224**	Q9BQ48	MRPL34
**17**	O00159	MYO1C	**69**	P36873	PPP1CC	**121**	P50454	SERPINH1	**173**	P60709	ACTB	**225**	P62140	PPP1CB
**18**	P12814	ACTN1	**70**	Q03001	DST	**122**	Q9HAN9	NMNAT1	**174**	P08670	VIM	**226**	P63173	RPL38
**19**	Q71U36	TUBA1A	**71**	P61160	ACTR2	**123**	Q99878	HIST1H2AJ	**175**	P62701	RPS4X	**227**	P09382	LGALS1
**20**	P68363	TUBA1B	**72**	P23396	RPS3	**124**	Q96QV6	HIST1H2AA	**176**	P08708	RPS17	**228**	P61254	RPL26
**21**	Q9ULV4	CORO1C	**73**	P28799	GRN	**125**	Q96KK5	HIST1H2AH	**177**	P07437	TUBB	**229**	Q14764	MVP
**22**	P11940	PABPC1	**74**	P04406	GAPDH	**126**	Q93077	HIST1H2AC	**178**	P61158	ACTR3	**230**	P36578	RPL4
**23**	P47756	CAPZB	**75**	P81605	DCD	**127**	Q8IUE6	HIST2H2AB	**179**	P62269	RPS18	**231**	P62241	RPS8
**24**	P11142	HSPA8	**76**	P12259	F5	**128**	Q6FI13	HIST2H2AA3	**180**	P30050	RPL12	**232**	P46782	RPS5
**25**	Q9P0K7	RAI14	**77**	J3QRS3	MYL12A	**129**	Q6FI13	HIST2H2AA4	**181**	P19338	NCL	**233**	Q02878	RPL6
**26**	Q6WCQ1	MPRIP	**78**	Q5ST81	TUBB	**130**	Q16777	HIST2H2AC	**182**	P61247	RPS3A	**234**	P15880	RPS2
**27**	Q9UMS6	SYNPO2	**79**	J3QRN6	MYO1D	**131**	P20671	HIST1H2AD	**183**	P24844	MYL9	**235**	P18124	RPL7
**28**	O14763	TNFRSF10B	**80**	B4DXW1	ACTR3	**132**	P04908	HIST1H2AE	**184**	P62277	RPS13	**236**	P62081	RPS7
**29**	P47755	CAPZA2	**81**	F5H5D3	TUBA1C	**133**	P04908	HIST1H2AB	**185**	P68133	ACTA1	**237**	P62917	RPL8
**30**	P52272	HNRNPM	**82**	Q6ZN40	TPM1	**134**	P35625	**TIMP3**	**186**	P68032	ACTC1	**238**	P32969	RPL9
**31**	Q5QNW6	HIST2H2BF	**83**	M0R210	RPS16	**135**	Q99880	HIST1H2BL	**187**	P62424	RPL7A	**239**	P46781	RPS9
**32**	Q9NVI7	ATAD3A	**84**	Q6IPX4	RPS16	**136**	Q99879	HIST1H2BM	**188**	P62851	RPS25	**240**	P62753	RPS6
**33**	P68104	EEF1A1	**85**	B1AK85	CAPZB	**137**	Q99877	HIST1H2BN	**189**	P62249	RPS16	**241**	P46777	RPL5
**34**	P59998	ARPC4	**86**	B1AK88	CAPZB	**138**	Q93079	HIST1H2BH	**190**	P83731	RPL24	**242**	P39023	RPL3
**35**	Q69YQ0	SPECC1L	**87**	A8MUD9	RPL7	**139**	Q8N257	HIST3H2BB	**191**	P62888	RPL30	**243**	O75531	BANF1
**36**	Q92841	DDX17	**88**	X6R3G6	MFGE8	**140**	Q16778	HIST2H2BE	**192**	P12236	SLC25A6	**244**	P62805	HIST1H4I
**37**	Q9NR12	PDLIM7	**89**	F5GZN3	MFGE8	**141**	P62807	HIST1H2BD	**193**	P05141	SLC25A5	**245**	P62805	HIST1H4A
**38**	P07355	ANXA2	**90**	K7ELC2	RPS15	**142**	P62807	HIST1H2BG	**194**	P05387	RPLP2	**246**	P62805	HIST1H4D
**39**	P41221	WNT5A	**91**	Q60FE5	FLNA	**143**	P62807	HIST1H2BF	**195**	P39019	RPS19	**247**	P62805	HIST1H4F
**40**	P60660	MYL6	**92**	E7ETZ0	CALM1	**144**	P62807	HIST1H2BE	**196**	P61313	RPL15	**248**	P62805	HIST1H4K
**41**	P67936	TPM4	**93**	J3KTA4	DDX5	**145**	P62807	HIST1H2BI	**197**	P14923	JUP	**249**	P62805	HIST1H4J
**42**	Q15149	PLEC	**94**	G3V325	ATP5MF-PTCD1	**146**	P62807	HIST1H2BC	**198**	P27635	RPL10	**250**	P62805	HIST1H4C
**43**	P09493	TPM1	**95**	Q5TCU3	TPM2	**147**	P58876	HIST1H2BD	**199**	P46783	RPS10	**251**	P62805	HIST1H4H
**44**	Q9UEY8	ADD3	**96**	Q6P0N6	DST	**148**	P57053	LOC102724334	**200**	P62841	RPS15	**252**	P62805	HIST1H4B
**45**	P05388	RPLP0	**97**	F8W9J4	DST	**149**	P33778	HIST1H2BB	**201**	P50914	RPL14	**253**	P62805	HIST1H4E
**46**	P35611	ADD1	**98**	F6QMI7	DST	**150**	P23527	HIST1H2BO	**202**	Q7L7L0	HIST3H2A	**254**	P62805	HIST1H4L
**47**	P09651	HNRNPA1	**99**	E9PHM6	DST	**151**	P06899	HIST1H2BJ	**203**	P16104	H2AFX	**255**	P62805	HIST2H4A
**48**	P26373	RPL13	**100**	P83111	LACTB	**152**	O60814	HIST1H2BK	**204**	P0C0S8	HIST1H2AI	**256**	P62805	HIST4H4
**49**	P06396	GSN	**101**	P19105	MYL12A	**153**	Q96A08	HIST1H2BA	**205**	P0C0S8	HIST1H2AK	**257**	P62805	HIST2H4B
**50**	P62847	RPS24	**102**	O94832	MYO1D	**154**	Q05639	EEF1A2	**206**	P0C0S8	HIST1H2AL			
**51**	Q08431	MFGE8	**103**	P11021	HSPA5	**155**	P62979	RPS27A	**207**	P0C0S8	HIST1H2AM			
**52**	P21333	FLNA	**104**	Q9BQE3	TUBA1C	**156**	Q02543	RPL18A	**208**	P0C0S8	HIST1H2AG			

Fig. S2 The 257 proteins that interact with FKBP51 identified by Co-IP-coupled mass spectrometry (Co-IP–MS).

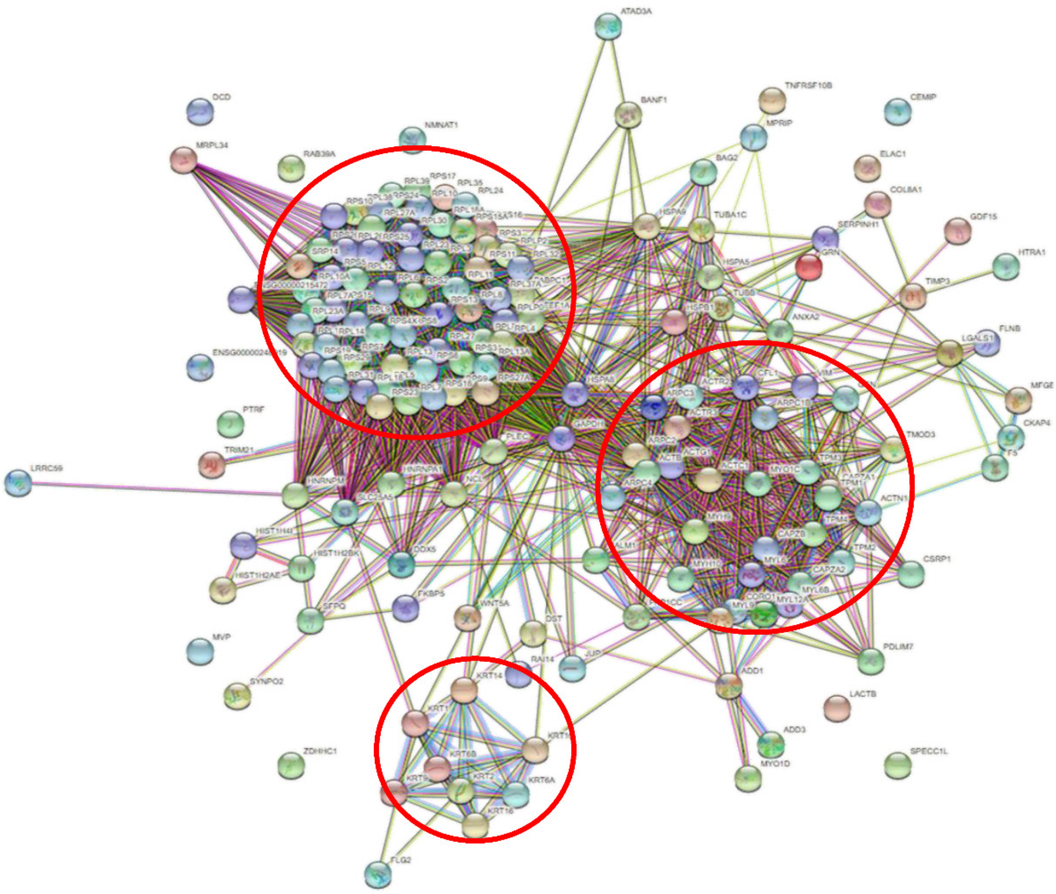

Fig. S3 The protein-protein interaction network of 257 proteins generated by STRING database.

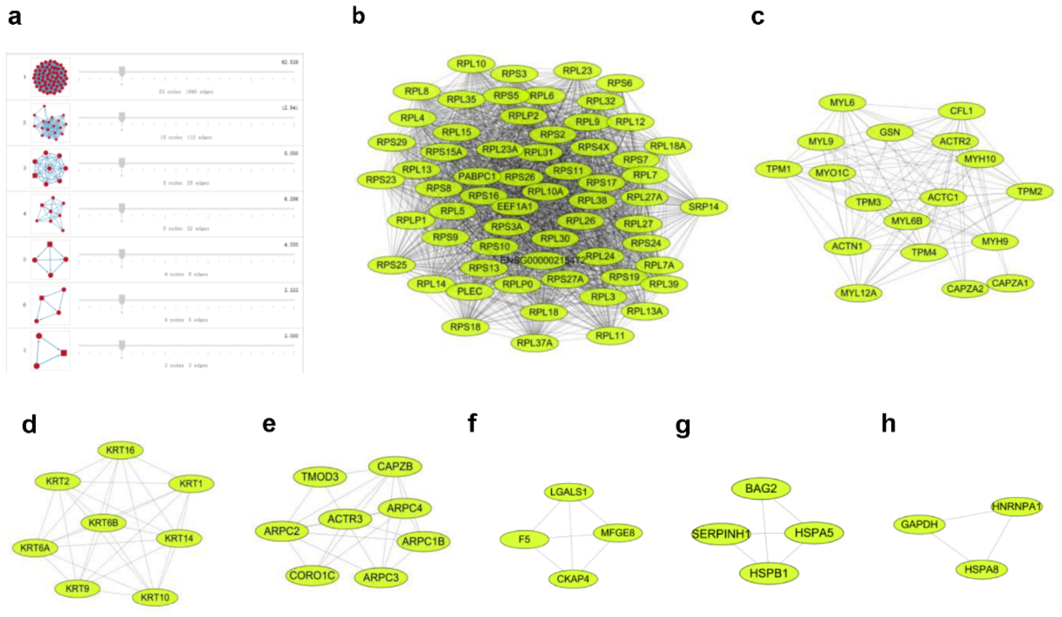

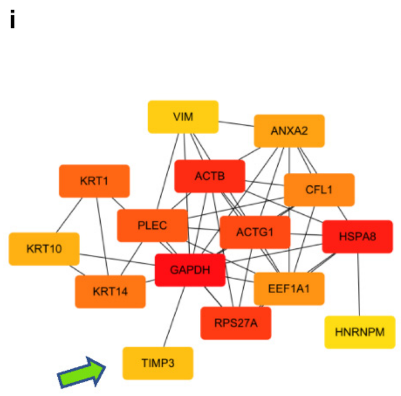

j

Top 15 in network string_interactions.tsv ranked by Betweenness method

Rank	Name	Score	
1	GAPDH	3343.71	Glyceraldehyde-3-phosphate dehydrogenase
2	HSPA8	1760.924	Heat shock cognate 71 kDa protein
3	ACTB	1593.291	Actin, cytoplasmic 1
4	RPS27A	1462.088	Ubiquitin-40S ribosomal protein S27a
5	ACTG1	1449.887	Actin, cytoplasmic 2
6	PLEC	1363.977	Plectin
7	KRT1	639.8728	Keratin, type I cytoskeletal
8	KRT14	631.8239	Keratin, type I cytoskeletal 14
9	CFL1	577.4396	Cofilin 1 (Non-muscle)
10	EEF1A1	499.3439	Putative elongation factor 1-alpha-like 3
11	ANXA2	486.6246	Annexin
12	KRT10	458.803	Keratin, type I cytoskeletal 10
**13**	**TIMP3**	**417.2186**	**Metalloproteinase inhibitor 3**
14	VIM	417.1494	Vimentin
15	HNRNPM	335.0046	Heterogeneous nuclear ribonucleoprotein M

Fig. S4 PPI network analysis on 257 proteins using cytoscape software. **a-h)** 7 modules calculated by MCODE plug-in. **i, j)** Top 15 significant nodes calculated by cytoHubba plug-in.

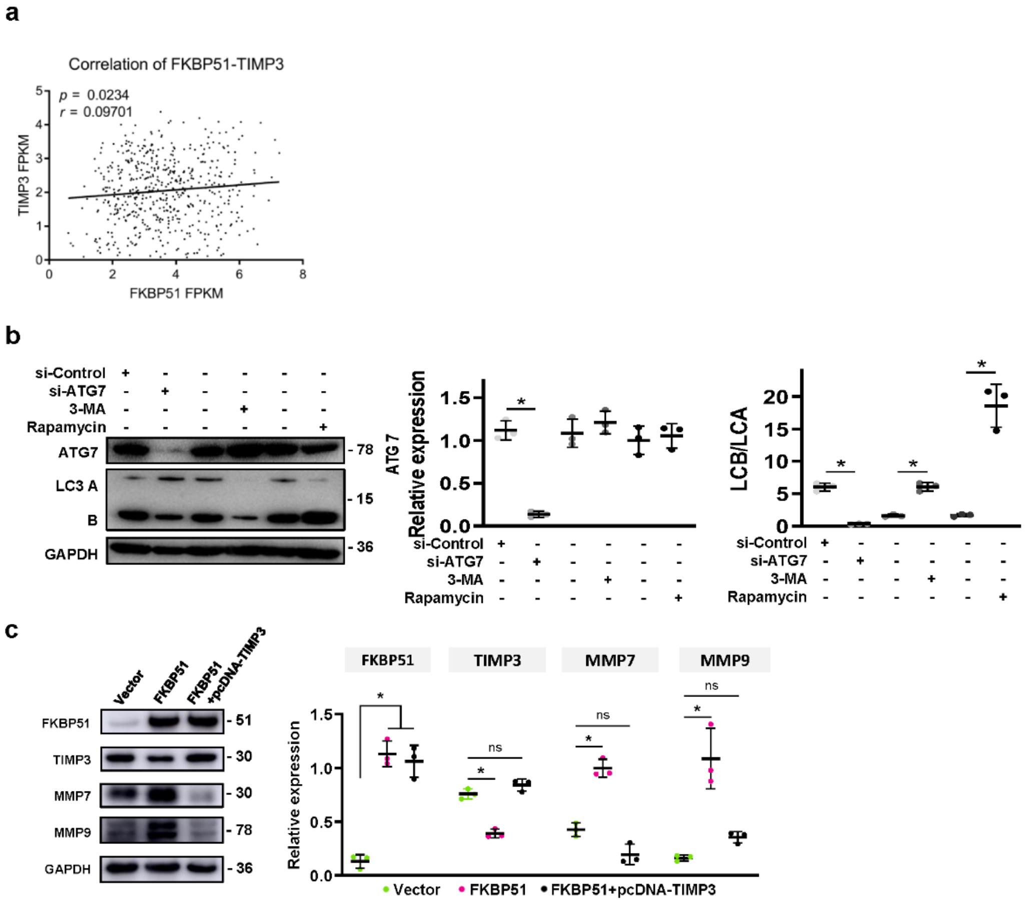

Fig. S5 a) Correlation analysis between FKBP51 and TIMP3 mRNA levels in TCGA cohort. **b)** Co-IP experiments in Caki-1 cells transfected with FKBP51 overexpress lentivirus (FKBP51) and empty vector lentivirus (Vector) using an anti-FKBP51 antibody with IgG as control. Schematic representation of quantitative data of indicated proteins. Representative images from three independent experiments are shown. **c)** Western blotting was conducted to determine the expression level of ATG7, LC3 A/B in 786-O cells transfected with siRNA-ATG7 (si-ATG7) or siRNA-Control (si-Control), treated with 3-MA (5 mM) or with rapamycin (1μM). **d)** Western blotting was used to detect the expression levels of TIMP3, MMP7 and MMP9 in FKBP51 overexpressing Caki-1 cells transfected with or without pcDNA3.1-TIMP3. *, $p < 0.05$.

Fig1g

	P1		P2		P3	
	ANT	T	ANT	T	ANT	T
FK	85.344	58.021	42.319	107.507	65.701	101.260
Gapdh	151.298	163.468	168.479	162.092	187.425	199.090
FKBP51-relative	0.564	0.355	0.251	0.663	0.351	0.509
FK	81.930	64.540	36.394	95.681	48.619	104.932
Gapdh	143.733	116.062	149.946	118.327	138.695	145.336
FKBP51-relative	0.570	0.556	0.243	0.809	0.351	0.722
FK	61.448	47.577	32.586	103.207	36.648	114.932
Gapdh	113.474	107.889	128.044	105.360	170.557	179.181
FKBP51-relative	0.542	0.441	0.254	0.980	0.215	0.641
FKBP51-relative	0.564	0.355	0.251	0.663	0.351	0.509
	0.570	0.556	0.243	0.809	0.351	0.722
	0.542	0.441	0.254	0.980	0.215	0.641
mean	0.559	0.451	0.249	0.817	0.305	0.624
std	0.012	0.082	0.005	0.129	0.064	0.088
ttest(ANT vs T)		0.141		0.003		0.014

	P4		P5		P6	
	ANT	T	ANT	T	ANT	T
FK	90.392	148.148	121.083	146.171	103.932	153.458
Gapdh	160.608	167.101	165.949	156.792	164.629	135.697
FKBP51-relative	0.563	0.887	0.730	0.932	0.631	1.131
FK	81.353	134.815	116.240	128.630	83.146	115.094
Gapdh	128.486	125.326	159.311	147.384	146.520	122.127
FKBP51-relative	0.633	1.076	0.730	0.873	0.567	0.942
FK	85.872	100.741	110.186	140.324	70.674	115.094
Gapdh	152.578	125.326	117.824	116.026	113.594	104.487
FKBP51-relative	0.563	0.804	0.935	1.209	0.622	1.102
FKBP51-relative	0.563	0.887	0.730	0.932	0.631	1.131
	0.633	1.076	0.730	0.873	0.567	0.942
	0.563	0.804	0.935	1.209	0.622	1.102
mean	0.586	0.922	0.798	1.005	0.607	1.058
std	0.033	0.114	0.097	0.147	0.028	0.083
ttest(ANT vs T)		0.016		0.172		0.002

	ANT	T
P1	0.559	0.451
P2	0.249	0.817
P3	0.305	0.624
P4	0.586	0.922
P5	0.798	1.005
P6	0.607	1.058
mean	0.664	0.995
std	0.095	0.056
ttest(ANT vs T)		0.043

Fig1h

	HK-2	ACHN	786-O	CAKI-1	CAKI-2
FK	88.77	256.17	216.57	149.49	151.68

Gapdh	198.89	198.67	198.72	187.23	168.29
FKBP51-relative	0.45	1.29	1.09	0.80	0.90
FK	63.91	245.81	202.91	150.62	148.65
Gapdh	173.04	198.67	129.16	153.53	148.09
FKBP51-relative	0.37	1.24	1.57	0.98	1.00
FK	77.23	236.05	210.01	159.12	136.79
Gapdh	147.18	156.95	129.16	172.25	126.21
FKBP51-relative	0.52	1.50	1.63	0.92	1.08
FKBP51-relative	0.446	1.289	1.090	0.798	0.901
	0.369	1.237	1.571	0.981	1.004
	0.525	1.504	1.626	0.924	1.084
mean	0.447	1.344	1.429	0.901	0.996
std	0.063	0.115	0.241	0.076	0.075
ttest(cancer vs HK-2)		0.005	0.030	0.017	0.008
			0.675	0.011	

Fig4a

	786-O		Caki-1	
OE	Vector	OE	Vector	OE
TIMP3	186.519	53.709	191.821	135.616
MMP7	151.685	222.574	29.647	184.27
MMP9	111.444	165.357	140.696	191.018
MMP21	185.328	189.958	179.141	188.936
GADPH	187.536	189.559	180.277	183.209
TIMP3-relative	0.99458	0.28334	1.06403	0.74023
MMP7-relative	0.80883	1.17417	0.16445	1.00579
MMP9-relative	0.59425	0.87232	0.78044	1.04262
MMP21-relative	0.98823	1.0021	0.9937	1.03126
TIMP3	167.867	39.7447	174.557	108.493
MMP7	148.651	202.542	20.4564	160.315
MMP9	95.8418	124.018	115.371	185.287
MMP21	179.768	150.067	157.644	160.042
GADPH	153.78	136.482	141.263	130.078
TIMP3-relative	1.09161	0.29121	1.23569	0.83406
MMP7-relative	0.96665	1.48402	0.14481	1.23245
MMP9-relative	0.62324	0.90867	0.81671	1.42443
MMP21-relative	1.169	1.09953	1.11596	1.23035
TIMP3	171.597	39.7447	138.111	128.835
MMP7	116.797	171.382	25.4964	160.315
MMP9	88.0408	107.482	120.999	157.982
MMP21	163.089	174.761	128.982	133.268
GADPH	136.901	141.977	140.616	142.903
TIMP3-relative	1.25344	0.27994	0.98219	0.90156
MMP7-relative	0.85315	1.20711	0.18132	1.12184
MMP9-relative	0.6431	0.75704	0.86049	1.10552
MMP21-relative	1.19129	1.23092	0.91726	0.93258
TIMP3-relative	0.99458	0.28334	1.06403	0.74023
TIMP3-relative	1.09161	0.29121	1.23569	0.83406
TIMP3-relative	1.25344	0.27994	0.98219	0.90156
mean	1.11321	0.28483	1.09397	0.82528
std	0.10678	0.00472	0.10563	0.06615
ttest(Vector vs OE/si)		0.00039		0.03808

MMP7-relative	0.80883	1.17417	0.16445	1.00579
MMP7-relative	0.96665	1.48402	0.14481	1.23245
MMP7-relative	0.85315	1.20711	0.18132	1.12184
mean	0.87621	1.28843	0.16353	1.12003
std	0.06646	0.13895	0.01492	0.09254
ttest(Vector vs OE/si)		0.01936		0.00013
MMP9-relative	0.59425	0.87232	0.78044	1.04262
MMP9-relative	0.62324	0.90867	0.81671	1.42443
MMP9-relative	0.6431	0.75704	0.86049	1.10552
mean	0.6202	0.84601	0.81921	1.19086
std	0.02006	0.06464	0.03273	0.16714
ttest(Vector vs OE/si)		0.00918		0.03672
MMP21-relative	0.98823	1.0021	0.9937	1.03126
MMP21-relative	1.169	1.09953	1.11596	1.23035
MMP21-relative	1.19129	1.23092	0.91726	0.93258
mean	1.11617	1.11085	1.00897	1.06473
std	0.09093	0.09375	0.08183	0.12385
ttest(Vector vs OE/si)		0.95683		0.62342

Fig4b

	786-O		Caki-1	
si	si-contro	si-FKBP5	si-contro	si-FKBP51
TIMP3	120.33	156.979	147.974	213.701
MMP7	225.389	55.551	219.656	136.042
MMP9	208.255	147.038	226.447	158.727
MMP21	177.68	173.74	180.341	208.428
GADPH	190.668	188.296	188.21	186.669
TIMP3-relative	0.6311	0.83368	0.78622	1.14481
MMP7-relative	1.1821	0.29502	1.16708	0.72879
MMP9-relative	1.09224	0.78089	1.20316	0.85031
MMP21-relative	0.93188	0.9227	0.95819	1.11656
TIMP3	82.2805	145.583	119.859	147.454
MMP7	214.12	36.1082	197.69	131.961
MMP9	147.861	108.808	167.571	150.791
MMP21	131.483	173.74	120.828	150.068
GADPH	125.841	134.53	150.625	143.735
TIMP3-relative	0.65385	1.08216	0.79575	1.02587
MMP7-relative	1.70151	0.2684	1.31247	0.91808
MMP9-relative	1.17498	0.8088	1.1125	1.04909
MMP21-relative	1.04484	1.29146	0.80218	1.04406
TIMP3	97.9234	140.874	107.258	147.454
MMP7	200.596	43.8853	195.494	110.194
MMP9	174.934	122.042	163.042	134.918
MMP21	156.358	151.154	180.341	206.344
GADPH	140.668	129.924	131.747	136.135
TIMP3-relative	0.69613	1.08428	0.81412	1.08314
MMP7-relative	1.42603	0.33778	1.48386	0.80944
MMP9-relative	1.2436	0.93933	1.23754	0.99106
MMP21-relative	1.11154	1.1634	1.36884	1.51572
TIMP3-relative	0.6311	0.83368	0.78622	1.14481
TIMP3-relative	0.65385	1.08216	0.79575	1.02587
TIMP3-relative	0.69613	1.08428	0.81412	1.08314
mean	0.66036	1.00004	0.79869	1.08461
std	0.02695	0.11764	0.01158	0.04857
ttest(Vector vs OE/si)		0.01639		0.00126

MMP7-relative	1.1821	0.29502	1.16708	0.72879
MMP7-relative	1.70151	0.2684	1.31247	0.91808
MMP7-relative	1.42603	0.33778	1.48386	0.80944
mean	1.43655	0.3004	1.32114	0.81877
std	0.21218	0.02858	0.12947	0.07756
ttest(Vector vs OE/si)		0.00169		0.00926
MMP9-relative	1.09224	0.78089	1.20316	0.85031
MMP9-relative	1.17498	0.8088	1.1125	1.04909
MMP9-relative	1.2436	0.93933	1.23754	0.99106
mean	1.17027	0.84301	1.1844	0.96349
std	0.06188	0.06906	0.05274	0.08346
ttest(Vector vs OE/si)		0.00754		0.03404
MMP21-relative	0.93188	0.9227	0.95819	1.11656
MMP21-relative	1.04484	1.29146	0.80218	1.04406
MMP21-relative	1.11154	1.1634	1.36884	1.51572
mean	1.02942	1.12585	1.04307	1.22545
std	0.07415	0.15287	0.239	0.20738
ttest(Vector vs OE/si)		0.46716		0.46076

Fig5a

	786		Caki1	
ip	IgG	FKIgG	IgG	FKIgG
TIMP3	3.948	228.766	3.112	203.972
FKBP51	10.756	222.711	1.54	126.601
input				
TIMP3	182.53	187.564	179.304	168.609
FKBP51	167.592	165.936	164.376	163.556
GAPDH	162.143	172.667	168.786	175.171
TIMP3-relative	1.12573	1.08628	1.06232	0.96254
FKBP51-relative	1.03361	0.96102	0.97387	0.93369
ip	IgG	FKIgG	IgG	FKIgG
TIMP3	3.5532	187.588	2.98752	181.535
FKBP51	9.46528	202.667	1.1396	96.2168
input				
TIMP3	129.596	133.17	143.443	141.632
FKBP51	117.314	119.474	161.088	150.845
GAPDH	129.714	134.68	151.907	143.64
TIMP3-relative	0.99909	0.98879	0.94428	0.98602
FKBP51-relative	0.90441	0.88709	1.06044	1.05016
ip	IgG	FKIgG	IgG	FKIgG
TIMP3	5.7506	183.177	5.04976	167.754
FKBP51	12.1106	187.689	3.3398	86.4089
input				
TIMP3	131.063	135.367	136.374	130.12
FKBP51	117.453	117.705	137.732	122.2
GAPDH	120.929	128.674	135.347	134.406
TIMP3-relative	1.08381	1.05202	1.00759	0.96812
FKBP51-relative	0.97126	0.91476	1.01763	0.90919
ip				
TIMP3	3.948	228.766	3.112	203.972
TIMP3	3.5532	187.588	2.98752	181.535
TIMP3	5.7506	183.177	5.04976	167.754
mean	4.41727	199.844	3.71643	184.42
std	0.95649	20.5303	0.94418	14.9262
ttest(Vector vs OE/si)		0.00018		6.9E-05

FKBP51	10.756	222.711	1.54	126.601
FKBP51	9.46528	202.667	1.1396	96.2168
FKBP51	12.1106	187.689	3.3398	86.4089
mean	10.7773	204.356	2.00647	103.076
std	1.08007	14.3474	0.95687	17.1101
ttest(Vector vs OE/si)		4.5E-05		0.00113
TIMP3-relative	1.12573	1.08628	1.06232	0.96254
TIMP3-relative	0.99909	0.98879	0.94428	0.98602
TIMP3-relative	1.08381	1.05202	1.00759	0.96812
mean	1.06954	1.04236	1.00473	0.97222
std	0.05268	0.04038	0.04823	0.01001
ttest(Vector vs OE/si)		0.59354		0.40355
FKBP51-relative	1.03361	0.96102	0.97387	0.93369
FKBP51-relative	0.90441	0.88709	1.06044	1.05
FKBP51-relative	0.97126	0.91476	1.01763	0.90919
mean	0.96976	0.92096	1.01731	0.96429
std	0.05276	0.0305	0.03534	0.06142
ttest(Vector vs OE/si)		0.32068		0.3497

Fig5b

	786		Caki1	
ip	IgG	T3IgG	IgG	T3IgG
TIMP3	5.206	148.258	3.917	137.045
FKBP51	9.399	121.833	7.189	92.577
input				
TIMP3	204.932	176.3	190.256	183.725
FKBP51	153.342	138.278	184.247	176.568
GAPDH	207.523	177.002	185.951	165.249
TIMP3-relative	0.98751	0.99603	1.02315	1.11181
FKBP51-relative	0.73892	0.78122	0.99084	1.0685
ip	IgG	T3IgG	IgG	T3IgG
TIMP3	10.6669	136.397	4.5253	123.341
FKBP51	7.61516	87.7198	6.20553	87.9482
input				
TIMP3	159.847	149.855	163.62	159.841
FKBP51	121.14	131.364	172.405	169.505
GAPDH	144.695	155.762	146.901	145.419
TIMP3-relative	1.10471	0.96208	1.11381	1.09917
FKBP51-relative	0.83721	0.84337	1.17361	1.16563
ip	IgG	T3IgG	IgG	T3IgG
TIMP3	9.93643	117.328	6.22115	105.193
FKBP51	10.5071	79.7764	8.69727	65.2626
input				
TIMP3	157.389	138.078	151.938	146.783
FKBP51	112.241	109.821	153.326	148.037
GAPDH	151.109	141.382	141.426	130.334
TIMP3-relative	1.04156	0.97663	1.07433	1.12621
FKBP51-relative	0.74278	0.77677	1.08414	1.13582
ip				
TIMP3	5.206	148.258	3.917	137.045
TIMP3	10.6669	136.397	4.5253	123.341
TIMP3	9.93643	117.328	6.22115	105.193
mean	8.6031	133.994	4.88782	121.859
std	2.42055	12.7411	0.97497	13.0457
ttest(Vector vs OE/si)		0.00017		0.00023

FKBP51	9.399	121.833	7.189	92.577
FKBP51	7.61516	87.7198	6.20553	87.9482
FKBP51	10.5071	79.7764	8.69727	65.2626
mean	9.17375	96.443	7.36393	81.9292
std	1.19132	18.2439	1.02474	11.9357
ttest(Vector vs OE/si)		0.00251		0.00092
TIMP3-relative	0.98751	0.99603	1.02315	1.11181
TIMP3-relative	1.10471	0.96208	1.11381	1.09917
TIMP3-relative	1.04156	0.97663	1.07433	1.12621
mean	1.0446	0.97825	1.07043	1.11239
std	0.04789	0.01391	0.03711	0.01104
ttest(Vector vs OE/si)		0.13306		0.20013
FKBP51-relative	0.73892	0.78122	0.99084	1.0685
FKBP51-relative	0.83721	0.84337	1.17361	1.16563
FKBP51-relative	0.74278	0.77677	1.08414	1.13582
mean	0.77297	0.80045	1.08286	1.12332
std	0.04545	0.0304	0.07462	0.04063
ttest(Vector vs OE/si)		0.51644		0.53761

Fig5e

V	0	1.5	3	6	9	12	15
TIMP3	96.4179	110.514	103.3	80.7154	68.1379	61.2575	52.8304
GAPDH	136.503	142.462	143.47	130.231	135.07	132.275	135.273
TIMP3-relative	0.70634	0.77575	0.72001	0.61979	0.50447	0.46311	0.39055
OE							
TIMP3	118.745	110.105	99.9493	75.6793	44.4402	14.1165	6.68225
GAPDH	167.849	177.278	170.682	176.942	200.071	181.665	188.036
TIMP3-relative	0.70745	0.62109	0.58559	0.42771	0.22212	0.07771	0.03554

Fig5f

V	0	1.5	3	6	9	12	15
TIMP3	149.486	148.152	136.209	119.864	122.375	113.904	112.516
GAPDH	146.59	135.07	146.854	140.635	143.485	138.967	149.859
TIMP3-relative	1.01975	1.09686	0.92751	0.85231	0.85288	0.81965	0.75081
OE							
TIMP3	146.361	143.977	125.419	118.66	101.449	81.762	74.6585
GAPDH	157.908	169.756	150.004	149.548	157.073	146.108	153.171
TIMP3-relative	0.92687	0.84814	0.8361	0.79346	0.64587	0.5596	0.48742

FigS5g

SCHX+MG132							
V	0	1.5	3	6	9	12	15
TIMP3	240.361	220.584	193.282	168.979	157.382	142.051	126.868
GAPDH	208.966	203.506	207.453	207.438	205.114	195.194	200.715
TIMP3-relative	1.15024	1.08392	0.93169	0.8146	0.76729	0.72774	0.63208
OE							
TIMP3	208.76	165.808	127.071	79.702	62.612	59.959	47.978
GAPDH	190.084	194.538	193.1	196.813	201.1	196.869	206.717
TIMP3-relative	1.09825	0.85232	0.65806	0.40496	0.31135	0.30456	0.2321

Fig5h

	786		
	OE	V	OE
ip	IgG	FKIgG	FKIgG
Beclin1	2.588	39.31	213.952
TIMP3	7.905	127.568	30.076
FKBP51	11.442	35.925	86.63
input			

Beclin1	148.245	123.51	139.114
TIMP3	121.035	200.235	139.535
FKBP51	194.655	48.65	187.605
GAPDH	186.555	180.045	173.43
Beclin1-relative	0.79465	0.686	0.80213
TIMP3-relative	0.64879	1.11214	0.80456
FKBP51-relative	1.04342	0.27021	1.08173

ip	IgG	FKIgG	FKIgG
Beclin1	2.681	55.692	164.384
TIMP3	15.306	187.748	43.104
FKBP51	7.989	41.75	118.13
input			
Beclin1	142.935	105.85	130.234
TIMP3	142.135	192.065	148.155
FKBP51	186.105	76.13	206.975
GAPDH	170.055	207.145	165.6
Beclin1-relative	0.84052	0.51099	0.78644
TIMP3-relative	0.83582	0.9272	0.89466
FKBP51-relative	1.09438	0.36752	1.24985

ip	IgG	FKIgG	FKIgG
Beclin1	1.919	70.104	135.408
TIMP3	13.617	141.423	35.344
FKBP51	8.877	33.005	127.4
input			
Beclin1	121.215	129.78	137.914
TIMP3	124.365	178.275	133.435
FKBP51	167.365	100.19	200.135
GAPDH	165.915	193.445	191.31
Beclin1-relative	0.73058	0.67089	0.72089
TIMP3-relative	0.74957	0.92158	0.69748
FKBP51-relative	1.00874	0.51792	1.04613

ip			
Beclin1	2.588	39.31	213.952
Beclin1	2.681	55.692	164.384
Beclin1	1.919	70.104	135.408
R	0.01387	0.21833	1.23365
R	0.01577	0.26886	0.99266
R	0.01157	0.3624	0.70779
mean	0.01373	0.2832	0.97803
std	0.00172	0.05968	0.21493
ttest（vs IgG； vs c）		0.00309	0.01164
TIMP3	7.905	127.568	30.076
TIMP3	15.306	187.748	43.104
TIMP3	13.617	141.423	35.344
	0.04237	0.70853	0.17342
	0.09001	0.90636	0.26029
	0.08207	0.73108	0.18475
mean	0.07148	0.78199	0.20615
std	0.02084	0.08842	0.03856
ttest（vs IgG； vs c）		0.00038	0.00108
FKBP51	11.442	35.925	86.63
FKBP51	7.989	41.75	118.13
FKBP51	8.877	33.005	127.4
	0.06133	0.19953	0.49951
	0.04698	0.20155	0.71335

	0.0535	0.17062	0.66593
mean	0.05394	0.19057	0.62626
std	0.00587	0.01413	0.09169
ttest（vs IgG； vs c)		0.00023	0.00267
Beclin1-relative	0.79465	0.686	0.80213
Beclin1-relative	0.70318	0.81881	0.83878
Beclin1-relative	0.75807	0.76635	0.97907
std	0.04573	0.3587	0.3858
ttest（vs IgG； vs c)		0.50721	0.90731
TIMP3-relative	0.64879	1.11214	0.80456
TIMP3-relative	0.63438	1.11026	0.78224
TIMP3-relative	0.757	1.06696	0.8618
mean	0.68006	1.09645	0.8162
std	0.05472	0.02087	0.03351
ttest（vs IgG； vs c)		0.00055	0.00055
FKBP51-relative	1.04342	0.27021	1.08173
FKBP51-relative	0.97628	0.37146	1.16039
FKBP51-relative	0.9954	0.42105	1.17153
mean	1.00503	0.35424	1.13788
std	0.02824	0.06277	0.03996
ttest（vs IgG； vs c)		0.00018	0.00012

Fig5i

	786		
	si-FK	si-C	si-FK
ip	IgG	BeclinIgG	Bclin1IgG
Beclin1	3.528	121.248	205.47
TIMP3	5.926	59.765	20.508
FKBP51	4.125	87.66	34.947
input			
Beclin1	126.54	159.782	140.121
TIMP3	132.426	78.49	148.208
FKBP51	19.152	151.488	16.188
GAPDH	129.504	136.684	138.892
Beclin1-relative	0.97711	1.16899	1.00885
TIMP3-relative	1.02256	0.57424	1.06707
FKBP51-relative	0.14789	1.10831	0.11655
ip	IgG	FKIgG	FKIgG
Beclin1	5.565	139.008	158.652
TIMP3	10.108	66.695	24.187
FKBP51	6.24	90.648	31.432
input			
Beclin1	115.32	168.502	160.871
TIMP3	124.466	97.01	130.258
FKBP51	5.052	138.508	44.648
GAPDH	134.964	128.544	122.082
Beclin1-relative	0.85445	1.31085	1.31773
TIMP3-relative	0.92222	0.75468	1.06697
FKBP51-relative	0.03743	1.07751	0.36572
ip	IgG	FKIgG	FKIgG
Beclin1	6.622	102.516	151.074
TIMP3	6.132	67.34	25.878
FKBP51	2.832	81.136	35.344
input			
Beclin1	137.08	178.402	151.381
TIMP3	117.266	85.87	114.248

FKBP51	15.382	161.248	28.698
GAPDH	122.114	136.744	107.972
Beclin1-relative	1.12256	1.30464	1.40204
TIMP3-relative	0.9603	0.62796	1.05813
FKBP51-relative	0.12596	1.1792	0.26579

ip			
Beclin1	3.528	121.248	205.47
Beclin1	5.565	139.008	158.652
Beclin1	6.622	102.516	151.074
	0.02724	0.88707	1.47935
	0.04123	1.0814	1.29955
	0.05423	0.74969	1.3992
mean	0.0409	0.90605	1.3927
std	0.01102	0.13608	0.07355
ttest（vs IgG； vs c)		0.00086	0.01125
TIMP3	5.926	59.765	20.508
TIMP3	10.108	66.695	24.187
TIMP3	6.132	67.34	25.878
	0.04576	0.43725	0.14765
	0.07489	0.51885	0.19812
	0.05022	0.49245	0.23967
mean	0.05696	0.48285	0.19515
std	0.01281	0.034	0.03763
ttest（vs IgG； vs c)		7.8E-05	0.00131
FKBP51	4.125	87.66	34.947
FKBP51	6.24	90.648	31.432
FKBP51	2.832	81.136	35.344
	0.03185	0.64133	0.25161
	0.04623	0.70519	0.25747
	0.02319	0.59334	0.32734
mean	0.03376	0.64662	0.27881
std	0.0095	0.04581	0.0344
ttest（vs IgG； vs c)		5E-05	0.00082
Beclin1-relative	0.97711	1.16899	1.00885
Beclin1-relative	0.85445	1.31085	1.31773
Beclin1-relative	1.12256	1.30464	1.40204
std	0.06133	0.58734	0.56232
ttest（vs IgG； vs c)		0.87895	0.9338
TIMP3-relative	1.02256	0.57424	1.06707
TIMP3-relative	0.92222	0.75468	1.06697
TIMP3-relative	0.9603	0.62796	1.05813
mean	0.96836	0.6523	1.06406
std	0.04136	0.07565	0.00419
ttest（vs IgG； vs c)		0.00659	0.00154
FKBP51-relative	0.14789	1.10831	0.11655
FKBP51-relative	0.03743	1.07751	0.36572
FKBP51-relative	0.12596	1.1792	0.26579
mean	0.10376	1.12167	0.24935
std	0.04775	0.04257	0.10239
ttest（vs IgG； vs c)		2.3E-05	0.00037

Fig6a

	V	V	OE	OE
	-	Rapa	-	Rapa
FKBP51	32.757	29.129	215.364	160.24
TIMP3	192.492	156.813	67.815	10.19
LC3A	57.181	11.495	10.082	7.67

LC3B	130.877	223.257	139.787	201.055
GAPDH	182.643	194.811	190.737	180.553
FKBP51-relative	0.17935	0.14952	1.12911	0.8875
TIMP3-relative	1.05392	0.80495	0.35554	0.05644
LC3A-relative	0.31308	0.05901	0.05286	0.04248
LC3B-relative	0.71657	1.14602	0.73288	1.11355
FKBP51	28.8262	29.6464	172.291	120.18
TIMP3	188.642	116.042	51.5394	7.8463
LC3A	46.8884	7.8166	22.9512	6.8263
LC3B	121.716	147.35	133.019	139.096
GAPDH	165.156	169.486	183.108	160.692
FKBP51-relative	0.17454	0.17492	0.94093	0.74789
TIMP3-relative	1.14221	0.68467	0.28147	0.04883
LC3A-relative	0.2839	0.04612	0.12534	0.04248
LC3B-relative	0.73697	0.86939	0.72645	0.86561
FKBP51	23.9126	25.4051	174.445	121.782
TIMP3	169.393	112.905	66.4587	7.2349
LC3A	48.6039	10.2306	13.9423	5.8292
LC3B	96.849	160.745	123.84	156.686
GAPDH	141.503	152.863	147.331	140.553
FKBP51-relative	0.16899	0.1662	1.18404	0.86645
TIMP3-relative	1.1971	0.73861	0.45109	0.05147
LC3A-relative	0.34348	0.06693	0.09463	0.04147
LC3B-relative	0.68443	1.05156	0.84056	1.11478
FKBP51-relative	0.17935	0.14952	1.12911	0.8875
FKBP51-relative	0.17454	0.17492	0.94093	0.74789
FKBP51-relative	0.16899	0.1662	1.18404	0.86645
	0.17429	0.16355	1.08469	0.83395
	0.00423	0.01054	0.1041	0.06145
vs CON+PBS		0.25174	0.00025	0.00011
VS OE				0.04267
TIMP3-relative	1.05392	0.80495	0.35554	0.05644
TIMP3-relative	1.14221	0.68467	0.28147	0.04883
TIMP3-relative	1.1971	0.73861	0.45109	0.05147
	1.16965	0.71164	0.36628	0.05015
	0.02745	0.02697	0.08481	0.00132
vs CON+PBS		0.00202	0.00028	1.3E-05
VS OE				0.00321
LC3A-relative	0.31308	0.05901	0.05286	0.04248
LC3A-relative	0.2839	0.04612	0.12534	0.04248
LC3A-relative	0.34348	0.06693	0.09463	0.04147
LC3B-relative	0.71657	1.14602	0.73288	1.11355
LC3B-relative	0.73697	0.86939	0.72645	0.86561
LC3B-relative	0.68443	1.05156	0.84056	1.11478
LCB/A	2.28882	19.4221	13.865	26.2132
LCB/A	2.59586	18.8509	5.79573	20.3765
LCB/A	1.99262	15.7123	8.88233	26.8795
	2.29243	17.9951	9.51436	24.4897
	0.24628	1.63095	3.32444	2.92116
vs CON+PBS		0.00018	0.03752	0.00043
VS OE			0.03171	0.00874

Fig6b

	C	C	siFK	siFK
	-	Rapa	-	Rapa

FKBP51	223.959	182.099	37.101	15.016
TIMP3	137.17	68.632	192.71	173.625
LC3A	84.167	12.733	81.11	19.347
LC3B	161.441	212.206	107.661	211.987
GAPDH	202.092	192.882	175.415	157.908
FKBP51-relative	1.1082	0.9441	0.2115	0.09509
TIMP3-relative	0.67875	0.35582	1.09859	1.09953
LC3A-relative	0.41648	0.06601	0.46239	0.12252
LC3B-relative	0.79885	1.10019	0.61375	1.34247
FKBP51	201.563	174.815	28.9388	13.6646
TIMP3	98.7624	68.632	144.533	130.219
LC3A	73.2253	9.54975	57.5881	11.4233
LC3B	121.081	178.253	101.201	182.631
GAPDH	145.506	152.882	163.136	146.852
FKBP51-relative	1.38525	1.14346	0.17739	0.09305
TIMP3-relative	0.67875	0.44892	0.88596	0.88674
LC3A-relative	0.50325	0.06246	0.35301	0.07779
LC3B-relative	0.83213	1.16595	0.62035	1.24364
FKBP51	172.448	158.426	31.1648	13.3642
TIMP3	108.364	68.632	132.97	171.889
LC3A	74.067	8.40378	72.1879	15.2384
LC3B	150.14	148.544	95.8183	201.388
GAPDH	143.485	159.736	150.857	142.117
FKBP51-relative	1.20185	0.9918	0.20659	0.09404
TIMP3-relative	0.75523	0.42966	0.88143	1.20949
LC3A-relative	0.5162	0.05261	0.47852	0.10722
LC3B-relative	1.04638	0.92993	0.63516	1.41705
FKBP51-relative	1.1082	0.9441	0.2115	0.09509
FKBP51-relative	1.38525	1.14346	0.17739	0.09305
FKBP51-relative	1.20185	0.9918	0.20659	0.09404
	1.23177	1.02645	0.19849	0.09406
	0.11507	0.085	0.01506	0.00083
vs CON+PBS		0.11225	0.00023	0.00015
VS OE				0.00061
TIMP3-relative	0.67875	0.35582	1.09859	1.09953
TIMP3-relative	0.67875	0.44892	0.88596	0.88674
TIMP3-relative	0.75523	0.42966	0.88143	1.20949
	0.71699	0.43929	0.8837	1.04811
	0.03824	0.00963	0.00227	0.16138
vs CON+PBS		0.00155	0.02988	0.02121
VS OE				0.4071
LC3A-relative	0.41648	0.06601	0.46239	0.12252
LC3A-relative	0.50325	0.06246	0.35301	0.07779
LC3A-relative	0.5162	0.05261	0.47852	0.10722
LC3B-relative	0.79885	1.10019	0.61375	1.34247
LC3B-relative	0.83213	1.16595	0.62035	1.24364
LC3B-relative	1.04638	0.92993	0.63516	1.41705
LCB/A	1.9181	16.6658	1.32735	10.9571
LCB/A	1.65354	18.6657	1.75733	15.9875
LCB/A	2.02709	17.6759	1.32735	13.2158
	1.86624	17.6691	1.47067	13.3868
	0.15685	0.81647	0.2027	2.05723
vs CON+PBS		1.1E-05	0.09446	0.05211
VS OE				0.00123

Fig6c

gCHX		0	3	6	9	12	15
V	TIMP3	225.028	228.597	216.032	196.399	159.198	113.984
	GAPDH	211.972	209.867	201.25	214.528	210.283	210.994
	TIMP3-re	1.06159	1.08925	1.07345	0.91549	0.75707	0.54022
OE	TIMP3	229.276	199.293	157.449	93.298	43.676	12.147
	GAPDH	219.844	211.068	206.201	171.766	209.974	203.893
	TIMP3-re	1.0429	0.94421	0.76357	0.54317	0.20801	0.05958

Fig6d

gCHX+siATG7		0	3	6	9	12	15
V	TIMP3	220.891	212.996	190.109	185.367	173.887	144.359
	GAPDH	217.272	202.429	200.783	208.842	188.69	205.516
	TIMP3-re	1.01666	1.0522	0.94684	0.88759	0.92155	0.70242
OE	TIMP3	208.295	231.858	229.429	160.015	134.978	146.593
	GAPDH	201.367	234.09	230.527	222.91	206.607	199.15
	TIMP3-re	1.0344	0.99047	0.99524	0.71785	0.65331	0.73609

Fig6e

gCHX		0	3	6	9	12	15
si-Control	TIMP3	223.954	208.468	198.237	147.877	85.6	59.037
	GAPDH	196.724	207.802	178.125	170.786	165.599	160.771
	TIMP3-re	1.13842	1.0032	1.11291	0.86586	0.51691	0.36721
siFKBP51	TIMP3	215.493	225.594	202.804	190.266	154.371	108.08
	GAPDH	187.174	177.061	174.697	171.343	176.81	187.692
	TIMP3-re	1.1513	1.2741	1.16089	1.11044	0.87309	0.57584

Fig6f

gCHX+Rapa		0	3	6	9	12	15
si-Control	TIMP3	223.924	216.323	141.399	52.302	32.892	13.323
	GAPDH	177.864	180.707	184.755	200.184	189.748	155.721
	TIMP3-re	1.25896	1.19709	0.76533	0.26127	0.17335	0.08556
siFKBP51	TIMP3	202.343	206.529	202.744	176.51	113.849	82.173
	GAPDH	184.802	178.492	174.794	170.603	179.262	202.778
	TIMP3-re	1.09492	1.15708	1.1599	1.03462	0.6351	0.40524

Fig7d

	786-0		
	Vector	OE	OE+TIMP3
FKBP51	78.711	190.871	206.939
TIMP3	178.697	140.266	191.145
MMP7	97.376	204.295	148.965
MMP9	128.548	225.948	163.242
GAPDH	195.91	197.213	208.307
FKBP51-relative	0.40177	0.96784	0.99343
TIMP3-relative	0.91214	0.71124	0.91761
MMP7-relative	0.49704	1.03591	0.71512
MMP9-relative	0.65616	1.14571	0.78366
FKBP51	69.2657	162.24	159.343
TIMP3	130.449	99.7474	181.588
MMP7	73.032	169.565	103.006
MMP9	94.2642	183.018	122.432
GAPDH	162.605	169.603	164.563
FKBP51-relative	0.42597	0.95659	0.96828
TIMP3-relative	0.80224	0.58812	1.10346
MMP7-relative	0.44914	0.99977	0.62594
MMP9-relative	0.57971	1.07909	0.74398

FKBP51	70.0528	137.427	151.065
TIMP3	137.597	81.1968	177.765
MMP7	72.0582	202.252	137.048
MMP9	105.696	162.683	120.799
GAPDH	172.401	161.715	183.726
FKBP51-relative	0.40634	0.84981	0.82223
TIMP3-relative	0.79812	0.5021	0.96756
MMP7-relative	0.41797	1.25067	0.74594
MMP9-relative	0.61309	1.00599	0.6575
FKBP51-relative	0.40177	0.96784	0.99343
FKBP51-relative	0.42597	0.95659	0.96828
FKBP51-relative	0.40634	0.84981	0.82223
mean	0.41136	0.92475	0.92798
std	0.0105	0.05319	0.07548
ttest vs control		0.00018	0.00066
ttest vs OE			0.96285
TIMP3-relative	0.91214	0.71124	0.91761
TIMP3-relative	0.80224	0.58812	1.10346
TIMP3-relative	0.79812	0.5021	0.96756
mean	0.8375	0.60049	0.99621
std	0.0528	0.08583	0.07853
ttest vs control		0.02921	0.07667
ttest vs OE			0.00858
MMP7-relative	0.49704	1.03591	0.71512
MMP7-relative	0.44914	0.99977	0.62594
MMP7-relative	0.41797	1.25067	0.74594
mean	0.45472	1.09545	0.69567
std	0.03252	0.11074	0.05088
ttest vs control		0.00142	0.00486
ttest vs OE			0.00974
MMP9-relative	0.65616	1.14571	0.78366
MMP9-relative	0.57971	1.07909	0.74398
MMP9-relative	0.61309	1.00599	0.6575
mean	0.61632	1.07693	0.72838
std	0.03129	0.05706	0.05267
ttest vs control		0.00056	0.0609
ttest vs OE			0.00316

Fig7e

F	Vector	FKOE	FK+siC	FK+siATG7
FKBP51	36.545	193.067	201.262	179.32
ATG7	180.664	152.332	149.109	68.527
TIMP3	206.071	35.136	49.829	201.264
MMP7	148.485	188.603	208.103	94.083
MMP9	121.964	163.103	188.155	111.433
GAPDH	230.982	228.376	216.333	214.874
FKBP51-relative	0.15822	0.84539	0.93033	0.83454
ATG7-relative	0.78216	0.66702	0.68926	0.31892
TIMP3-relative	0.89215	0.15385	0.23033	0.93666
MMP7-relative	0.64284	0.82584	0.96196	0.43785
MMP9-relative	0.52802	0.71419	0.86975	0.5186
FKBP51	26.8905	171.83	142.896	145.249

ATG7	137.305	112.726	120.778	54.6247
TIMP3	179.282	56.1088	54.4478	158.999
MMP7	114.333	179.173	181.915	85.5623
MMP9	109.768	158.21	160.747	95.8324
GAPDH	163.997	178.133	212.006	178.345
FKBP51-relative	0.16397	0.96461	0.67402	0.81443
ATG7-relative	0.83724	0.63282	0.56969	0.30629
TIMP3-relative	1.0932	0.31498	0.25682	0.89152
MMP7-relative	0.69717	1.00584	0.85806	0.47976
MMP9-relative	0.66933	0.88815	0.75822	0.53734
FKBP51	31.4433	148.662	158.997	163.181
ATG7	126.465	112.726	123.76	57.9038
TIMP3	166.918	38.0115	45.8461	154.973
MMP7	107	180.313	193.536	88.3789
MMP9	95.8658	166.793	171.858	101.404
GAPDH	207.884	212.39	166.576	180.494
FKBP51-relative	0.15125	0.69995	0.9545	0.90408
ATG7-relative	0.60834	0.53075	0.74297	0.32081
TIMP3-relative	0.80294	0.17897	0.27523	0.85861
MMP7-relative	0.51471	0.84897	1.16184	0.48965
MMP9-relative	0.46115	0.78531	1.03171	0.56181
FKBP51-relative	0.15822	0.84539	0.93033	0.83454
FKBP51-relative	0.16397	0.96461	0.67402	0.81443
FKBP51-relative	0.15125	0.69995	0.9545	0.90408
mean	0.15781	0.83665	0.85295	0.85101
std	0.0052	0.10823	0.12691	0.03841
ttest vs control		0.0009		1.5E-05
ttest vs OE				1.5E-05
ATG7-relative	0.78216	0.66702	0.68926	0.31892
ATG7-relative	0.83724	0.63282	0.56969	0.30629
ATG7-relative	0.60834	0.53075	0.74297	0.32081
mean	0.74258	0.6102	0.6673	0.31534
std	0.09755	0.05789	0.07242	0.00645
ttest vs control		0.17418		0.00348
ttest vs OE				0.00201
TIMP3-relative	0.89215	0.15385	0.23033	0.93666
TIMP3-relative	1.0932	0.31498	0.25682	0.89152
TIMP3-relative	0.80294	0.17897	0.27523	0.85861
mean	0.92943	0.21593	0.25413	0.8956
std	0.1214	0.07078	0.01843	0.032
ttest vs control		0.00199		0.72248
ttest vs OE				0.00025
MMP7-relative	0.64284	0.82584	0.96196	0.43785
MMP7-relative	0.69717	1.00584	0.85806	0.47976
MMP7-relative	0.51471	0.84897	1.16184	0.48965
mean	0.61824	0.89355	0.99396	0.46909
std	0.07649	0.07996	0.12606	0.02245
ttest vs control		0.02448		0.05722
ttest vs OE				0.00194
MMP9-relative	0.52802	0.71419	0.86975	0.5186
MMP9-relative	0.66933	0.88815	0.75822	0.53734
MMP9-relative	0.46115	0.78531	1.03171	0.56181

mean	0.55283	0.79589	0.88656	0.53925
std	0.08678	0.07141	0.11228	0.01769
ttest vs control		0.03772		0.8389
ttest vs OE				0.00786

FigS1d

	786-O		Caki-1		786-O		Caki-1	
	Vector	OE	Vector	OE	si-Contrc	si-FKBP5	si-Contrc	si-FKBP5
pAkt 473	222.095	235.109	236.016	194.555	173.22	198.004	182.495	188.657
pAkt 308	206.667	210.653	226.842	192.57	184.816	226.017	207.961	241.001
总AKT	182.3	192.253	170.389	151.042	183.717	222.027	184.714	198.237
pAkt 473/pan AKT	1.21829	1.22291	1.38516	1.28809	0.94286	0.8918	0.98799	0.95167
pAkt 308/pan AKT	1.13366	1.09571	1.33132	1.27494	1.00598	1.01797	1.12585	1.21572
pAkt 473	179.028	169.66	131.724	142.83	145.71	160.776	139.356	160.884
pAkt 308	207.648	192.354	195.102	186.21	211.464	231.462	208.332	189.892
总AKT	218.502	214.218	191.596	187.366	193.932	217.54	220.86	198.526
pAkt 473/pan AKT	0.81934	0.792	0.68751	0.7623	0.75135	0.73906	0.63097	0.81039
pAkt 308/pan AKT	0.95033	0.89794	1.0183	0.99383	1.0904	1.064	0.94328	0.95651
pAkt 473	147.06	130.88	164.448	156.12	135.656	148.536	155.052	183.852
pAkt 308	183.186	198.496	181.332	207.124	185.145	153.63	171.09	145.458
总AKT	222.552	212.976	184.644	168.786	206.082	194.112	174.168	187.56
pAkt 473/pan AKT	0.66079	0.61453	0.89062	0.92496	0.65826	0.76521	0.89024	0.98023
pAkt 308/pan AKT	0.82312	0.93201	0.98206	1.22714	0.8984	0.79145	0.98233	0.77553
pAkt 473/pan AKT	1.21829	1.22291	1.38516	1.28809	0.94286	0.8918	0.98799	0.95167
	0.81934	0.792	0.68751	0.7623	0.75135	0.73906	0.63097	0.81039
	0.66079	0.61453	0.89062	0.92496	0.65826	0.76521	0.89024	0.98023
mean	0.89948	0.87648	0.98776	0.99178	0.78416	0.79869	0.8364	0.9141
std	0.23455	0.25546	0.29298	0.21979	0.11848	0.0667	0.15064	0.07425
ttest vs control		0.930		0.988		0.887		0.549
pAkt 308/pan AKT	1.13366	1.09571	1.33132	1.27494	1.00598	1.01797	1.12585	1.21572
	0.95033	0.89794	1.0183	0.99383	1.0904	1.064	0.94328	0.95651
	0.82312	0.93201	0.98206	1.22714	0.8984	0.79145	0.98233	0.77553
mean	0.96904	0.97522	1.11056	1.1653	0.99826	0.95781	1.01715	0.98259
std	0.12747	0.08633	0.1568	0.12281	0.07857	0.11912	0.0785	0.18065
ttest vs control		0.957		0.717		0.709		0.816

	786-O		Caki-1		786-O		Caki-1	
	Vector	OE	Vector	OE	si-Contrc	si-FKBP5	si-Contrc	si-FKBP5
Beclin1	146.655	129.78	162.99	139.885	158.3	116.865	145.305	136.24
GAPDH	133.65	133.65	133.65	133.65	133.65	133.65	133.65	133.65
Beclin1/GAPDH	1.09731	1.22291	1.38516	1.28809	0.94286	0.8918	0.98799	0.95167
Beclin1	126.375	159.18	129.42	162.09	138.32	128.82	150.895	147.32
GAPDH	189.555	172.13	160.035	166.72	182.16	167.01	175.125	183.965
Beclin1/GAPDH	0.66669	0.92477	0.8087	0.97223	0.75933	0.77133	0.86164	0.8008
Beclin1	166.13	130.183	148.19	163.695	174.15	146.325	145.29	134.765
GAPDH	190.98	176.23	177.42	184.305	187.98	195.795	182.475	173.185
Beclin1/GAPDH	0.86988	0.73871	0.83525	0.88817	0.92643	0.74734	0.79622	0.77816
Beclin1	151.38	98.483	129.33	136.875				
GAPDH	175.315	167.445	173.535	165.63				
Beclin1/GAPDH	0.86347	0.58815	0.74527	0.82639				

Beclin1	174.94	167.715	167.79	163.82				
GAPDH	187.36	190.215	178.765	168.495				
Beclin1/GAPDH	0.93371	0.88171	0.93861	0.97225				
	1.09731	1.22291	1.38516	1.28809	0.94286	0.8918	0.98799	0.95167
	0.66669	0.92477	0.8087	0.97223	0.75933	0.77133	0.86164	0.8008
	0.86988	0.73871	0.83525	0.88817	0.92643	0.74734	0.79622	0.77816
	0.86347	0.58815	0.74527	0.82639				
	0.93371	0.88171	0.93861	0.97225				
mean	0.88621	0.86864	0.94359	0.99372	0.87621	0.80349	0.88195	0.84354
std	0.13841	0.23676	0.25703	0.17766	0.08292	0.06321	0.0796	0.07702
ttest vs control		0.909		0.746		0.380		0.650

FigS1e

	786-O		Caki-1		786-O		Caki-1	
	Vector	OE	Vector	OE	si-Contrc	si-FKBP5	si-Contrc	si-FKBP5
IKB	181.298	134.812	155.977	177.295	160.754	116.938	145.999	114.068
GAPDH	184.634	184.562	189.496	180.995	208.707	200.51	195.861	169.727
	0.98193	0.73044	0.82311	0.97956	0.77024	0.5832	0.74542	0.67207
C-P65	195.629	169.51	117.198	123.384	135.573	150.652	161.428	90.217
N-P65	165.261	109.835	81.946	103.152	158.689	122.109	109.172	136.69
N/C	1.18376	1.54331	1.43019	1.19614	0.85433	1.23375	1.47866	0.66001
IKB	131.205	112.382	151.965	194.175	118.5	179.73	187.425	152.415
GAPDH	156.03	168.425	171.655	181.08	142.96	153.535	160.54	154.07
	0.8409	0.66725	0.88529	1.07232	0.8289	1.17061	1.16747	0.98926
C-P65	128.3	73.89	81.899	81.743	85.132	128.536	125.954	114.474
N-P65	134.08	89.055	123.344	92.681	120.44	117.692	98.171	119.207
N/C	0.95689	0.82971	0.66399	0.88198	0.70684	1.09214	1.28301	0.9603
IKB	117.41	102.06	121.166	119.392	84.231	120.036	117.431	109.87
GAPDH	137.298	118.04	149.684	134.124	123.691	134.526	137.704	129.346
	0.85515	0.86462	0.80948	0.89016	0.68098	0.89229	0.85278	0.84943
C-P65	120.654	79.456	66.983	89.737	118.432	110.751	76.091	105.194
N-P65	112.78	90.21	89.26	97.571	113.86	126.71	77.26	107.173
N/C	1.06982	0.88079	0.75043	0.91971	1.04015	0.87405	0.98487	0.98153
IKB	0.98193	0.73044	0.82311	0.97956	0.77024	0.5832	0.74542	0.67207
	0.8409	0.66725	0.88529	1.07232	0.8289	1.17061	1.16747	0.98926
	0.85515	0.86462	0.80948	0.89016	0.68098	0.89229	0.85278	0.84943
mean	0.89266	0.75411	0.8393	0.98068	0.76004	0.88203	0.92189	0.83692
std	0.06339	0.08229	0.033	0.07437	0.06082	0.23992	0.1791	0.12979
ttest vs control		0.132		0.070		0.524		0.616
N/C	1.18376	1.54331	1.43019	1.19614	0.85433	1.23375	1.47866	0.66001
	0.95689	0.82971	0.66399	0.88198	0.70684	1.09214	1.28301	0.9603
	1.06982	0.88079	0.75043	0.91971	1.04015	0.87405	0.98487	0.98153
mean	1.07016	1.08461	0.9482	0.99928	0.86711	1.06665	1.24884	0.86728
std	0.09262	0.32503	0.34264	0.14005	0.13637	0.14795	0.20303	0.14682
ttest vs control		0.955		0.855		0.233		0.098

FigS5b

	si-Contrc	siATG7				
				3MA		
						Rapamycin
ATG7	226.34	27.611	220.738	241.965	230.172	201.421
LC3A	30.583	131.718	128.91	9.9	109.971	11.41

LC3B	201.344	57.427	192.393	65.466	205.071	229.839
GAPDH	219.668	205.989	215.887	220.296	223.011	226.642
ATG7-relative	1.03037	0.13404	1.02247	1.09836	1.03211	0.88872
LC3A-relative	0.13922	0.63944	0.59712	0.04494	0.49312	0.05034
LC3B-relative	0.91658	0.27879	0.89117	0.29717	0.91956	1.01411
ATG7	183.335	17.9472	169.968	217.769	163.422	191.35
LC3A	24.4664	129.084	86.3697	9.801	80.2788	7.4165
LC3B	151.008	37.3276	161.61	60.8834	141.499	153.992
GAPDH	169.144	177.151	177.027	184.207	198.551	167.317
ATG7-relative	1.0839	0.10131	0.96012	1.18219	0.82307	1.14364
LC3A-relative	0.14465	0.72867	0.48789	0.05321	0.40432	0.04433
LC3B-relative	0.89278	0.21071	0.91291	0.33052	0.71266	0.92036
ATG7	194.652	25.9543	211.908	229.867	184.138	191.35
LC3A	30.2772	108.009	95.3934	9.405	108.871	10.269
LC3B	163.089	42.496	146.219	50.4088	159.955	151.694
GAPDH	155.964	150.372	166.233	169.281	159.63	169.047
ATG7-relative	1.24806	0.1726	1.27477	1.3579	1.15353	1.13193
LC3A-relative	0.19413	0.71828	0.57385	0.05556	0.68202	0.06075
LC3B-relative	1.04568	0.28261	0.8796	0.29778	1.00204	0.89735
ATG7-relative	1.03037	0.13404	1.02247	1.09836	1.03211	0.88872
ATG7-relative	1.0839	0.10131	0.96012	1.18219	0.82307	1.14364
ATG7-relative	1.24806	0.1726	1.27477	1.3579	1.15353	1.13193
mean	1.12078	0.13598	1.08579	1.21282	1.0029	1.05476
std	0.09262	0.02914	0.13603	0.10815	0.13648	0.11751
		0.00014		0.35965		0.70469
LC3A-relative	0.13922	0.63944	0.59712	0.04494	0.49312	0.05034
LC3A-relative	0.14465	0.72867	0.48789	0.05321	0.40432	0.04433
LC3A-relative	0.19413	0.71828	0.57385	0.05556	0.68202	0.06075
LC3B-relative	0.91658	0.27879	0.89117	0.29717	0.91956	1.01411
LC3B-relative	0.89278	0.21071	0.91291	0.33052	0.71266	0.92036
LC3B-relative	1.04568	0.28261	0.8796	0.29778	1.00204	0.89735
LCB/A	6.58353	0.43598	1.49246	6.61273	1.86477	20.1436
LCB/A	6.17206	0.28917	1.87114	6.21196	1.76259	20.7635
LCB/A	5.38652	0.39345	1.5328	5.35979	1.46922	14.772
mean	6.04737	0.37287	1.63213	6.06149	1.69886	18.5597
std	0.49657	0.06168	0.16981	0.52246	0.16766	2.69023
		8.8E-05		0.00034		0.0009

FigS5c

	Caki-1		
	Vector	OE	OE+TIMP3
FKBP51	31.252	223.64	214.332
TIMP3	130.304	76.672	140.784
MMP7	66.494	192.862	26.826
MMP9	71.475	247.92	31.551
GAPDH	182.942	176.202	181.191
FKBP51-relative	0.17083	1.26923	1.18291
TIMP3-relative	0.71227	0.43514	0.77699
MMP7-relative	0.36347	1.09455	0.14805
MMP9-relative	0.3907	1.40702	0.17413
FKBP51	10.56	194.964	176.964
TIMP3	151.08	65.117	172.52
MMP7	79.615	169.22	25.718
MMP9	54.943	175.576	25.964

GAPDH	187.534	179.524	197.412
FKBP51-relative	0.05631	1.08601	0.89642
TIMP3-relative	0.80561	0.36272	0.87391
MMP7-relative	0.42454	0.9426	0.13028
MMP9-relative	0.29298	0.97801	0.13152
FKBP51	27.884	198.476	187.172
TIMP3	129.25	70.74	146.853
MMP7	83.697	181.694	51.568
MMP9	64.743	166.952	31.26
GAPDH	171.369	190.638	169.306
FKBP51-relative	0.16271	1.04111	1.10552
TIMP3-relative	0.75422	0.37107	0.86738
MMP7-relative	0.4884	0.95308	0.30458
MMP9-relative	0.3778	0.87575	0.18464
FKBP51-relative	0.17083	1.26923	1.18291
FKBP51-relative	0.05631	1.08601	0.89642
FKBP51-relative	0.16271	1.04111	1.10552
mean	0.12995	1.13211	1.06162
std	0.05218	0.09867	0.12101
ttest vs control		0.00022	0.00056
ttest vs OE			0.55785
TIMP3-relative	0.71227	0.43514	0.77699
TIMP3-relative	0.80561	0.36272	0.87391
TIMP3-relative	0.75422	0.37107	0.86738
mean	0.75737	0.38964	0.83943
std	0.03817	0.03235	0.04423
ttest vs control		0.00048	0.11794
ttest vs OE			0.00031
MMP7-relative	0.36347	1.09455	0.14805
MMP7-relative	0.42454	0.9426	0.13028
MMP7-relative	0.4884	0.95308	0.30458
mean	0.42547	0.99675	0.1943
std	0.05101	0.06929	0.07832
ttest vs control		0.00072	0.02495
ttest vs OE			0.00041
MMP9-relative	0.3907	1.40702	0.17413
MMP9-relative	0.29298	0.97801	0.13152
MMP9-relative	0.3778	0.87575	0.18464
mean	0.35382	1.08693	0.16343
std	0.04335	0.23016	0.02297
ttest vs control		0.01145	0.00537
ttest vs OE			0.00484

submitter_id.samples	age_at_init	file_uuid	tumor_stag	sample_type.samples
TCGA-BP-4355-01A	59	6E38F2F3-	stage iii	Primary Tumor
TCGA-BP-4355-11A	59	6E38F2F3-	stage iii	Solid Tissue Normal
TCGA-A3-3359-01A	82	8ED684EA	stage i	Primary Tumor
TCGA-A3-3359-11A	82	8ED684EA	stage i	Solid Tissue Normal
TCGA-B0-4838-01A	69	B4B69E12-	stage i	Primary Tumor
TCGA-B0-4838-11A	69	B4B69E12-	stage i	Solid Tissue Normal
TCGA-BP-5001-01A	43	28CD47F5	stage i	Primary Tumor
TCGA-BP-5001-11A	43	28CD47F5	stage i	Solid Tissue Normal
TCGA-BP-4801-01A	57	7AC264D0	stage i	Primary Tumor
TCGA-BP-4801-11A	57	7AC264D0	stage i	Solid Tissue Normal
TCGA-CJ-4639-01A	49	DDEF9B69	stage ii	Primary Tumor
TCGA-CJ-4639-11A	49	DDEF9B69	stage ii	Solid Tissue Normal
TCGA-CJ-4640-01A	49	B82F4FB4-	stage iii	Primary Tumor
TCGA-CJ-4640-11A	49	B82F4FB4-	stage iii	Solid Tissue Normal
TCGA-BP-4341-01A	67	801325C5-	stage iii	Primary Tumor
TCGA-BP-4341-11A	67	801325C5-	stage iii	Solid Tissue Normal
TCGA-B0-5691-01A	66	8E6F459A-	stage i	Primary Tumor
TCGA-B0-5691-11A	66	8E6F459A-	stage i	Solid Tissue Normal
TCGA-CJ-4892-01A	65	9693D38C	stage i	Primary Tumor
TCGA-CJ-4892-11A	65	9693D38C	stage i	Solid Tissue Normal
TCGA-AK-3454-01A	84	87DBE5C7	stage i	Primary Tumor
TCGA-B0-5703-01A	73	F5120146-	stage i	Primary Tumor
TCGA-B0-5703-11A	73	F5120146-	stage i	Solid Tissue Normal
TCGA-CW-5581-01A	44	00BE93A4-	stage i	Primary Tumor
TCGA-CW-5581-11A	44	00BE93A4-	stage i	Solid Tissue Normal
TCGA-B0-5117-01A	40	BCF55B79-	stage i	Primary Tumor
TCGA-B0-5117-11A	40	BCF55B79-	stage i	Solid Tissue Normal
TCGA-BP-4158-01A	69	86527E50-	stage i	Primary Tumor
TCGA-BP-4158-11A	69	86527E50-	stage i	Solid Tissue Normal
TCGA-BP-4329-01A	75	0FC44DE5	stage iii	Primary Tumor
TCGA-BP-4329-11A	75	0FC44DE5	stage iii	Solid Tissue Normal
TCGA-BP-5174-01A	45	7E4B9F3E-	stage i	Primary Tumor
TCGA-BP-5174-11A	45	7E4B9F3E-	stage i	Solid Tissue Normal
TCGA-B2-4098-01A	72	F2E3AED7	stage i	Primary Tumor
TCGA-B2-4098-11A	72	F2E3AED7	stage i	Solid Tissue Normal
TCGA-B0-5693-01A	47	3A496D52	stage i	Primary Tumor
TCGA-B0-5693-11A	47	3A496D52	stage i	Solid Tissue Normal
TCGA-BP-4354-01A	40	8EA49A12	stage iv	Primary Tumor
TCGA-BP-4354-11A	40	8EA49A12	stage iv	Solid Tissue Normal
TCGA-BP-5010-01A	63	12FD3F7E-	stage iii	Primary Tumor
TCGA-BP-5010-11A	63	12FD3F7E-	stage iii	Solid Tissue Normal
TCGA-DV-A4VX-01A	59	AA5E3D24	stage iv	Primary Tumor
TCGA-B0-5710-01A	57	4D4B0B96	stage i	Primary Tumor
TCGA-B0-5710-11A	57	4D4B0B96	stage i	Solid Tissue Normal
TCGA-BP-4784-01A	67	0DAEFB78	stage i	Primary Tumor
TCGA-BP-4784-11A	67	0DAEFB78	stage i	Solid Tissue Normal
TCGA-BP-4960-01A	46	CF470AEC	stage ii	Primary Tumor
TCGA-BP-4960-11A	46	CF470AEC	stage ii	Solid Tissue Normal
TCGA-B0-5088-01A	53	CA4DF249	stage i	Primary Tumor
TCGA-B0-5088-11A	53	CA4DF249	stage i	Solid Tissue Normal
TCGA-BP-5004-01A	53	9BFA460E-	stage i	Primary Tumor
TCGA-BP-5004-11A	53	9BFA460E-	stage i	Solid Tissue Normal
TCGA-AK-3430-01A	61	97C75092-	stage iii	Primary Tumor
TCGA-BP-5000-01A	40	9C004DA2	stage i	Primary Tumor
TCGA-BP-5000-11A	40	9C004DA2	stage i	Solid Tissue Normal
TCGA-BP-4340-01A	70	BD7009C9	stage i	Primary Tumor
TCGA-BP-4340-11A	70	BD7009C9	stage i	Solid Tissue Normal

TCGA-BP-5186-01A	50	A824B9C6	stage i	Primary Tumor
TCGA-BP-5186-11A	50	A824B9C6	stage i	Solid Tissue Normal
TCGA-B0-4849-01A	51	B012C1C8	stage iii	Primary Tumor
TCGA-B0-4849-11A	51	B012C1C8	stage iii	Solid Tissue Normal
TCGA-BP-5175-01A	60	66E1AF39·	stage i	Primary Tumor
TCGA-BP-5175-11A	60	66E1AF39·	stage i	Solid Tissue Normal
TCGA-B8-A8YJ-01A	60	5E54A488·	stage i	Primary Tumor
TCGA-B0-5075-01A	77	639C9C84	stage iii	Primary Tumor
TCGA-B0-5075-11A	77	639C9C84	stage iii	Solid Tissue Normal
TCGA-EU-5905-01A	67	7BF91284·	stage i	Primary Tumor
TCGA-DV-5566-01A	67	DBDB02ED	stage i	Primary Tumor
TCGA-CZ-5469-01A	41	68B15428·	stage ii	Primary Tumor
TCGA-CZ-5469-11A	41	68B15428·	stage ii	Solid Tissue Normal
TCGA-A3-3336-01A	75	A53E4458·	stage i	Primary Tumor
TCGA-A3-3336-11A	75	A53E4458·	stage i	Solid Tissue Normal
TCGA-B0-5116-01A	52	6228CE26·	stage iii	Primary Tumor
TCGA-B0-5116-11A	52	6228CE26·	stage iii	Solid Tissue Normal
TCGA-B0-5095-01A	81	1E244279·	stage iii	Primary Tumor
TCGA-B0-5095-11A	81	1E244279·	stage iii	Solid Tissue Normal
TCGA-CJ-5678-01A	62	A863EDDA	stage iv	Primary Tumor
TCGA-CJ-5678-11A	62	A863EDDA	stage iv	Solid Tissue Normal
TCGA-G6-A5PC-01A	54	336073F7·	stage iv	Primary Tumor
TCGA-A3-3374-01A	51	DE531716	stage i	Primary Tumor
TCGA-A3-3374-11A	51	DE531716	stage i	Solid Tissue Normal
TCGA-CJ-4884-01A	72	DAE46504	stage iii	Primary Tumor
TCGA-CJ-4884-11A	72	DAE46504	stage iii	Solid Tissue Normal
TCGA-B4-5832-01A	65	CAC4765E	stage iii	Primary Tumor
TCGA-BP-4335-01A	65	50CA5B51	stage iv	Primary Tumor
TCGA-BP-4335-11A	65	50CA5B51	stage iv	Solid Tissue Normal
TCGA-B0-4710-01A	75	65653168·	stage iii	Primary Tumor
TCGA-B0-4710-11A	75	65653168·	stage iii	Solid Tissue Normal
TCGA-A3-3370-01A	48	F070105D·	stage i	Primary Tumor
TCGA-A3-3370-11A	48	F070105D·	stage i	Solid Tissue Normal
TCGA-CW-5588-01A	78	1477CAB6	stage i	Primary Tumor
TCGA-CW-5588-11A	78	1477CAB6	stage i	Solid Tissue Normal
TCGA-BP-5181-01A	58	880B1659·	stage i	Primary Tumor
TCGA-BP-5181-11A	58	880B1659·	stage i	Solid Tissue Normal
TCGA-CZ-5453-01A	67	E529E32A·	stage ii	Primary Tumor
TCGA-CZ-5453-11A	67	E529E32A·	stage ii	Solid Tissue Normal
TCGA-EU-5907-01A	81	5AAADF1D	stage iii	Primary Tumor
TCGA-B8-5158-01A	56	D52B6099	stage iii	Primary Tumor
TCGA-CJ-6031-01A	54	9B1FEDC3	stage i	Primary Tumor
TCGA-CJ-6031-11A	54	9B1FEDC3	stage i	Solid Tissue Normal
TCGA-B0-4815-01A	65	7A393200·	stage iii	Primary Tumor
TCGA-B0-4815-11A	65	7A393200·	stage iii	Solid Tissue Normal
TCGA-DV-5565-01A	59	14156836·	stage i	Primary Tumor
TCGA-CZ-5986-01A	61	6D17AF7E	stage i	Primary Tumor
TCGA-CZ-5986-11A	61	6D17AF7E	stage i	Solid Tissue Normal
TCGA-CZ-5459-01A	63	F67FC842·	stage iii	Primary Tumor
TCGA-CZ-5459-01B	63	F67FC842·	stage iii	Primary Tumor
TCGA-CZ-5459-11A	63	F67FC842·	stage iii	Solid Tissue Normal
TCGA-BP-4160-01A	67	1B7B33E4·	stage iii	Primary Tumor
TCGA-BP-4160-11A	67	1B7B33E4·	stage iii	Solid Tissue Normal
TCGA-BP-5009-01A	52	5E0FCE94·	stage i	Primary Tumor
TCGA-BP-5009-11A	52	5E0FCE94·	stage i	Solid Tissue Normal
TCGA-A3-3380-01A	54	5B278272·	stage i	Primary Tumor
TCGA-A3-3380-11A	54	5B278272·	stage i	Solid Tissue Normal
TCGA-B2-5639-01A	46	4494D745	stage iv	Primary Tumor

TCGA-B2-5639-11A	46	4494D745	stage iv	Solid Tissue Normal
TCGA-BP-4769-01A	63	65BA6981	stage i	Primary Tumor
TCGA-BP-4769-11A	63	65BA6981	stage i	Solid Tissue Normal
TCGA-BP-4797-01A	34	89F92412-	stage iii	Primary Tumor
TCGA-BP-4797-11A	34	89F92412-	stage iii	Solid Tissue Normal
TCGA-CZ-5462-01A	83	BDC19515	stage iv	Primary Tumor
TCGA-CZ-5462-11A	83	BDC19515	stage iv	Solid Tissue Normal
TCGA-A3-3352-01A	74	B581CC3D	stage iii	Primary Tumor
TCGA-A3-3352-11A	74	B581CC3D	stage iii	Solid Tissue Normal
TCGA-CJ-4882-01A	57	E65DE8B5	stage iii	Primary Tumor
TCGA-CJ-4882-11A	57	E65DE8B5	stage iii	Solid Tissue Normal
TCGA-CJ-4881-01A	41	8F9247B0-	stage iii	Primary Tumor
TCGA-CJ-4881-11A	41	8F9247B0-	stage iii	Solid Tissue Normal
TCGA-BP-4771-01A	62	B601578C	stage iv	Primary Tumor
TCGA-BP-4771-11A	62	B601578C	stage iv	Solid Tissue Normal
TCGA-BP-5170-01A	55	52A3AC70	stage i	Primary Tumor
TCGA-BP-5170-11A	55	52A3AC70	stage i	Solid Tissue Normal
TCGA-BP-4760-01A	69	EF8ACCA2	stage i	Primary Tumor
TCGA-BP-4760-11A	69	EF8ACCA2	stage i	Solid Tissue Normal
TCGA-CZ-4865-01A	70	EADF8516	stage i	Primary Tumor
TCGA-CZ-4865-11A	70	EADF8516	stage i	Solid Tissue Normal
TCGA-CJ-4644-01A	48	321A1AFE	stage iv	Primary Tumor
TCGA-CJ-4644-11A	48	321A1AFE	stage iv	Solid Tissue Normal
TCGA-CZ-5464-01A	69	57EEABDE	stage iv	Primary Tumor
TCGA-CZ-5464-11A	69	57EEABDE	stage iv	Solid Tissue Normal
TCGA-CW-5587-01A	62	218A732A	stage iii	Primary Tumor
TCGA-CW-5587-11A	62	218A732A	stage iii	Solid Tissue Normal
TCGA-BP-4765-01A	43	37B7144F-	stage i	Primary Tumor
TCGA-BP-4765-11A	43	37B7144F-	stage i	Solid Tissue Normal
TCGA-B8-A54F-01A	49	70ADD31E	stage i	Primary Tumor
TCGA-B0-5713-01A	75	AE33AC1C	stage iii	Primary Tumor
TCGA-B0-5713-11A	75	AE33AC1C	stage iii	Solid Tissue Normal
TCGA-B4-5835-01A	64	65EDBEAE	stage i	Primary Tumor
TCGA-BP-4777-01A	46	96E4650A	stage i	Primary Tumor
TCGA-BP-4777-11A	46	96E4650A	stage i	Solid Tissue Normal
TCGA-DV-A4VZ-01A	53	941858F6-	stage i	Primary Tumor
TCGA-CJ-4635-01A	48	89EE70B8-	stage i	Primary Tumor
TCGA-CJ-4635-11B	48	89EE70B8-	stage i	Solid Tissue Normal
TCGA-B0-5696-01A	69	507D0844	stage iii	Primary Tumor
TCGA-B0-5696-11A	69	507D0844	stage iii	Solid Tissue Normal
TCGA-CZ-5982-01A	59	F27090BE-	stage i	Primary Tumor
TCGA-CZ-5982-11A	59	F27090BE-	stage i	Solid Tissue Normal
TCGA-CW-5583-01A	51	B2AE604B	stage i	Primary Tumor
TCGA-CW-5583-11A	51	B2AE604B	stage i	Solid Tissue Normal
TCGA-B0-5107-01A	65	4C53E6F9-	stage iv	Primary Tumor
TCGA-B0-5107-11A	65	4C53E6F9-	stage iv	Solid Tissue Normal
TCGA-GK-A6C7-01A	76	EC83E48D	stage i	Primary Tumor
TCGA-GK-A6C7-11A	76	EC83E48D	stage i	Solid Tissue Normal
TCGA-B0-4698-01A	75	C655446E	stage iv	Primary Tumor
TCGA-B0-4698-11A	75	C655446E	stage iv	Solid Tissue Normal
TCGA-BP-4353-01A	61	CE51450F-	stage i	Primary Tumor
TCGA-BP-4353-11A	61	CE51450F-	stage i	Solid Tissue Normal
TCGA-B0-5698-01A	77	C1933720	stage i	Primary Tumor
TCGA-A3-3308-01A	77	676B16DB	stage iii	Primary Tumor
TCGA-A3-3308-11A	77	676B16DB	stage iii	Solid Tissue Normal
TCGA-B8-4148-01A	63	62AEE695	stage i	Primary Tumor
TCGA-CJ-4893-01A	76	E26235CA	stage i	Primary Tumor
TCGA-CJ-4893-11A	76	E26235CA	stage i	Solid Tissue Normal

TCGA-B0-5097-01A	59	010CDFB6	stage iii	Primary Tumor
TCGA-B0-5097-11A	59	010CDFB6	stage iii	Solid Tissue Normal
TCGA-BP-4973-01A	47	611EC18C	stage iii	Primary Tumor
TCGA-BP-4973-11A	47	611EC18C	stage iii	Solid Tissue Normal
TCGA-B0-4697-01A	46	0C8DB6CF	stage iv	Primary Tumor
TCGA-B0-4697-11A	46	0C8DB6CF	stage iv	Solid Tissue Normal
TCGA-A3-3357-01A	62	D4ECC87E	stage ii	Primary Tumor
TCGA-A3-3357-11A	62	D4ECC87E	stage ii	Solid Tissue Normal
TCGA-A3-3335-01A	41	E7CEB30D	stage ii	Primary Tumor
TCGA-A3-3335-11A	41	E7CEB30D	stage ii	Solid Tissue Normal
TCGA-A3-3358-01A	57	FE86A6E3-	stage i	Primary Tumor
TCGA-A3-3358-11A	57	FE86A6E3-	stage i	Solid Tissue Normal
TCGA-AK-3431-01A	62	C8B657FD	stage ii	Primary Tumor
TCGA-A3-3349-01A	34	3D5BE25E	stage i	Primary Tumor
TCGA-A3-3349-11A	34	3D5BE25E	stage i	Solid Tissue Normal
TCGA-B0-5109-01A	69	DD6C66BF	stage iii	Primary Tumor
TCGA-B0-5109-11A	69	DD6C66BF	stage iii	Solid Tissue Normal
TCGA-BP-4768-01A	72	05DE3483	stage i	Primary Tumor
TCGA-BP-4768-11A	72	05DE3483	stage i	Solid Tissue Normal
TCGA-B8-4153-01B	74	B85A0799	stage iii	Primary Tumor
TCGA-B8-4153-11A	74	B85A0799	stage iii	Solid Tissue Normal
TCGA-B8-4620-01A	70	99527DB6	stage iii	Primary Tumor
TCGA-B8-4620-11A	70	99527DB6	stage iii	Solid Tissue Normal
TCGA-BP-4986-01A	75	8C374144-	stage i	Primary Tumor
TCGA-BP-4986-11A	75	8C374144-	stage i	Solid Tissue Normal
TCGA-CJ-4920-01A	64	8FB5DAE7	stage i	Primary Tumor
TCGA-CJ-4920-11A	64	8FB5DAE7	stage i	Solid Tissue Normal
TCGA-CJ-4873-01A	85	4371A155-	stage iii	Primary Tumor
TCGA-CJ-4873-11A	85	4371A155-	stage iii	Solid Tissue Normal
TCGA-AS-3778-01A	35	1D973708	stage i	Primary Tumor
TCGA-CJ-4637-01A	52	8CEA0A3D	stage iv	Primary Tumor
TCGA-CJ-4637-11A	52	8CEA0A3D	stage iv	Solid Tissue Normal
TCGA-B0-4713-01A	76	843A43EA	stage iii	Primary Tumor
TCGA-B0-4713-11A	76	843A43EA	stage iii	Solid Tissue Normal
TCGA-CZ-4853-01A	82	B431DD88	stage i	Primary Tumor
TCGA-CZ-4853-11A	82	B431DD88	stage i	Solid Tissue Normal
TCGA-B0-5706-01A	45	9A021EBA	stage ii	Primary Tumor
TCGA-B0-5706-11A	45	9A021EBA	stage ii	Solid Tissue Normal
TCGA-CJ-5683-01A	78	4AA2E63C	stage i	Primary Tumor
TCGA-CJ-5683-11A	78	4AA2E63C	stage i	Solid Tissue Normal
TCGA-AK-3425-01A	68	BEC7CC25	stage i	Primary Tumor
TCGA-B4-5838-01A	52	F7689846-	not report	Primary Tumor
TCGA-MM-A564-01A	68	D147F2E7-	stage ii	Primary Tumor
TCGA-BP-5199-01A	58	E7B00261-	stage ii	Primary Tumor
TCGA-BP-5199-11A	58	E7B00261-	stage ii	Solid Tissue Normal
TCGA-B0-5085-01A	76	B31199CD	stage iii	Primary Tumor
TCGA-B0-5085-11A	76	B31199CD	stage iii	Solid Tissue Normal
TCGA-AK-3433-01A	48	7F4F675B-	stage ii	Primary Tumor
TCGA-B0-4842-01A	73	02D2D305	stage iii	Primary Tumor
TCGA-B0-4842-11A	73	02D2D305	stage iii	Solid Tissue Normal
TCGA-B8-5545-01A	42	E63A02D2	stage i	Primary Tumor
TCGA-A3-3319-01A	70	DCDBF019	stage i	Primary Tumor
TCGA-A3-3319-11A	70	DCDBF019	stage i	Solid Tissue Normal
TCGA-CW-5585-01A	51	26DDB2B1	stage iv	Primary Tumor
TCGA-CW-5585-11A	51	26DDB2B1	stage iv	Solid Tissue Normal
TCGA-B0-5120-01A	72	DB47B1A5	stage i	Primary Tumor
TCGA-B0-5120-11A	72	DB47B1A5	stage i	Solid Tissue Normal
TCGA-CZ-4859-01A	59	3CD057A7	stage i	Primary Tumor

TCGA-CZ-4859-11A	59	3CD057A7	stage i	Solid Tissue Normal
TCGA-BP-4974-01A	58	04DB941C	stage iv	Primary Tumor
TCGA-BP-4974-11A	58	04DB941C	stage iv	Solid Tissue Normal
TCGA-A3-3320-01A	52	A164719D	stage i	Primary Tumor
TCGA-A3-3320-11A	52	A164719D	stage i	Solid Tissue Normal
TCGA-AK-3451-01A	48	6490EB2B-	stage ii	Primary Tumor
TCGA-BP-4332-01A	36	F45A6C85	stage iii	Primary Tumor
TCGA-BP-4332-11A	36	F45A6C85	stage iii	Solid Tissue Normal
TCGA-AK-3456-01A	48	6CB0989E-	stage ii	Primary Tumor
TCGA-CJ-4868-01A	42	F9BAC9A3	stage iv	Primary Tumor
TCGA-CJ-4868-11A	42	F9BAC9A3	stage iv	Solid Tissue Normal
TCGA-B2-3923-01A	59	91F40370-	stage ii	Primary Tumor
TCGA-B2-3923-01B	59	91F40370-	stage ii	Primary Tumor
TCGA-B2-3923-11A	59	91F40370-	stage ii	Solid Tissue Normal
TCGA-CJ-4900-01A	69	2CCDB9F3	stage iv	Primary Tumor
TCGA-CJ-4900-11A	69	2CCDB9F3	stage iv	Solid Tissue Normal
TCGA-B0-4833-01A	82	B8D03DB3	stage i	Primary Tumor
TCGA-B0-4833-11A	82	B8D03DB3	stage i	Solid Tissue Normal
TCGA-B0-4699-01A	74	153A7E77-	stage iv	Primary Tumor
TCGA-B0-4699-11A	74	153A7E77-	stage iv	Solid Tissue Normal
TCGA-CZ-5984-01A	51	749F1B5B-	stage i	Primary Tumor
TCGA-CZ-5984-11A	51	749F1B5B-	stage i	Solid Tissue Normal
TCGA-BP-5182-01A	56	754F0CCD	stage i	Primary Tumor
TCGA-BP-5182-11A	56	754F0CCD	stage i	Solid Tissue Normal
TCGA-B0-4837-01A	63	D79785D6	stage i	Primary Tumor
TCGA-B0-4837-11A	63	D79785D6	stage i	Solid Tissue Normal
TCGA-BP-4968-01A	40	48C4869C	stage i	Primary Tumor
TCGA-BP-4968-11A	40	48C4869C	stage i	Solid Tissue Normal
TCGA-CJ-5671-01A	51	35C4A56E	stage i	Primary Tumor
TCGA-CJ-5671-11A	51	35C4A56E	stage i	Solid Tissue Normal
TCGA-BP-4804-01A	59	DC0A75D2	stage i	Primary Tumor
TCGA-BP-4804-11A	59	DC0A75D2	stage i	Solid Tissue Normal
TCGA-AK-3443-01A	45	7D993EA8	stage ii	Primary Tumor
TCGA-B0-5812-01A	53	9F11B768-	stage i	Primary Tumor
TCGA-B0-5812-11A	53	9F11B768-	stage i	Solid Tissue Normal
TCGA-B8-4619-01A	58	E8209EA2-	stage i	Primary Tumor
TCGA-B8-4619-11A	58	E8209EA2-	stage i	Solid Tissue Normal
TCGA-B4-5834-01A	59	745CD1B1	stage i	Primary Tumor
TCGA-AK-3450-01A	85	A0E7B127-	stage i	Primary Tumor
TCGA-B0-5709-01A	62	9ACBF36F	stage iii	Primary Tumor
TCGA-B0-5709-11A	62	9ACBF36F	stage iii	Solid Tissue Normal
TCGA-B2-4102-01A	61	EBD1A20E	stage i	Primary Tumor
TCGA-B2-4102-11A	61	EBD1A20E	stage i	Solid Tissue Normal
TCGA-CW-6090-01A	68	2DF7FA48	stage i	Primary Tumor
TCGA-CW-6090-11A	68	2DF7FA48	stage i	Solid Tissue Normal
TCGA-BP-4977-01A	57	6C0EA353	stage i	Primary Tumor
TCGA-BP-4977-11A	57	6C0EA353	stage i	Solid Tissue Normal
TCGA-B0-5690-01A	53	6BFE5B58-	stage i	Primary Tumor
TCGA-B0-5690-11A	53	6BFE5B58-	stage i	Solid Tissue Normal
TCGA-B0-5121-01A	56	2B815745-	stage i	Primary Tumor
TCGA-B0-5121-11A	56	2B815745-	stage i	Solid Tissue Normal
TCGA-A3-3387-01A	49	A1BA3ED3	stage i	Primary Tumor
TCGA-A3-3387-11A	49	A1BA3ED3	stage i	Solid Tissue Normal
TCGA-AK-3445-01A	69	397D0918	stage iii	Primary Tumor
TCGA-CJ-4870-01A	58	8F06C14E-	stage iii	Primary Tumor
TCGA-CJ-4870-11A	58	8F06C14E-	stage iii	Solid Tissue Normal
TCGA-BP-4985-01A	72	F1FB12F1-	stage iii	Primary Tumor
TCGA-BP-4985-11A	72	F1FB12F1-	stage iii	Solid Tissue Normal

TCGA-CJ-5679-01A	73	849FAA68	stage iii	Primary Tumor
TCGA-CJ-5679-11A	73	849FAA68	stage iii	Solid Tissue Normal
TCGA-CJ-4905-01A	62	4DA840DE	stage i	Primary Tumor
TCGA-CJ-4905-11A	62	4DA840DE	stage i	Solid Tissue Normal
TCGA-AK-3455-01A	71	5DE1E471-	stage iii	Primary Tumor
TCGA-BP-4352-01A	74	30BB961D	stage iv	Primary Tumor
TCGA-BP-4352-11A	74	30BB961D	stage iv	Solid Tissue Normal
TCGA-BP-4972-01A	43	D38D8B3E	stage iii	Primary Tumor
TCGA-BP-4972-11A	43	D38D8B3E	stage iii	Solid Tissue Normal
TCGA-CZ-5452-01A	69	E9BD04A3	stage ii	Primary Tumor
TCGA-CZ-5452-11A	69	E9BD04A3	stage ii	Solid Tissue Normal
TCGA-B0-5092-01A	53	8BAFFBB7-	stage iv	Primary Tumor
TCGA-B0-5092-11A	53	8BAFFBB7-	stage iv	Solid Tissue Normal
TCGA-B8-5550-01A	71	AD1D8509	stage iii	Primary Tumor
TCGA-B0-5077-01A	77	F0382B68-	stage i	Primary Tumor
TCGA-B0-5077-11A	77	F0382B68-	stage i	Solid Tissue Normal
TCGA-CZ-5454-01A	63	83F2B00B-	stage iv	Primary Tumor
TCGA-CZ-5454-11A	63	83F2B00B-	stage iv	Solid Tissue Normal
TCGA-A3-A6NL-01A	49	3A6DD02F	stage i	Primary Tumor
TCGA-A3-A6NL-11A	49	3A6DD02F	stage i	Solid Tissue Normal
TCGA-BP-4162-01A	65	9860C2AD	stage i	Primary Tumor
TCGA-BP-4162-11A	65	9860C2AD	stage i	Solid Tissue Normal
TCGA-BP-5184-01A	54	5A2A5718	stage i	Primary Tumor
TCGA-BP-5184-11A	54	5A2A5718	stage i	Solid Tissue Normal
TCGA-CZ-5463-01A	76	674447EA-	stage ii	Primary Tumor
TCGA-CZ-5463-11A	76	674447EA-	stage ii	Solid Tissue Normal
TCGA-CZ-4856-01A	62	576ACDE3	stage i	Primary Tumor
TCGA-CZ-4856-11A	62	576ACDE3	stage i	Solid Tissue Normal
TCGA-B0-4818-01A	68	C1C9DFAE	stage ii	Primary Tumor
TCGA-B0-4818-11A	68	C1C9DFAE	stage ii	Solid Tissue Normal
TCGA-B8-4151-01A	51	809A8398-	stage iii	Primary Tumor
TCGA-A3-3385-01A	46	D5F0DC81	stage i	Primary Tumor
TCGA-A3-3385-11A	46	D5F0DC81	stage i	Solid Tissue Normal
TCGA-CW-5589-01A	52	688FDBA1	stage i	Primary Tumor
TCGA-CW-5589-11A	52	688FDBA1	stage i	Solid Tissue Normal
TCGA-B2-4101-01A	52	705A4203-	stage ii	Primary Tumor
TCGA-B2-4101-11A	52	705A4203-	stage ii	Solid Tissue Normal
TCGA-BP-4781-01A	78	7226D05A	stage i	Primary Tumor
TCGA-BP-4781-11A	78	7226D05A	stage i	Solid Tissue Normal
TCGA-CJ-6027-01A	77	F855F155-	stage i	Primary Tumor
TCGA-CJ-6027-11A	77	F855F155-	stage i	Solid Tissue Normal
TCGA-B0-5100-01A	72	9926DDE5	stage iii	Primary Tumor
TCGA-B0-5100-11A	72	9926DDE5	stage iii	Solid Tissue Normal
TCGA-MW-A4EC-01A	72	CE200E47-	stage i	Primary Tumor
TCGA-B0-5400-01A	59	3F2A0061-	stage iii	Primary Tumor
TCGA-B0-5400-11A	59	3F2A0061-	stage iii	Solid Tissue Normal
TCGA-B8-A54G-01A	50	2D2D35A1	stage i	Primary Tumor
TCGA-A3-3372-01A	64	D8403BAE	stage iii	Primary Tumor
TCGA-A3-3372-11A	64	D8403BAE	stage iii	Solid Tissue Normal
TCGA-AK-3427-01A	65	41FFCB3A	stage i	Primary Tumor
TCGA-CJ-4641-01A	55	A1DB3151	stage iv	Primary Tumor
TCGA-CJ-4641-11A	55	A1DB3151	stage iv	Solid Tissue Normal
TCGA-BP-4982-01A	42	DCE5C673	stage i	Primary Tumor
TCGA-BP-4982-11A	42	DCE5C673	stage i	Solid Tissue Normal
TCGA-BP-5008-01A	46	B0D743AD	stage i	Primary Tumor
TCGA-BP-5008-11A	46	B0D743AD	stage i	Solid Tissue Normal
TCGA-B2-5633-01A	56	F2924EC7-	stage i	Primary Tumor
TCGA-B2-5633-01B	56	F2924EC7-	stage i	Primary Tumor

TCGA-B2-5633-11A	56	F2924EC7·	stage i	Solid Tissue Normal
TCGA-6D-AA2E-01A	68	6FBD8CC0	stage i	Primary Tumor
TCGA-BP-4803-01A	79	1D01C119	stage iii	Primary Tumor
TCGA-BP-4803-11A	79	1D01C119	stage iii	Solid Tissue Normal
TCGA-CJ-4894-01A	58	ABC8DECE	stage iii	Primary Tumor
TCGA-CJ-4894-11A	58	ABC8DECE	stage iii	Solid Tissue Normal
TCGA-BP-5168-01A	75	E25E1BDC	stage i	Primary Tumor
TCGA-BP-5168-11A	75	E25E1BDC	stage i	Solid Tissue Normal
TCGA-BP-4789-01A	48	DF4F5D6E	stage i	Primary Tumor
TCGA-BP-4789-11A	48	DF4F5D6E	stage i	Solid Tissue Normal
TCGA-B0-4827-01A	77	D89D91BE	stage iii	Primary Tumor
TCGA-B0-4827-11A	77	D89D91BE	stage iii	Solid Tissue Normal
TCGA-CZ-5465-01A	76	D105F1F1·	stage iii	Primary Tumor
TCGA-CZ-5465-11A	76	D105F1F1·	stage iii	Solid Tissue Normal
TCGA-CJ-4643-01A	67	9D97AEEC	stage ii	Primary Tumor
TCGA-CJ-4643-11A	67	9D97AEEC	stage ii	Solid Tissue Normal
TCGA-BP-5177-01A	46	5157D53F·	stage i	Primary Tumor
TCGA-BP-5177-11A	46	5157D53F·	stage i	Solid Tissue Normal
TCGA-AK-3460-01A	58	276681E7-	stage i	Primary Tumor
TCGA-AK-3429-01A	54	F9F3C532-	stage ii	Primary Tumor
TCGA-CZ-5455-01A	63	336802A5·	stage iv	Primary Tumor
TCGA-CZ-5455-11A	63	336802A5·	stage iv	Solid Tissue Normal
TCGA-A3-3313-01A	59	E7B6C4D1	stage i	Primary Tumor
TCGA-A3-3313-11A	59	E7B6C4D1	stage i	Solid Tissue Normal
TCGA-CJ-4895-01A	62	C93A0E44	stage iv	Primary Tumor
TCGA-CJ-4895-11A	62	C93A0E44	stage iv	Solid Tissue Normal
TCGA-B4-5377-01A	68	E1808554-	stage iv	Primary Tumor
TCGA-A3-A8CQ-01A	59	A9902E52·	stage i	Primary Tumor
TCGA-BP-4166-01A	69	B131DDF0	stage iii	Primary Tumor
TCGA-BP-4166-11A	69	B131DDF0	stage iii	Solid Tissue Normal
TCGA-A3-3373-01A	54	BFFAC483	stage i	Primary Tumor
TCGA-A3-3373-11A	54	BFFAC483	stage i	Solid Tissue Normal
TCGA-CJ-5680-01A	65	57D0667A	stage iv	Primary Tumor
TCGA-CJ-5680-11A	65	57D0667A	stage iv	Solid Tissue Normal
TCGA-AK-3453-01A	58	0924CB60·	stage ii	Primary Tumor
TCGA-B0-5692-01A	66	1C63EAAE	stage iii	Primary Tumor
TCGA-B0-5692-11A	66	1C63EAAE	stage iii	Solid Tissue Normal
TCGA-B0-5697-01A	50	4E722D22	stage i	Primary Tumor
TCGA-B0-5697-11A	50	4E722D22	stage i	Solid Tissue Normal
TCGA-B8-5551-01A	65	9A5F0D90	stage i	Primary Tumor
TCGA-B8-5551-11A	65	9A5F0D90	stage i	Solid Tissue Normal
TCGA-BP-4787-01A	59	29E69EB2-	stage iv	Primary Tumor
TCGA-BP-4787-11A	59	29E69EB2-	stage iv	Solid Tissue Normal
TCGA-BP-4173-01A	47	05839304-	stage ii	Primary Tumor
TCGA-BP-4173-11A	47	05839304-	stage ii	Solid Tissue Normal
TCGA-B0-4816-01A	49	39C6AB02	stage ii	Primary Tumor
TCGA-B0-4816-11A	49	39C6AB02	stage ii	Solid Tissue Normal
TCGA-B4-5844-01A	61	2215127A·	stage ii	Primary Tumor
TCGA-BP-4343-01A	64	E7359698-	stage iii	Primary Tumor
TCGA-BP-4343-11A	64	E7359698-	stage iii	Solid Tissue Normal
TCGA-CZ-5456-01A	57	E082F834-	stage ii	Primary Tumor
TCGA-CZ-5456-11A	57	E082F834-	stage ii	Solid Tissue Normal
TCGA-B8-5549-01A	53	2C8845AB	stage i	Primary Tumor
TCGA-B8-5549-11A	53	2C8845AB	stage i	Solid Tissue Normal
TCGA-A3-A8OU-01A	74	6FC66EF4-	stage i	Primary Tumor
TCGA-BP-4969-01A	63	5DA88C36	stage i	Primary Tumor
TCGA-BP-4969-11A	63	5DA88C36	stage i	Solid Tissue Normal
TCGA-AK-3465-01A	71	8B2748AC	stage i	Primary Tumor

TCGA-CJ-4634-01A	60	D809A484	stage i	Primary Tumor
TCGA-CJ-4634-11A	60	D809A484	stage i	Solid Tissue Normal
TCGA-BP-5194-01A	39	21E1A6A5	stage i	Primary Tumor
TCGA-BP-5194-11A	39	21E1A6A5	stage i	Solid Tissue Normal
TCGA-CZ-4854-01A	68	7AB6F7D4	stage i	Primary Tumor
TCGA-CZ-4854-11A	68	7AB6F7D4	stage i	Solid Tissue Normal
TCGA-A3-3382-01A	69	48D32188	stage i	Primary Tumor
TCGA-A3-3382-11A	69	48D32188	stage i	Solid Tissue Normal
TCGA-BP-4962-01A	58	84F00E13-	stage ii	Primary Tumor
TCGA-BP-4962-11A	58	84F00E13-	stage ii	Solid Tissue Normal
TCGA-CJ-4874-01A	73	BAD661C5	stage i	Primary Tumor
TCGA-CJ-4874-11A	73	BAD661C5	stage i	Solid Tissue Normal
TCGA-B0-4712-01A	76	A1961A6D	stage iv	Primary Tumor
TCGA-B0-4712-11A	76	A1961A6D	stage iv	Solid Tissue Normal
TCGA-CW-5580-01A	73	FA611178-	stage iv	Primary Tumor
TCGA-CW-5580-11A	73	FA611178-	stage iv	Solid Tissue Normal
TCGA-BP-4964-01A	54	93E7ADDE	stage i	Primary Tumor
TCGA-BP-4964-11A	54	93E7ADDE	stage i	Solid Tissue Normal
TCGA-DV-5573-01A	41	1A522511-	stage i	Primary Tumor
TCGA-BP-5183-01A	57	64476D8B	stage iii	Primary Tumor
TCGA-BP-5183-11A	57	64476D8B	stage iii	Solid Tissue Normal
TCGA-CJ-4890-01A	72	A0A946DF	stage iv	Primary Tumor
TCGA-CJ-4890-11A	72	A0A946DF	stage iv	Solid Tissue Normal
TCGA-CJ-6033-01A	54	6AA88770	stage iv	Primary Tumor
TCGA-CJ-6033-11A	54	6AA88770	stage iv	Solid Tissue Normal
TCGA-B8-A7U6-01A	54	20430024-	stage i	Primary Tumor
TCGA-BP-4327-01A	75	5B92F22E-	stage ii	Primary Tumor
TCGA-BP-4327-11A	75	5B92F22E-	stage ii	Solid Tissue Normal
TCGA-T7-A92I-01A	47	33F82509-	stage i	Primary Tumor
TCGA-CJ-5684-01A	61	5DA1B9A(	stage iii	Primary Tumor
TCGA-CJ-5684-11A	61	5DA1B9A(	stage iii	Solid Tissue Normal
TCGA-CJ-5676-01A	47	68203929-	stage iii	Primary Tumor
TCGA-CJ-5676-11A	47	68203929-	stage iii	Solid Tissue Normal
TCGA-CZ-5467-01A	86	01F578BA-	stage iii	Primary Tumor
TCGA-CZ-5467-11A	86	01F578BA-	stage iii	Solid Tissue Normal
TCGA-BP-4993-01A	58	AD5E5CBA	stage i	Primary Tumor
TCGA-BP-4993-11A	58	AD5E5CBA	stage i	Solid Tissue Normal
TCGA-B0-5711-01A	50	B7AA1C8A	stage iii	Primary Tumor
TCGA-B0-5711-11A	50	B7AA1C8A	stage iii	Solid Tissue Normal
TCGA-CJ-4886-01A	42	4F040577-	stage i	Primary Tumor
TCGA-CJ-4886-11A	42	4F040577-	stage i	Solid Tissue Normal
TCGA-B8-5553-01A	67	91713F8E-	stage i	Primary Tumor
TCGA-B0-4945-01A	75	3ECE3665-	stage i	Primary Tumor
TCGA-B0-4945-11A	75	3ECE3665-	stage i	Solid Tissue Normal
TCGA-BP-5006-01A	61	C3F3B7A7	stage i	Primary Tumor
TCGA-BP-5006-11A	61	C3F3B7A7	stage i	Solid Tissue Normal
TCGA-B0-4810-01A	47	1010ED49	stage iii	Primary Tumor
TCGA-B0-4810-11A	47	1010ED49	stage iii	Solid Tissue Normal
TCGA-B0-5110-01A	71	16D36B3C	stage i	Primary Tumor
TCGA-B0-5110-11A	71	16D36B3C	stage i	Solid Tissue Normal
TCGA-BP-5176-01A	78	5614070B-	stage i	Primary Tumor
TCGA-BP-5176-11A	78	5614070B-	stage i	Solid Tissue Normal
TCGA-CZ-5985-01A	58	1E7199E5-	stage ii	Primary Tumor
TCGA-CZ-5985-11A	58	1E7199E5-	stage ii	Solid Tissue Normal
TCGA-DV-A4W0-01A	55	60DB23FA	stage i	Primary Tumor
TCGA-DV-A4W0-05A	55	60DB23FA	stage i	Additional - New Primary
TCGA-BP-4963-01A	63	B3DF16C4	stage i	Primary Tumor
TCGA-BP-4963-11A	63	B3DF16C4	stage i	Solid Tissue Normal

TCGA-BP-4177-01A	65	5F6F91D7·	stage i	Primary Tumor
TCGA-BP-4177-11A	65	5F6F91D7·	stage i	Solid Tissue Normal
TCGA-BP-4999-01A	56	859C1699·	stage i	Primary Tumor
TCGA-BP-4999-11A	56	859C1699·	stage i	Solid Tissue Normal
TCGA-G6-A8L8-01A	62	5590E1F5-	stage i	Primary Tumor
TCGA-BP-4967-01A	76	65AEC5F5	stage iii	Primary Tumor
TCGA-BP-4967-11A	76	65AEC5F5	stage iii	Solid Tissue Normal
TCGA-A3-3378-01A	60	24478731-	stage i	Primary Tumor
TCGA-A3-3378-11A	60	24478731-	stage i	Solid Tissue Normal
TCGA-CJ-4878-01A	71	6070A677·	stage iii	Primary Tumor
TCGA-CJ-4878-11A	71	6070A677·	stage iii	Solid Tissue Normal
TCGA-CJ-6030-01A	65	BB63ADBE	stage i	Primary Tumor
TCGA-CJ-6030-11A	65	BB63ADBE	stage i	Solid Tissue Normal
TCGA-B8-5163-01A	63	105A0995·	stage iii	Primary Tumor
TCGA-BP-4976-01A	77	374DE081	stage i	Primary Tumor
TCGA-BP-4976-11A	77	374DE081	stage i	Solid Tissue Normal
TCGA-BP-5192-01A	59	25402B33-	stage i	Primary Tumor
TCGA-BP-5192-11A	59	25402B33-	stage i	Solid Tissue Normal
TCGA-B0-5108-01A	54	DB4F3F13·	stage iii	Primary Tumor
TCGA-B0-5108-11A	54	DB4F3F13·	stage iii	Solid Tissue Normal
TCGA-A3-3307-01A	66	61BA85DD	stage iii	Primary Tumor
TCGA-A3-3307-11A	66	61BA85DD	stage iii	Solid Tissue Normal
TCGA-BP-4169-01A	76	47B5943E-	stage ii	Primary Tumor
TCGA-BP-4169-11A	76	47B5943E-	stage ii	Solid Tissue Normal
TCGA-CJ-4916-01A	69	9AE3875A	stage iii	Primary Tumor
TCGA-CJ-4916-11A	69	9AE3875A	stage iii	Solid Tissue Normal
TCGA-BP-4337-01A	76	D9499138	stage iii	Primary Tumor
TCGA-BP-4337-11A	76	D9499138	stage iii	Solid Tissue Normal
TCGA-CZ-4863-01A	51	0264DB4B	stage iii	Primary Tumor
TCGA-CZ-4863-11A	51	0264DB4B	stage iii	Solid Tissue Normal
TCGA-BP-4988-01A	72	12A14F55·	stage i	Primary Tumor
TCGA-BP-4988-11A	72	12A14F55·	stage i	Solid Tissue Normal
TCGA-BP-4989-01A	58	CCD1B378	stage iii	Primary Tumor
TCGA-BP-4989-11A	58	CCD1B378	stage iii	Solid Tissue Normal
TCGA-B0-4844-01A	60	D586DDC:	stage iv	Primary Tumor
TCGA-B0-4844-11A	60	D586DDC:	stage iv	Solid Tissue Normal
TCGA-CJ-4638-01A	46	A85252FF-	stage iv	Primary Tumor
TCGA-CJ-4638-11A	46	A85252FF-	stage iv	Solid Tissue Normal
TCGA-BP-5190-01A	61	D1D099D6	stage i	Primary Tumor
TCGA-BP-5190-11A	61	D1D099D6	stage i	Solid Tissue Normal
TCGA-CZ-5458-01A	43	351193C9·	stage iii	Primary Tumor
TCGA-CZ-5458-11A	43	351193C9·	stage iii	Solid Tissue Normal
TCGA-A3-A8OW-01A	37	9C696615·	stage iii	Primary Tumor
TCGA-B0-4693-01A	72	0A2094B0	stage iii	Primary Tumor
TCGA-B0-4693-11A	72	0A2094B0	stage iii	Solid Tissue Normal
TCGA-B0-4696-01A	58	14D72D07	stage iii	Primary Tumor
TCGA-B0-4696-11A	58	14D72D07	stage iii	Solid Tissue Normal
TCGA-BP-4795-01A	74	34B6AF90·	stage i	Primary Tumor
TCGA-BP-4795-11A	74	34B6AF90·	stage i	Solid Tissue Normal
TCGA-CJ-5681-01A	44	BB8137DC	stage iv	Primary Tumor
TCGA-CJ-5681-11A	44	BB8137DC	stage iv	Solid Tissue Normal
TCGA-B2-5641-01A	79	B392B44F-	stage i	Primary Tumor
TCGA-B2-5641-11A	79	B392B44F-	stage i	Solid Tissue Normal
TCGA-BP-4762-01A	42	9B76C5F9·	stage i	Primary Tumor
TCGA-BP-4762-11A	42	9B76C5F9·	stage i	Solid Tissue Normal
TCGA-BP-4782-01A	55	1845FAA3	stage i	Primary Tumor
TCGA-BP-4782-11A	55	1845FAA3	stage i	Solid Tissue Normal
TCGA-B8-5552-01B	41	1E511B0D	stage i	Primary Tumor

TCGA-B8-5552-11A	41	1E511B0D	stage i	Solid Tissue Normal
TCGA-B4-5843-01A	45	EF08679B-	stage i	Primary Tumor
TCGA-BP-4330-01A	60	9B1D4994	stage iii	Primary Tumor
TCGA-BP-4330-11A	60	9B1D4994	stage iii	Solid Tissue Normal
TCGA-B2-3924-01A	73	21BDACD!	stage i	Primary Tumor
TCGA-B2-3924-01B	73	21BDACD!	stage i	Primary Tumor
TCGA-B2-3924-11A	73	21BDACD!	stage i	Solid Tissue Normal
TCGA-BP-4770-01A	73	01DDABA!	stage iv	Primary Tumor
TCGA-BP-4770-11A	73	01DDABA!	stage iv	Solid Tissue Normal
TCGA-B0-5084-01A	33	FC08008C	stage iv	Primary Tumor
TCGA-B0-5084-11A	33	FC08008C	stage iv	Solid Tissue Normal
TCGA-CJ-4903-01A	50	27320BDD	stage i	Primary Tumor
TCGA-CJ-4903-11A	50	27320BDD	stage i	Solid Tissue Normal
TCGA-B0-5083-01A	63	9374C37F-	stage i	Primary Tumor
TCGA-B0-5083-11A	63	9374C37F-	stage i	Solid Tissue Normal
TCGA-BP-4790-01A	76	FEEC1411-	stage i	Primary Tumor
TCGA-BP-4790-11A	76	FEEC1411-	stage i	Solid Tissue Normal
TCGA-BP-5195-01A	75	6F000783-	stage i	Primary Tumor
TCGA-BP-5195-11A	75	6F000783-	stage i	Solid Tissue Normal
TCGA-B2-4099-01A	83	6CC7741F	stage i	Primary Tumor
TCGA-B2-4099-11A	83	6CC7741F	stage i	Solid Tissue Normal
TCGA-BP-5198-01A	72	8E6035A5-	stage iii	Primary Tumor
TCGA-BP-5198-11A	72	8E6035A5-	stage iii	Solid Tissue Normal
TCGA-CJ-4636-01A	51	39A98B04	stage iii	Primary Tumor
TCGA-CJ-4636-11A	51	39A98B04	stage iii	Solid Tissue Normal
TCGA-B0-5705-01A	65	C6E2CCB2	stage i	Primary Tumor
TCGA-B0-5705-11A	65	C6E2CCB2	stage i	Solid Tissue Normal
TCGA-B0-5402-01A	64	9859AAB1	stage iv	Primary Tumor
TCGA-B0-5402-11A	64	9859AAB1	stage iv	Solid Tissue Normal
TCGA-BP-5007-01A	45	726D96E8	stage ii	Primary Tumor
TCGA-BP-5007-11A	45	726D96E8	stage ii	Solid Tissue Normal
TCGA-B0-4822-01A	78	576A562B	stage ii	Primary Tumor
TCGA-B0-4822-11A	78	576A562B	stage ii	Solid Tissue Normal
TCGA-BP-4776-01A	52	17EC41B4-	stage i	Primary Tumor
TCGA-BP-4776-11A	52	17EC41B4-	stage i	Solid Tissue Normal
TCGA-A3-A8OX-01A	65	418C5AA0	stage i	Primary Tumor
TCGA-B0-5712-01A	68	DF076FBB	stage iv	Primary Tumor
TCGA-B0-5712-11A	68	DF076FBB	stage iv	Solid Tissue Normal
TCGA-A3-3347-01A	76	79609DDA	stage iii	Primary Tumor
TCGA-A3-3347-11A	76	79609DDA	stage iii	Solid Tissue Normal
TCGA-CJ-4901-01A	47	E7BB4D90	stage iii	Primary Tumor
TCGA-CJ-4901-11A	47	E7BB4D90	stage iii	Solid Tissue Normal
TCGA-BP-4161-01A	74	E9E17E98-	stage i	Primary Tumor
TCGA-BP-4161-11A	74	E9E17E98-	stage i	Solid Tissue Normal
TCGA-BP-4981-01A	75	53BFF5F9-	stage iii	Primary Tumor
TCGA-BP-4981-11A	75	53BFF5F9-	stage iii	Solid Tissue Normal
TCGA-BP-4971-01A	40	3E01826E-	stage iii	Primary Tumor
TCGA-BP-4971-11A	40	3E01826E-	stage iii	Solid Tissue Normal
TCGA-A3-3324-01A	51	2AEE1CCA	stage i	Primary Tumor
TCGA-A3-3324-11A	51	2AEE1CCA	stage i	Solid Tissue Normal
TCGA-B0-5080-01A	63	842F7DF0-	stage iv	Primary Tumor
TCGA-B0-5080-11A	63	842F7DF0-	stage iv	Solid Tissue Normal
TCGA-A3-3329-01A	75	5C36490A	stage i	Primary Tumor
TCGA-A3-3329-11A	75	5C36490A	stage i	Solid Tissue Normal
TCGA-CJ-5677-01A	54	DB0C3B61	stage iv	Primary Tumor
TCGA-CJ-5677-11A	54	DB0C3B61	stage iv	Solid Tissue Normal
TCGA-A3-3343-01A	79	26B1F579-	stage ii	Primary Tumor
TCGA-A3-3343-11A	79	26B1F579-	stage ii	Solid Tissue Normal

TCGA-A3-3376-01A	51	86959679-	stage i	Primary Tumor
TCGA-A3-3376-11A	51	86959679-	stage i	Solid Tissue Normal
TCGA-BP-4325-01A	64	844D0293	stage i	Primary Tumor
TCGA-BP-4325-11A	64	844D0293	stage i	Solid Tissue Normal
TCGA-B0-5700-01A	77	9B497369-	stage i	Primary Tumor
TCGA-B0-5700-11A	77	9B497369-	stage i	Solid Tissue Normal
TCGA-CJ-4908-01A	38	AE2561EF-	stage i	Primary Tumor
TCGA-CJ-4908-11A	38	AE2561EF-	stage i	Solid Tissue Normal
TCGA-CJ-4875-01A	67	DA015C1A	stage iv	Primary Tumor
TCGA-CJ-4875-11A	67	DA015C1A	stage iv	Solid Tissue Normal
TCGA-EU-5906-01A	55	547C5432-	stage i	Primary Tumor
TCGA-3Z-A93Z-01A	69	4F1F919B-	stage i	Primary Tumor
TCGA-B0-5119-01A	61	0E52DD30	stage i	Primary Tumor
TCGA-B0-5119-11A	61	0E52DD30	stage i	Solid Tissue Normal
TCGA-CZ-5989-01A	60	A37C32D9	stage ii	Primary Tumor
TCGA-CZ-5989-11A	60	A37C32D9	stage ii	Solid Tissue Normal
TCGA-B0-4823-01A	88	2BB56013-	stage i	Primary Tumor
TCGA-B0-4823-11A	88	2BB56013-	stage i	Solid Tissue Normal
TCGA-B0-5399-01A	46	13F689C8-	stage i	Primary Tumor
TCGA-B8-A54J-01A	60	48B70B15-	stage ii	Primary Tumor
TCGA-EU-5904-01A	47	9BCDF452	stage i	Primary Tumor
TCGA-CJ-4907-01A	58	FCB95DC9	stage iii	Primary Tumor
TCGA-CJ-4907-11A	58	FCB95DC9	stage iii	Solid Tissue Normal
TCGA-CZ-4862-01A	46	6BD0D739	stage i	Primary Tumor
TCGA-CZ-4862-11A	46	6BD0D739	stage i	Solid Tissue Normal
TCGA-CJ-4897-01A	79	565B48E3-	stage iii	Primary Tumor
TCGA-CJ-4897-11A	79	565B48E3-	stage iii	Solid Tissue Normal
TCGA-BP-4346-01A	57	F47E395F-	stage iii	Primary Tumor
TCGA-BP-4346-11A	57	F47E395F-	stage iii	Solid Tissue Normal
TCGA-CJ-4913-01A	45	AC0E57A7	stage iii	Primary Tumor
TCGA-CJ-4913-11A	45	AC0E57A7	stage iii	Solid Tissue Normal
TCGA-CW-6087-01A	61	DF03535B	stage iv	Primary Tumor
TCGA-CW-6087-11A	61	DF03535B	stage iv	Solid Tissue Normal
TCGA-BP-4775-01A	55	46C145B7-	stage i	Primary Tumor
TCGA-BP-4775-11A	55	46C145B7-	stage i	Solid Tissue Normal
TCGA-AK-3436-01A	40	37A82018-	stage iv	Primary Tumor
TCGA-BP-4174-01A	49	98A344DC	stage ii	Primary Tumor
TCGA-BP-4174-11A	49	98A344DC	stage ii	Solid Tissue Normal
TCGA-CJ-4885-01A	64	5C1782D0	stage iv	Primary Tumor
TCGA-CJ-4885-11A	64	5C1782D0	stage iv	Solid Tissue Normal
TCGA-BP-4995-01A	68	9C5AE1F9	stage i	Primary Tumor
TCGA-BP-4995-11A	68	9C5AE1F9	stage i	Solid Tissue Normal
TCGA-B0-5102-01A	74	B4A20CD9	stage i	Primary Tumor
TCGA-B0-5102-11A	74	B4A20CD9	stage i	Solid Tissue Normal
TCGA-CZ-5466-01A	67	527A731D	stage iii	Primary Tumor
TCGA-CZ-5466-11A	67	527A731D	stage iii	Solid Tissue Normal
TCGA-BP-5202-01A	75	F93D440E-	stage iii	Primary Tumor
TCGA-BP-5202-11A	75	F93D440E-	stage iii	Solid Tissue Normal
TCGA-BP-5178-01A	71	DFC4197E	stage iv	Primary Tumor
TCGA-BP-5178-11A	71	DFC4197E	stage iv	Solid Tissue Normal
TCGA-BP-4759-01A	50	A211EBB7	stage i	Primary Tumor
TCGA-BP-4759-11A	50	A211EBB7	stage i	Solid Tissue Normal
TCGA-B0-4714-01A	81	E1D2DD05	stage iv	Primary Tumor
TCGA-B0-4714-11A	81	E1D2DD05	stage iv	Solid Tissue Normal
TCGA-BP-4165-01A	64	BB84A373	stage i	Primary Tumor
TCGA-BP-4165-11A	64	BB84A373	stage i	Solid Tissue Normal
TCGA-CW-6093-01A	73	0C0950D1	stage i	Primary Tumor
TCGA-CW-6093-11A	73	0C0950D1	stage i	Solid Tissue Normal

TCGA-A3-3316-01A	57	5D215B1A	stage ii	Primary Tumor
TCGA-A3-3316-11A	57	5D215B1A	stage ii	Solid Tissue Normal
TCGA-B0-4845-01A	70	8F63B8D1	stage iv	Primary Tumor
TCGA-B0-4845-11A	70	8F63B8D1	stage iv	Solid Tissue Normal
TCGA-BP-5169-01A	70	2257D391	stage i	Primary Tumor
TCGA-BP-5169-11A	70	2257D391	stage i	Solid Tissue Normal
TCGA-CZ-4857-01A	56	5A045492-	stage iv	Primary Tumor
TCGA-CZ-4857-11A	56	5A045492-	stage iv	Solid Tissue Normal
TCGA-A3-3383-01A	52	AA334AFA	stage i	Primary Tumor
TCGA-A3-3383-11A	52	AA334AFA	stage i	Solid Tissue Normal
TCGA-CW-6096-01A	44	54F801B7-	stage i	Primary Tumor
TCGA-CW-6096-11A	44	54F801B7-	stage i	Solid Tissue Normal
TCGA-B0-4828-01A	79	581120DE	stage iv	Primary Tumor
TCGA-B0-4828-11A	79	581120DE	stage iv	Solid Tissue Normal
TCGA-B8-4621-01A	63	50C5BE3D	stage i	Primary Tumor
TCGA-CZ-4858-01A	39	B2CF5B64-	stage ii	Primary Tumor
TCGA-CZ-4858-11A	39	B2CF5B64-	stage ii	Solid Tissue Normal
TCGA-CJ-4904-01A	60	7F945C64-	stage iv	Primary Tumor
TCGA-CJ-4904-11A	60	7F945C64-	stage iv	Solid Tissue Normal
TCGA-CJ-4869-01A	49	D01A9999	stage iii	Primary Tumor
TCGA-CJ-4869-11A	49	D01A9999	stage iii	Solid Tissue Normal
TCGA-A3-3365-01A	46	37F1B581-	stage i	Primary Tumor
TCGA-A3-3365-11A	46	37F1B581-	stage i	Solid Tissue Normal
TCGA-DV-5569-01A	29	3C71C771	stage i	Primary Tumor
TCGA-B8-A54K-01A	61	C850DE09	stage i	Primary Tumor
TCGA-A3-A6NN-01A	78	5A9DCEE0	stage i	Primary Tumor
TCGA-DV-5567-01A	40	AE4E4CC3	stage i	Primary Tumor
TCGA-CJ-4912-01A	61	FB6644B1-	stage ii	Primary Tumor
TCGA-CJ-4912-11A	61	FB6644B1-	stage ii	Solid Tissue Normal
TCGA-BP-4992-01A	66	B1382791-	stage i	Primary Tumor
TCGA-BP-4992-11A	66	B1382791-	stage i	Solid Tissue Normal
TCGA-B0-4688-01A	46	5105DD37	stage iv	Primary Tumor
TCGA-B0-4688-11A	46	5105DD37	stage iv	Solid Tissue Normal
TCGA-B0-4814-01A	58	76B3E98F-	stage iv	Primary Tumor
TCGA-B0-4814-11A	58	76B3E98F-	stage iv	Solid Tissue Normal
TCGA-BP-4959-01A	49	8EF7E50A-	stage i	Primary Tumor
TCGA-BP-4959-11A	49	8EF7E50A-	stage i	Solid Tissue Normal
TCGA-BP-5189-01A	60	E8CBD80C	stage i	Primary Tumor
TCGA-BP-5189-11A	60	E8CBD80C	stage i	Solid Tissue Normal
TCGA-B0-4813-01A	68	F5C9CD91	stage iii	Primary Tumor
TCGA-B0-4813-11A	68	F5C9CD91	stage iii	Solid Tissue Normal
TCGA-CJ-5675-01A	70	A2B255FF-	stage ii	Primary Tumor
TCGA-CJ-5675-11A	70	A2B255FF-	stage ii	Solid Tissue Normal
TCGA-B0-4811-01A	48	A836E5F1-	stage iii	Primary Tumor
TCGA-B0-4811-11A	48	A836E5F1-	stage iii	Solid Tissue Normal
TCGA-B2-A4SR-01A	61	81EF0B48-	stage ii	Primary Tumor
TCGA-B2-A4SR-11A	61	81EF0B48-	stage ii	Solid Tissue Normal
TCGA-B0-4821-01A	68	75E3035F-	stage iii	Primary Tumor
TCGA-B0-4821-11A	68	75E3035F-	stage iii	Solid Tissue Normal
TCGA-A3-3331-01A	86	686CC653	stage i	Primary Tumor
TCGA-A3-3331-11A	86	686CC653	stage i	Solid Tissue Normal
TCGA-BP-4766-01A	43	7DC7086F	stage i	Primary Tumor
TCGA-BP-4766-11A	43	7DC7086F	stage i	Solid Tissue Normal
TCGA-B0-5699-01A	53	71D5F1F6-	stage i	Primary Tumor
TCGA-B0-5699-11A	53	71D5F1F6-	stage i	Solid Tissue Normal
TCGA-B0-4847-01A	60	ACF37644	stage iv	Primary Tumor
TCGA-B0-4847-11A	60	ACF37644	stage iv	Solid Tissue Normal
TCGA-CZ-5457-01A	62	CAB9F7F2	stage iii	Primary Tumor

TCGA-CZ-5457-11A	62	CAB9F7F2	stage iii	Solid Tissue Normal
TCGA-BP-5201-01A	63	E5906328-	stage iv	Primary Tumor
TCGA-BP-5201-11A	63	E5906328-	stage iv	Solid Tissue Normal
TCGA-A3-3322-01A	51	DEA7E510	stage i	Primary Tumor
TCGA-A3-3322-11A	51	DEA7E510	stage i	Solid Tissue Normal
TCGA-B8-A54E-01A	62	86CA2E91	stage i	Primary Tumor
TCGA-B0-4690-01A	65	9455B63F-	stage iv	Primary Tumor
TCGA-B0-4690-11A	65	9455B63F-	stage iv	Solid Tissue Normal
TCGA-BP-4349-01A	68	B2695AA5	stage i	Primary Tumor
TCGA-BP-4349-11A	68	B2695AA5	stage i	Solid Tissue Normal
TCGA-BP-4991-01A	54	191054EE-	stage i	Primary Tumor
TCGA-BP-4991-11A	54	191054EE-	stage i	Solid Tissue Normal
TCGA-CW-5584-01A	74	9BAE0333	stage iii	Primary Tumor
TCGA-CW-5584-11A	74	9BAE0333	stage iii	Solid Tissue Normal
TCGA-CZ-5468-01A	84	871ED6DD	stage iv	Primary Tumor
TCGA-CZ-5468-11A	84	871ED6DD	stage iv	Solid Tissue Normal
TCGA-CZ-4866-01A	79	F59D1DA6	stage i	Primary Tumor
TCGA-CZ-4866-11A	79	F59D1DA6	stage i	Solid Tissue Normal
TCGA-B0-4848-01A	54	8270F9EC-	stage iii	Primary Tumor
TCGA-B0-4848-11A	54	8270F9EC-	stage iii	Solid Tissue Normal
TCGA-B8-4146-01B	41	07875774-	stage i	Primary Tumor
TCGA-BP-4351-01A	51	6DECA3D9	stage iii	Primary Tumor
TCGA-BP-4351-11A	51	6DECA3D9	stage iii	Solid Tissue Normal
TCGA-A3-3363-01A	50	76D177F0	stage ii	Primary Tumor
TCGA-A3-3363-11A	50	76D177F0	stage ii	Solid Tissue Normal
TCGA-CZ-5451-01A	74	267CD74C	stage ii	Primary Tumor
TCGA-CZ-5451-11A	74	267CD74C	stage ii	Solid Tissue Normal
TCGA-BP-5191-01A	79	DB4F05EC	stage iii	Primary Tumor
TCGA-BP-5191-11A	79	DB4F05EC	stage iii	Solid Tissue Normal
TCGA-CZ-5461-01A	52	9FFEE890-	stage iv	Primary Tumor
TCGA-CZ-5461-11A	52	9FFEE890-	stage iv	Solid Tissue Normal
TCGA-BP-4756-01A	62	90BE0503-	stage i	Primary Tumor
TCGA-BP-4756-11A	62	90BE0503-	stage i	Solid Tissue Normal
TCGA-A3-3325-01A	52	ACFBAF77	stage i	Primary Tumor
TCGA-A3-3325-11A	52	ACFBAF77	stage i	Solid Tissue Normal
TCGA-BP-4799-01A	70	004CC4C9	stage iii	Primary Tumor
TCGA-BP-4799-11A	70	004CC4C9	stage iii	Solid Tissue Normal
TCGA-A3-3311-01A	57	323A1E4B	stage i	Primary Tumor
TCGA-A3-3311-11A	57	323A1E4B	stage i	Solid Tissue Normal
TCGA-CW-6088-01A	60	602D16C5	stage i	Primary Tumor
TCGA-CW-6088-11A	60	602D16C5	stage i	Solid Tissue Normal
TCGA-CJ-4918-01A	64	F11DEEF2-	stage iv	Primary Tumor
TCGA-CJ-4918-11A	64	F11DEEF2-	stage iv	Solid Tissue Normal
TCGA-CW-5590-01A	51	562AC200	stage iv	Primary Tumor
TCGA-CW-5590-11A	51	562AC200	stage iv	Solid Tissue Normal
TCGA-BP-4994-01A	54	065AD737	stage i	Primary Tumor
TCGA-BP-4994-11A	54	065AD737	stage i	Solid Tissue Normal
TCGA-DV-5568-01A	26	712B9F53-	stage i	Primary Tumor
TCGA-B2-5636-01A	79	EAFE502E-	stage i	Primary Tumor
TCGA-B2-5636-11A	79	EAFE502E-	stage i	Solid Tissue Normal
TCGA-BP-4970-01A	44	4C580DA5	stage iii	Primary Tumor
TCGA-BP-4970-11A	44	4C580DA5	stage iii	Solid Tissue Normal
TCGA-B0-4707-01A	63	F8E2D53F-	stage iii	Primary Tumor
TCGA-B0-4707-11A	63	F8E2D53F-	stage iii	Solid Tissue Normal
TCGA-BP-4807-01A	42	CA30D0D5	stage i	Primary Tumor
TCGA-BP-4807-11A	42	CA30D0D5	stage i	Solid Tissue Normal
TCGA-B8-4143-01A	66	8C7B303B	stage iv	Primary Tumor
TCGA-B8-4143-11A	66	8C7B303B	stage iv	Solid Tissue Normal

TCGA-BP-4344-01A	75	C8CF95F4	stage i	Primary Tumor
TCGA-BP-4344-11A	75	C8CF95F4	stage i	Solid Tissue Normal
TCGA-B0-5081-01A	79	F53307AF-	stage iii	Primary Tumor
TCGA-B0-5081-11A	79	F53307AF-	stage iii	Solid Tissue Normal
TCGA-CJ-4642-01B	47	784E90D5	stage ii	Primary Tumor
TCGA-CJ-4642-11A	47	784E90D5	stage ii	Solid Tissue Normal
TCGA-CJ-6032-01A	63	C734A0B4	stage ii	Primary Tumor
TCGA-CJ-6032-11A	63	C734A0B4	stage ii	Solid Tissue Normal
TCGA-B0-4839-01A	80	98293F9F-	stage i	Primary Tumor
TCGA-B0-4839-11A	80	98293F9F-	stage i	Solid Tissue Normal
TCGA-BP-5173-01A	75	25B9412B	stage i	Primary Tumor
TCGA-BP-5173-11A	75	25B9412B	stage i	Solid Tissue Normal
TCGA-CJ-4923-01A	63	3CBE402B	stage iv	Primary Tumor
TCGA-CJ-4923-11A	63	3CBE402B	stage iv	Solid Tissue Normal
TCGA-B8-A54D-01A	69	66198674-	stage iii	Primary Tumor
TCGA-B0-5695-01A	61	A9010CBB	stage i	Primary Tumor
TCGA-B0-5695-11A	61	A9010CBB	stage i	Solid Tissue Normal
TCGA-AK-3440-01A	58	5BB409A5	stage i	Primary Tumor
TCGA-B0-4819-01A	60	CAC5929F	stage iv	Primary Tumor
TCGA-B0-4819-11A	60	CAC5929F	stage iv	Solid Tissue Normal
TCGA-B0-5115-01A	43	7D9B2FE0	stage iv	Primary Tumor
TCGA-B0-5115-11A	43	7D9B2FE0	stage iv	Solid Tissue Normal
TCGA-AK-3447-01A	83	52A6954C	stage ii	Primary Tumor
TCGA-B0-4703-01A	51	C884FCD2	stage iv	Primary Tumor
TCGA-B0-4703-11A	51	C884FCD2	stage iv	Solid Tissue Normal
TCGA-BP-4761-01A	57	CAE63DE1	stage iii	Primary Tumor
TCGA-BP-4761-11A	57	CAE63DE1	stage iii	Solid Tissue Normal
TCGA-B0-5098-01A	53	D25E224F	stage i	Primary Tumor
TCGA-B0-5098-11A	53	D25E224F	stage i	Solid Tissue Normal
TCGA-AK-3434-01A	72	9410B4FF-	stage i	Primary Tumor
TCGA-MM-A563-01A	41	9649330F-	not report	Primary Tumor
TCGA-DV-5576-01A	55	E746D55D	stage i	Primary Tumor
TCGA-A3-3306-01A	67	674F19CE-	stage i	Primary Tumor
TCGA-A3-3306-11A	67	674F19CE-	stage i	Solid Tissue Normal
TCGA-AS-3777-01A	63	8C71D3FA	stage i	Primary Tumor
TCGA-CJ-4871-01A	63	0CE3E462-	stage iv	Primary Tumor
TCGA-CJ-4871-11A	63	0CE3E462-	stage iv	Solid Tissue Normal
TCGA-B0-4846-01A	52	28AB0428	stage iv	Primary Tumor
TCGA-B0-4846-11A	52	28AB0428	stage iv	Solid Tissue Normal
TCGA-A3-3351-01A	42	40654E68-	stage ii	Primary Tumor
TCGA-A3-3351-11A	42	40654E68-	stage ii	Solid Tissue Normal
TCGA-B0-4817-01A	81	5CFC3FFE	stage iii	Primary Tumor
TCGA-B0-4817-11A	81	5CFC3FFE	stage iii	Solid Tissue Normal
TCGA-B0-5104-01A	90	6DA1A8F5	stage i	Primary Tumor
TCGA-B0-5104-11A	90	6DA1A8F5	stage i	Solid Tissue Normal
TCGA-MM-A84U-01A	58	16EB2A38	stage i	Primary Tumor
TCGA-BP-4987-01A	41	04053C28	stage i	Primary Tumor
TCGA-BP-4987-11A	41	04053C28	stage i	Solid Tissue Normal
TCGA-B0-5099-01A	88	8C8C74CF	stage iii	Primary Tumor
TCGA-B0-5099-11A	88	8C8C74CF	stage iii	Solid Tissue Normal
TCGA-BP-5185-01A	56	AE28E3DF	stage i	Primary Tumor
TCGA-BP-5185-11A	56	AE28E3DF	stage i	Solid Tissue Normal
TCGA-BP-4983-01A	67	1DC0B82F	stage iii	Primary Tumor
TCGA-BP-4983-11A	67	1DC0B82F	stage iii	Solid Tissue Normal
TCGA-CJ-6028-01A	58	10EFDE0A	stage iv	Primary Tumor
TCGA-CJ-6028-11A	58	10EFDE0A	stage iv	Solid Tissue Normal
TCGA-B0-5707-01A	39	F64DDA52	stage i	Primary Tumor
TCGA-B0-5707-11A	39	F64DDA52	stage i	Solid Tissue Normal

TCGA-AK-3458-01A	48	EC350189·	stage i	Primary Tumor
TCGA-BP-4774-01A	57	E63E2223-	stage i	Primary Tumor
TCGA-BP-4774-11A	57	E63E2223-	stage i	Solid Tissue Normal
TCGA-B0-5702-01A	71	91051A51·	stage i	Primary Tumor
TCGA-B0-5702-11A	71	91051A51·	stage i	Solid Tissue Normal
TCGA-CJ-5689-01A	90	D97F09C9	stage i	Primary Tumor
TCGA-CJ-5689-11A	90	D97F09C9	stage i	Solid Tissue Normal
TCGA-A3-3346-01A	68	447DD1E0	stage i	Primary Tumor
TCGA-A3-3346-11A	68	447DD1E0	stage i	Solid Tissue Normal
TCGA-B0-4843-01A	57	709CB9BD	stage iii	Primary Tumor
TCGA-B0-4843-11A	57	709CB9BD	stage iii	Solid Tissue Normal
TCGA-CJ-4891-01A	57	65388DE6	stage iii	Primary Tumor
TCGA-CJ-4891-11A	57	65388DE6	stage iii	Solid Tissue Normal
TCGA-BP-4342-01A	79	99288D9D	stage ii	Primary Tumor
TCGA-BP-4342-11A	79	99288D9D	stage ii	Solid Tissue Normal
TCGA-DV-5575-01A	52	2BDE9E2B	stage i	Primary Tumor
TCGA-BP-4164-01A	51	DC5F1C1A	stage iii	Primary Tumor
TCGA-BP-4164-11A	51	DC5F1C1A	stage iii	Solid Tissue Normal
TCGA-CZ-5470-01A	72	A5F4D616	stage ii	Primary Tumor
TCGA-CZ-5470-11A	72	A5F4D616	stage ii	Solid Tissue Normal
TCGA-B4-5836-01A	61	581F871E-	stage i	Primary Tumor
TCGA-B0-4836-01A	61	9166AC70	stage iv	Primary Tumor
TCGA-B0-4836-11A	61	9166AC70	stage iv	Solid Tissue Normal
TCGA-B8-5159-01A	61	D8661989	stage i	Primary Tumor
TCGA-A3-3328-01A	79	07AAEED2	stage i	Primary Tumor
TCGA-A3-3328-11A	79	07AAEED2	stage i	Solid Tissue Normal
TCGA-G6-A8L7-01A	81	4E030E25-	stage i	Primary Tumor
TCGA-BP-4334-01A	56	2A7E0D6C	stage iii	Primary Tumor
TCGA-BP-4334-11A	56	2A7E0D6C	stage iii	Solid Tissue Normal
TCGA-A3-A6NI-01A	47	0C1B9A59	stage i	Primary Tumor
TCGA-BP-4347-01A	74	6025C5E6·	stage iii	Primary Tumor
TCGA-BP-4347-11A	74	6025C5E6·	stage iii	Solid Tissue Normal
TCGA-A3-A8OV-01A	75	A3EE7CD4	stage i	Primary Tumor
TCGA-B0-4701-01A	66	73A9CB34	stage iv	Primary Tumor
TCGA-B0-4701-11A	66	73A9CB34	stage iv	Solid Tissue Normal
TCGA-CJ-4872-01A	51	EAC3CDE7	stage i	Primary Tumor
TCGA-CJ-4872-11A	51	EAC3CDE7	stage i	Solid Tissue Normal
TCGA-B0-4691-01A	55	BFC7C8E4	stage iv	Primary Tumor
TCGA-B0-4691-11A	55	BFC7C8E4	stage iv	Solid Tissue Normal
TCGA-CZ-5987-01A	60	089D9FF7·	stage iv	Primary Tumor
TCGA-CZ-5987-11A	60	089D9FF7·	stage iv	Solid Tissue Normal
TCGA-B0-5113-01A	69	D8E8B737	stage iii	Primary Tumor
TCGA-B0-5113-11A	69	D8E8B737	stage iii	Solid Tissue Normal
TCGA-DV-5574-01A	37	043A95C6	stage i	Primary Tumor
TCGA-CJ-4888-01A	59	8EAB8470	stage iv	Primary Tumor
TCGA-CJ-4888-11A	59	8EAB8470	stage iv	Solid Tissue Normal
TCGA-BP-4975-01A	40	3ABE1F82·	stage i	Primary Tumor
TCGA-BP-4975-11A	40	3ABE1F82·	stage i	Solid Tissue Normal
TCGA-A3-A6NJ-01A	57	4D0A5832	stage i	Primary Tumor
TCGA-A3-A6NJ-11A	57	4D0A5832	stage i	Solid Tissue Normal
TCGA-BP-4345-01A	62	14E0B4A9	stage iii	Primary Tumor
TCGA-BP-4345-11A	62	14E0B4A9	stage iii	Solid Tissue Normal
TCGA-BP-4163-01A	60	1C7404A5	stage iii	Primary Tumor
TCGA-BP-4163-11A	60	1C7404A5	stage iii	Solid Tissue Normal
TCGA-B0-5694-01A	71	7770DDF9	stage iii	Primary Tumor
TCGA-B0-5694-11A	71	7770DDF9	stage iii	Solid Tissue Normal
TCGA-CJ-4902-01A	61	888C52D7	stage iii	Primary Tumor
TCGA-CJ-4902-11A	61	888C52D7	stage iii	Solid Tissue Normal

TCGA-CJ-5686-01A	59	FD23E0DE	stage i	Primary Tumor
TCGA-CJ-5686-11A	59	FD23E0DE	stage i	Solid Tissue Normal
TCGA-CJ-4889-01A	63	68B33C61	stage i	Primary Tumor
TCGA-CJ-4889-11A	63	68B33C61	stage i	Solid Tissue Normal
TCGA-B0-5094-01A	62	1D1C08D/	stage iv	Primary Tumor
TCGA-B0-5094-11A	62	1D1C08D/	stage iv	Solid Tissue Normal
TCGA-BP-4998-01A	49	CEA3FA4E	stage i	Primary Tumor
TCGA-BP-4998-11A	49	CEA3FA4E	stage i	Solid Tissue Normal
TCGA-AK-3461-01A	72	F6296F3B-	stage i	Primary Tumor
TCGA-BP-5200-01A	44	222B487E-	stage ii	Primary Tumor
TCGA-BP-5200-11A	44	222B487E-	stage ii	Solid Tissue Normal
TCGA-BP-4961-01A	47	5C538845	stage i	Primary Tumor
TCGA-BP-4961-11A	47	5C538845	stage i	Solid Tissue Normal
TCGA-CJ-4899-01A	42	D0841496	stage i	Primary Tumor
TCGA-CJ-4899-11A	42	D0841496	stage i	Solid Tissue Normal
TCGA-BP-5180-01A	53	DDA0808/	stage i	Primary Tumor
TCGA-BP-5180-11A	53	DDA0808/	stage i	Solid Tissue Normal
TCGA-B4-5378-01A	62	E3055773-	stage i	Primary Tumor
TCGA-B2-5635-01A	74	A4B19949	stage i	Primary Tumor
TCGA-B2-5635-01B	74	A4B19949	stage i	Primary Tumor
TCGA-B2-5635-11A	74	A4B19949	stage i	Solid Tissue Normal
TCGA-B0-4824-01A	49	3F833AAD	stage i	Primary Tumor
TCGA-B0-4824-11A	49	3F833AAD	stage i	Solid Tissue Normal
TCGA-B8-A54H-01A	69	DEBB8651	stage ii	Primary Tumor
TCGA-B0-4706-01A	61	C6C5F0BA	stage iii	Primary Tumor
TCGA-B0-4706-11A	61	C6C5F0BA	stage iii	Solid Tissue Normal
TCGA-B0-4700-01A	60	2123016E-	stage iv	Primary Tumor
TCGA-B0-4700-11A	60	2123016E-	stage iv	Solid Tissue Normal
TCGA-CJ-5672-01A	84	431E5DBA	stage i	Primary Tumor
TCGA-CJ-5672-11A	84	431E5DBA	stage i	Solid Tissue Normal
TCGA-BP-5196-01A	53	BB338A38	stage i	Primary Tumor
TCGA-BP-5196-11A	53	BB338A38	stage i	Solid Tissue Normal
TCGA-B0-4718-01A	57	86C6039C	stage iii	Primary Tumor
TCGA-B0-4718-11A	57	86C6039C	stage iii	Solid Tissue Normal
TCGA-BP-4338-01A	43	E4808241-	stage i	Primary Tumor
TCGA-BP-4338-11A	43	E4808241-	stage i	Solid Tissue Normal
TCGA-B0-5096-01A	72	D87CD84/	stage iii	Primary Tumor
TCGA-B0-5096-11A	72	D87CD84/	stage iii	Solid Tissue Normal
TCGA-B0-5701-01A	65	FADF7515	stage iii	Primary Tumor
TCGA-B0-5701-11A	65	FADF7515	stage iii	Solid Tissue Normal
TCGA-AK-3426-01A	37	BE0EB10E-	stage iii	Primary Tumor
TCGA-CZ-4864-01A	86	18800994-	stage ii	Primary Tumor
TCGA-CZ-4864-11A	86	18800994-	stage ii	Solid Tissue Normal
TCGA-BP-4159-01A	70	3340E1B7-	stage i	Primary Tumor
TCGA-BP-4159-11A	70	3340E1B7-	stage i	Solid Tissue Normal
TCGA-G6-A8L6-01A	55	B7DA85FB	stage iv	Primary Tumor
TCGA-BP-4176-01A	64	BBFA9C54	stage i	Primary Tumor
TCGA-BP-4176-11A	64	BBFA9C54	stage i	Solid Tissue Normal
TCGA-BP-5187-01A	54	75D914A2	stage i	Primary Tumor
TCGA-BP-5187-11A	54	75D914A2	stage i	Solid Tissue Normal
TCGA-B8-5165-01A	43	062D5D78	stage i	Primary Tumor
TCGA-B8-4154-01A	73	CF0D8B11	stage i	Primary Tumor
TCGA-B8-4154-11A	73	CF0D8B11	stage i	Solid Tissue Normal
TCGA-CW-5591-01A	56	811F8463-	stage iv	Primary Tumor
TCGA-CW-5591-11A	56	811F8463-	stage iv	Solid Tissue Normal
TCGA-CW-6097-01A	32	4E7F6263-	stage iii	Primary Tumor
TCGA-CW-6097-11A	32	4E7F6263-	stage iii	Solid Tissue Normal
TCGA-BP-4798-01A	74	49B05307-	not report	Primary Tumor

TCGA-BP-4798-11A	74	49B05307·	not report	Solid Tissue Normal
TCGA-A3-3317-01A	67	3682A43B	stage ii	Primary Tumor
TCGA-A3-3317-11A	67	3682A43B	stage ii	Solid Tissue Normal
TCGA-BP-4763-01A	79	A438311A	stage i	Primary Tumor
TCGA-BP-4763-11A	79	A438311A	stage i	Solid Tissue Normal
TCGA-A3-3367-01A	72	9141A95E·	stage i	Primary Tumor
TCGA-A3-3367-11A	72	9141A95E·	stage i	Solid Tissue Normal
TCGA-AK-3428-01A	62	FBD31E2D	stage iii	Primary Tumor
TCGA-CJ-4876-01A	57	1075C3F8·	stage ii	Primary Tumor
TCGA-CJ-4876-11A	57	1075C3F8·	stage ii	Solid Tissue Normal
TCGA-B0-4841-01A	63	07D2CEF3	stage iv	Primary Tumor
TCGA-B0-4841-11A	63	07D2CEF3	stage iv	Solid Tissue Normal
TCGA-A3-3362-01A	60	C9DDA27!	stage i	Primary Tumor
TCGA-A3-3362-11A	60	C9DDA27!	stage i	Solid Tissue Normal
TCGA-BP-4326-01A	53	F7AEA1DF	stage i	Primary Tumor
TCGA-BP-4326-11A	53	F7AEA1DF	stage i	Solid Tissue Normal
TCGA-B8-4622-01A	57	32615A0E·	stage iv	Primary Tumor
TCGA-B8-4622-11A	57	32615A0E·	stage iv	Solid Tissue Normal
TCGA-CJ-5682-01A	60	19DF5D8A	stage iv	Primary Tumor
TCGA-CJ-5682-11A	60	19DF5D8A	stage iv	Solid Tissue Normal
TCGA-B8-5546-01A	38	251C78B4·	stage i	Primary Tumor
TCGA-B8-5546-11A	38	251C78B4·	stage i	Solid Tissue Normal
TCGA-BP-4965-01A	46	F68A3B9E·	stage i	Primary Tumor
TCGA-BP-4965-11A	46	F68A3B9E·	stage i	Solid Tissue Normal
TCGA-B0-4834-01A	49	A45DFA5C	stage i	Primary Tumor
TCGA-B0-4834-11A	49	A45DFA5C	stage i	Solid Tissue Normal
TCGA-B8-A54I-01A	48	8BD715B1	stage i	Primary Tumor
TCGA-B8-5164-01A	65	E4BDDBA4	stage iii	Primary Tumor
TCGA-BP-4170-01A	72	7FDFC042	stage i	Primary Tumor
TCGA-BP-4170-11A	72	7FDFC042	stage i	Solid Tissue Normal
TCGA-B0-4852-01A	78	3B3DABB3	stage ii	Primary Tumor
TCGA-B0-4852-11A	78	3B3DABB3	stage ii	Solid Tissue Normal
TCGA-CZ-4860-01A	60	60AAD00D	stage iv	Primary Tumor
TCGA-CZ-4860-11A	60	60AAD00D	stage iv	Solid Tissue Normal
TCGA-CZ-5460-01A	55	D73718FA	stage iv	Primary Tumor
TCGA-CZ-5460-11A	55	D73718FA	stage iv	Solid Tissue Normal
TCGA-CJ-4887-01A	48	9FD032A9	stage iv	Primary Tumor
TCGA-CJ-4887-11A	48	9FD032A9	stage iv	Solid Tissue Normal
TCGA-AK-3444-01A	80	39C58017·	stage i	Primary Tumor
TCGA-A3-3326-01A	47	950DBF0A	stage i	Primary Tumor
TCGA-A3-3326-11A	47	950DBF0A	stage i	Solid Tissue Normal
TCGA-BP-4331-01A	52	EF57FD4C	stage i	Primary Tumor
TCGA-BP-4331-11A	52	EF57FD4C	stage i	Solid Tissue Normal
TCGA-CZ-5988-01A	38	09B19160·	stage i	Primary Tumor
TCGA-CZ-5988-11A	38	09B19160·	stage i	Solid Tissue Normal
TCGA-BP-4758-01A	40	39903A51·	stage i	Primary Tumor
TCGA-BP-4758-11A	40	39903A51·	stage i	Solid Tissue Normal
TCGA-B0-4694-01A	72	F555FE25-	stage iii	Primary Tumor
TCGA-B0-4694-11A	72	F555FE25-	stage iii	Solid Tissue Normal
TCGA-BP-4167-01A	59	E6DDED2F	stage iii	Primary Tumor
TCGA-BP-4167-11A	59	E6DDED2F	stage iii	Solid Tissue Normal
TCGA-CZ-4861-01A	63	D358877A	stage ii	Primary Tumor
TCGA-CZ-4861-11A	63	D358877A	stage ii	Solid Tissue Normal
TCGA-B0-5106-01A	64	5DBE4855	stage i	Primary Tumor
TCGA-B0-5106-11A	64	5DBE4855	stage i	Solid Tissue Normal
TCGA-A3-3323-01A	53	3BACDB13	stage i	Primary Tumor
TCGA-A3-3323-11A	53	3BACDB13	stage i	Solid Tissue Normal
TCGA-B8-5162-01A	62	9A711E1A	stage ii	Primary Tumor

Beroukhim Renal (70)

Normal	ccRCC	NH-ccRCC
-1.16861	-0.73548	-0.98303
-0.87408	-0.70889	-0.1451
-0.66259	0.12086	-0.64969
0.06811	0.70295	-0.09241
0.39049	0.80682	-0.07387
0.45376	0.86637	0.15381
0.46651	1.05535	0.49006
0.52855	1.08504	0.54164
0.80978	1.34317	0.9166
1.01504	1.43923	1.01831
1.99609	1.53474	1.10238
	1.62469	1.18433
	1.62865	1.20118
	1.67905	1.3107
	1.71256	1.39855
	1.74264	1.47335
	1.91729	1.48779
	1.9273	1.54279
	1.94985	1.88975
	2.02729	1.99744
	2.12151	2.18313
	2.12659	2.18795
	2.25504	2.7127
	2.27503	2.72448
	2.30007	3.01951
	2.48928	3.99361
	2.75654	4.62231
	3.27212	
	3.34871	
	3.7992	
	3.79922	
	4.04549	

PMID: 19470766

Yusenko

Normal	ccRCC
-1.50492	-1.16107
-0.47125	-0.91156
-0.39463	-0.89977
	-0.88707
	-0.83503
	-0.75804
	-0.71042
	-0.51635
	-0.34794
	-0.26551
	-0.22378
	0.1699
	-0.16408
	-0.15666
	-0.11671
	-0.10376
	-0.06845
	-0.05178
	0.0048
	0.2482
	0.30311
	0.33197
	0.33757
	0.51407

PMID: 19445733
